# IL-2–inducible T cell kinase deficiency sustains chimeric antigen receptor T cell therapy against tumor cells

**DOI:** 10.1172/JCI178558

**Published:** 2024-11-26

**Authors:** Zheng Fu, Zineng Huang, Hao Xu, Qingbai Liu, Jing Li, Keqing Song, Yating Deng, Yujia Tao, Huifang Zhang, Peilong Wang, Heng Li, Yue Sheng, Aijun Zhou, Lianbin Han, Yan Fu, Chenzhi Wang, Saurav Kumar Choudhary, Kaixiong Ye, Gianluca Veggiani, Zhihong Li, Avery August, Weishan Huang, Qiang Shan, Hongling Peng

**Affiliations:** 1 Department of Hematology, The Second Xiangya Hospital, Central South University, Changsha, Hunan, China.; 2 Hubei Jiangxia Laboratory, Wuhan, Hubei, China.; 3 MegaRobo Technologies Co. Ltd., Suzhou, China.; 4 Xinyi Biotech Co. Ltd., Lingang, Shanghai, China.; 5 Institute of Hematology, Central South University, Changsha, Hunan, China.; 6 Hunan Engineering Research Center of Cell Immunotherapy for Hematopoietic Malignancies, Changsha, Hunan, China.; 7 National Key Laboratory of Immunity and Inflammation, Suzhou Institute of Systems Medicine, Chinese Academy of Medical Sciences & Peking Union Medical College, Suzhou, Jiangsu, China.; 8 Lianshui People’s Hospital of Kangda College Affiliated to Nanjing Medical University, Huai’an, Jiangsu Province, China.; 9 Key Laboratory of Cluster Science, Ministry of Education of China, School of Chemistry and Chemical Engineering, Beijing Institute of Technology, Beijing, China.; 10 Tianjin Mogenetics Biotech Co. Ltd., Tianjin, China.; 11 Institute of Bioinformatics and; 12 Department of Genetics, Franklin College of Arts and Sciences, University of Georgia, Athens, Georgia, USA.; 13 Department of Pathobiological Sciences, School of Veterinary Medicine, Louisiana State University, Baton Rouge, Louisiana, USA.; 14 Department of Orthopedics, The Second Xiangya Hospital, Central South University, Changsha, China.; 15 Hunan Key Laboratory of Tumor Models and Individualized Medicine, Changsha, Hunan, China.; 16 Department of Microbiology and Immunology, College of Veterinary Medicine, Cornell University, Ithaca, New York, USA.

**Keywords:** Hematology, Immunology, Cancer immunotherapy, Leukemias, T cells

## Abstract

Despite the revolutionary achievements of chimeric antigen receptor (CAR) T cell therapy in treating cancers, especially leukemia, several key challenges still limit its therapeutic efficacy. Of particular relevance is the relapse of cancer in large part as a result of exhaustion and short persistence of CAR-T cells in vivo. IL-2–inducible T cell kinase (ITK) is a critical modulator of the strength of T cell receptor signaling, while its role in CAR signaling is unknown. By electroporation of CRISPR-associated protein 9 (Cas9) ribonucleoprotein (RNP) complex into CAR-T cells, we successfully deleted *ITK* in CD19-CAR-T cells with high efficiency. Bulk and single-cell RNA sequencing analyses revealed downregulation of exhaustion and upregulation of memory gene signatures in ITK-deficient CD19-CAR-T cells. Our results further demonstrated a significant reduction of T cell exhaustion and enhancement of T cell memory, with significant improvement of CAR-T cell expansion and persistence both in vitro and in vivo. Moreover, ITK-deficient CD19-CAR-T cells showed better control of tumor relapse. Our work provides a promising strategy of targeting ITK to develop sustainable CAR-T cell products for clinical use.

## Abstract 2

ITK deficiency decreases exhaustion and increases memory in CD19-CAR-T cells, resulting in improvement of CAR-T cell expansion and better control of tumor relapse.

## Introduction

Chimeric antigen receptor (CAR) T cell therapy, as an innovative cellular immunotherapy, has been used in leukemia (1), solid tumors (2), and several other diseases such as autoimmunity (3, 4) and cardiac injury (5). Currently, a total of nine CAR-T cell products have been approved for clinical therapy. Despite the remarkable clinical efficacy of CAR-T cell therapy in leukemia, especially in B cell malignancy (1), there are still many challenges limiting its therapeutic efficacy. Relapse of tumor remains the major obstacle to be addressed, especially in CD19-targeted CAR-T cell therapy, with which even about 40%–60% of patients who achieve a complete response eventually experience relapse (1, 6, 7). For example, a recent study reported that CAR-T cell treatment for diffuse large B cell lymphoma showed about 43% progression 8 months after CAR-T cell infusion (8). The efficacy of CAR-T cell therapy remains largely to be improved in cancer treatment.

Severe T cell exhaustion in the tumor environment and the relatively short persistence of CAR-T cells in vivo are among the key obstacles affecting the efficacy of CAR-T cell therapy (1, 6, 7, 9). Recently, there have been many efforts toward decreasing exhaustion and promoting the persistence of CAR-T cells. Combinations of CAR-T cell therapy with PD-1 blockade or knockout have been investigated in several tumors and showed promising outcomes (10, 11). However, the application of these strategies was restricted by the limited PD-1 expression in certain tumors and the systemic side effects of PD-1 blockade. In addition, targeted deletion or inhibition of genes such as *TGFBRII*, *NR4A*, and *DNMT3A* and BET proteins, Ragnase-1, and Roquin-1 has been shown to reduce T cell exhaustion, promote CAR-T cell persistence, and/or enhance antitumor activity (12–16). It has been reported that the SRC family kinase LCK, a critical kinase for T cell receptor (TCR) signaling, promotes strong signaling that tends to lead to exhaustion in CAR-T cells (17). LCK-deficient CAR-T cells show enhanced therapeutic efficacy with reduced exhaustion and enhanced memory in vivo (17). Additionally, previous studies have shown that transient treatment with dasatinib, a multi-targeted tyrosine kinase inhibitor that targets ABL, SRC, and c-KIT, reduces the expression of exhaustion markers and increases the expression of memory-associated markers through epigenetic remodeling (18–20). However, current knowledge and approaches are still quite limited in addressing these obstacles.

The Tec family nonreceptor tyrosine kinase IL-2–inducible T cell kinase (ITK), predominantly expressed in T cells, is a crucial signaling mediator downstream of TCR and regulates the strength of TCR signaling (21). ITK plays critical roles in T cell activation and differentiation (22). In *Itk*-knockout (hereafter *Itk^–/–^*) mice, T cells spontaneously develop a memory-like phenotype independent of specific antigenic stimulation (23, 24), and can rapidly produce IFN-γ upon stimulation (23, 25). In mouse models of antigen-specific CD8^+^ T cell development following infections, naive *Itk^–/–^* CD8^+^ T cells also showed significantly enhanced memory development (26, 27). Similarly, naive *Itk^–/–^* T cells expanded significantly better than WT cells under lymphogenic conditions (28). In the clinic, ibrutinib, a Bruton’s tyrosine kinase (BTK) inhibitor for B cell leukemia treatment, has been used in combination with CD19-CAR-T cells for chronic lymphocytic leukemia (CLL) therapy (29). An increased response rate of CAR-T cell therapy in CLL patients with this combination therapy has been reported (30). Because the C-terminal kinase domain has high levels of structural similarity between ITK and other Tec family members, BTK inhibitors like ibrutinib exhibit off-target effects on ITK. It has been reported that ibrutinib could directly reduce CD8^+^ T cell exhaustion independent of BTK (31). Furthermore, chronic TCR activation drives T cell exhaustion, and tempering TCR signaling by ITK inhibition or deletion will reduce T cell exhaustion in mice (32–34). However, the intrinsic role of ITK in CAR-T cells against tumors remains unaddressed.

Here, by combining the clustered regularly interspaced short palindromic repeats–associated (CRISPR-associated) protein 9 (Cas9) gene editing technology with multiple in vitro and in vivo models of CD19-CAR-T cell therapy, we showed that ITK deficiency in CD19-CAR-T cells significantly improved expansion, reduced cell exhaustion, and enhanced memory of CAR-T cells. ITK-deficient CD19-CAR-T cells showed better control of tumor relapse, leading to more sustainable therapeutic effects. In addition, ITK deficiency in CAR-T cells derived from CLL patients attenuated T cell exhaustion, a critical issue in CLL patients (35), thereby potentially addressing a challenge in CAR-T cell therapy for CLL patients. Our results suggest that deletion of *ITK* during CAR-T cell production may be a useful strategy for the development of sustainable and functional CAR-T cell therapy, which will potentially benefit patients whose T cell functional quality is low, as well as those who suffer from tumor relapses.

## Results

### ITK deficiency attenuates immediate cytotoxicity of CD19-CAR-T cells.

**Figure 1:**
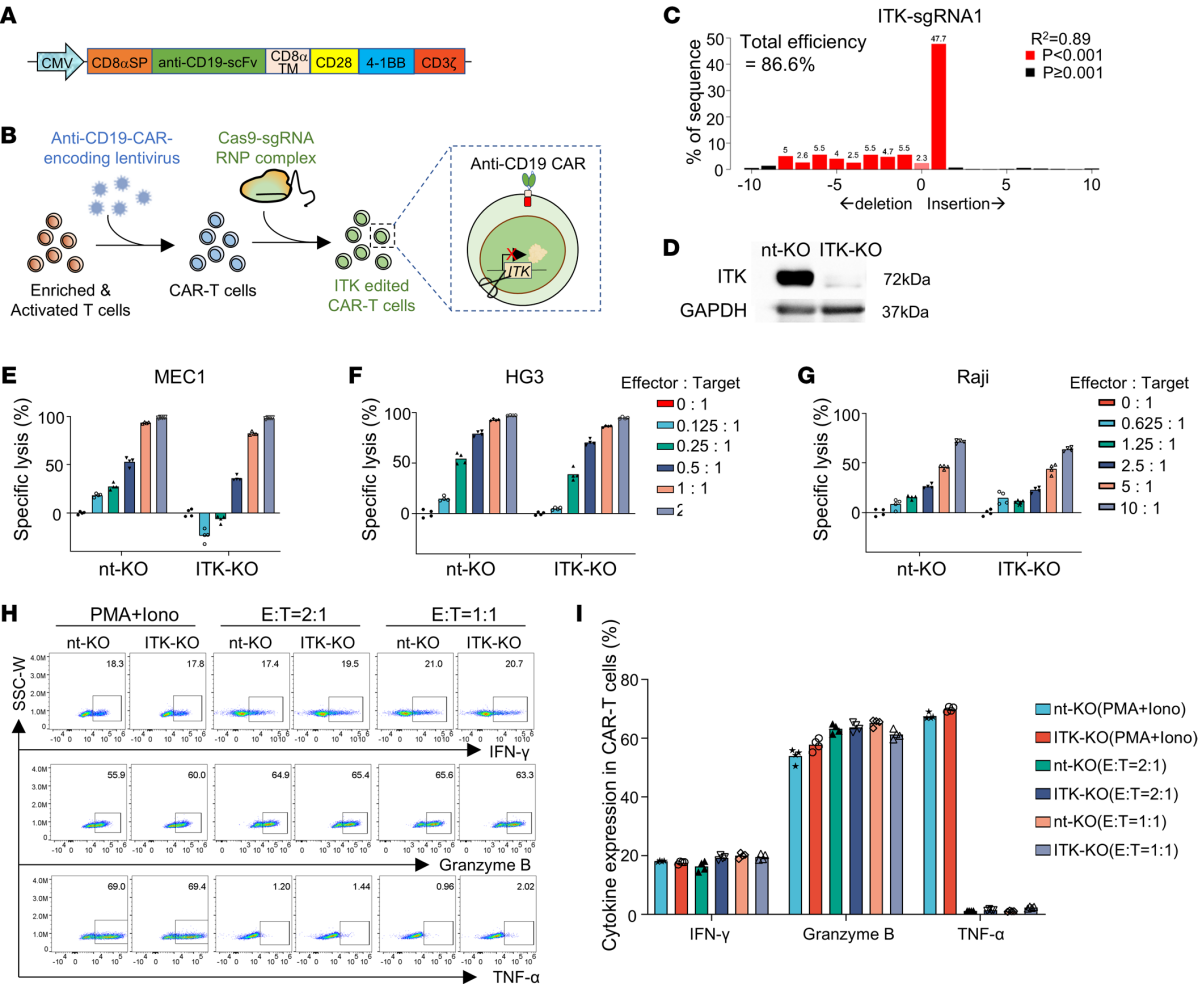
ITK deficiency attenuates immediate cytotoxicity of CAR-T cells. “nt-KO” indicates the control group of CAR-T cells electroporated with RNP complex containing nontargeting sgRNA, while “ITK-KO” indicates the group that received *ITK*-targeting sgRNA. (**A**) Schematic representation of the anti–human CD19–CAR molecule. CMV, cytomegalovirus promoter; CD8αSP, signal peptide of human CD8α; anti-CD19-scFv, single-chain fragment variable of anti–human CD19 antibody (clone: FMC63); CD8αTM, transmembrane domain of human CD8α; CD28, 4-1BB, and CD3ζ, signal transduction domains of human CD28, 4-1BB, and CD3ζ, respectively. (**B**) Generation of ITK-deficient CAR-T cells. Briefly, T cells were enriched from PBMCs and activated with anti-CD3/CD28 beads for 24 hours. Then, T cells were transduced with CAR-encoding lentivirus. Forty-eight hours after transduction, CAR-T cells were electroporated with RNP complex. (**C**) Gene editing efficiency of *ITK* locus by sgRNA1 targeting *ITK* (ITK-sgRNA1). CAR-T cells were collected for analysis 3 days after electroporation of RNP complex. (**D**) Validation of *ITK* deficiency at protein level by Western blotting. CAR-T cells were collected for Western blotting 5 days after electroporation. (**E**–**G**) In vitro killing assay against the indicated target tumor cells using control and ITK-KO CAR-T cells (*n* = 4). Luciferase-expressing MEC1, HG3, and Raji cells were mixed at the indicated ratios with CAR-T cells and analyzed 48 hours after coculture. (**H**) Representative flow cytometric plots of IFN-γ, TNF-α, and granzyme B expression in CAR-T cells stimulated as indicated. E, effector (CAR-T cells); T, target (MEC1 cells). (**I**) Summary of percentages of CAR-T cells expressing different cytokines in **H** (*n* = 4). Compiled data from 1 independent experiment for **E**–**G** and **I**. Technical replicates are shown in **E**–**G** and **I**. Data represent results of at least 2 independent experiments in **C**–**I**.

CAR-T cells targeting human CD19 (hereafter referred to as CD19-CAR-T cells) were obtained via lentiviral transduction of a third-generation CAR (Figure 1A), and *ITK* deletion in CD19-CAR-T cells was performed through electroporation of Cas9–single-guide RNA (sgRNA) ribonucleoprotein (RNP) complex (Figure 1B). Notably, we observed a modest impact on the expression of several functional molecules in T cells following lentiviral transduction and electroporation (Supplemental Figure 1, A–D; supplemental material available online with this article; https://doi.org/10.1172/JCI178558DS1). However, there was no significant impact on the nonspecific killing activity of T cells against MEC1 cells (a CD19-expressing CLL tumor cell line) or on T cell expansion following these procedures (Supplemental Figure 1, E–G). The gene editing efficiency of *ITK* was 86.6% for sgRNA1 and 96.9% for sgRNA2 (Figure 1C and Supplemental Figure 1, H and I), with sgRNA1 displaying fewer off-target effects than sgRNA2 (Supplemental Figure 1, J and K). Thus, sgRNA1 was used in the following experiments in this study unless otherwise noted. Efficient deletion of ITK (ITK-KO) was further confirmed at the protein level by Western blotting (Figure 1D). Reduced activation of TCR downstream signaling molecules, including phosphorylated PLCγ1 (p-PLCγ1), phosphorylated ERK1/2 (p-ERK1/2), and phosphorylated p70 S6 (p–p70 S6), was observed in ITK-KO CD19-CAR-T cells after coculturing with MEC1 cells (Supplemental Figure 1L). This indicates that ITK deficiency impairs TCR downstream signaling in ITK-KO CD19-CAR-T cells. Continued monitoring of CAR-T cells cocultured with MEC1 cells suggested that both ITK-KO CD19-CAR-T cells and control CD19-CAR-T cells were able to efficiently kill MEC1 cells (Supplemental Videos 1–3). The cytotoxic activity of ITK-KO CD19-CAR-T cells was further assessed in vitro against a panel of cell line models of B cell malignancy (including two CLL cell lines expressing CD19 — MEC1 and HG3 — and the Raji cell line, derived from Burkitt lymphoma). The tumor-killing ability of ITK-KO CD19-CAR-T cells was compared with that of control CD19-CAR-T cells that were electroporated with an RNP complex containing a nontargeting sgRNA (nt-KO). Deletion of *ITK* slightly reduced the cytotoxic function of CD19-CAR-T cells compared with nt-KO CD19-CAR-T cells in all tested cell lines (Figure 1, E–G). However, ITK-KO CD19-CAR-T cells showed no obvious changes in IFN-γ, TNF-α, and granzyme B expression by flow cytometric analyses, as compared with nt-KO CD19-CAR-T cells (Figure 1, H and I, and Supplemental Figure 1, M and N). Furthermore, IL-17A, FOXP3, and Th2-associated cytokines were expressed at low levels, with minimal differences observed between nt-KO and ITK-KO CD19-CAR-T cells (Supplemental Figure 1O). These results suggest that ITK deficiency in CD19-CAR-T cells attenuated the immediate cytotoxic effects against tumor cells but did not profoundly affect the production of effector cytokines.

### ITK deficiency promotes long-term expansion of CD19-CAR-T cells in vitro.

**Figure 2:**
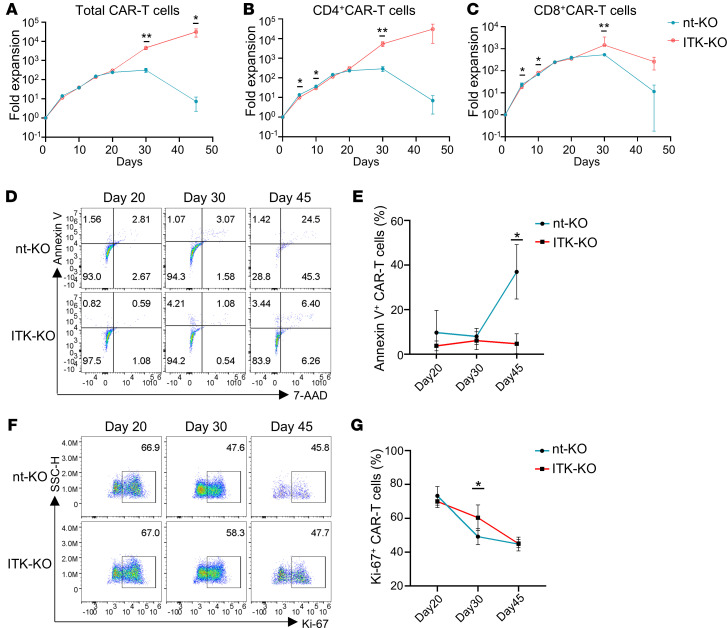
ITK deficiency promotes long-term expansion of CD19-CAR-T cells in vitro. (**A**–**C**) Fold expansion of total (**A**), CD4^+^ (**B**), and CD8^+^ (**C**) CD19-CAR-T cells at the indicated time points, following 48 hours of coculture with MEC1 cells at an E/T ratio of 2:1 (*n* = 3). Fold expansion values of the cell numbers were normalized to the average cell number of the CD19-CAR-T cells at day 0. (**D** and **E**) Representative flow cytometric plots of annexin V and 7-aminoactinomycin D (7-AAD) (**D**) and summary of percentages of annexin V^+^ cells (**E**) in CAR-T cells at the indicated time points. (**F** and **G**) Representative flow cytometric plots of Ki-67 (**F**) and summary of percentages of Ki-67^+^ cells (**G**) in CAR-T cells at the indicated time points. *n* = 3 for each group in **E** and **G**. Compiled data from 1 independent experiment in **A**–**C**, **E**, and **G**. Data represent results of at least 2 independent experiments. Statistical differences were determined by 2-tailed unpaired Student’s *t* test. *P < 0.05, **P < 0.01.

To further determine the role of ITK in CD19-CAR-T cells, we examined cell expansion, proliferation, and apoptosis in ITK-KO CD19-CAR-T cells and nt-KO CD19-CAR-T cells after coculture with tumor cells. ITK-KO CD19-CAR-T cells exhibited similar rates of cell expansion at the early time points (e.g., before day 20 after coculture) compared with nt-KO CD19-CAR-T cells (Figure 2, A–C). However, we observed significant improvements in the expansion of ITK-KO CD19-CAR-T cells at later time points compared with nt-KO CD19-CAR-T cells (both CD4^+^ and CD8^+^ CD19-CAR-T cells) (Figure 2, A–C, and Supplemental Figure 2). Annexin V staining revealed that apoptosis of nt-KO CAR-T cells was significantly increased 45 days after in vitro culture. In contrast, ITK-KO CD19-CAR-T cells displayed a significantly lower level of apoptosis at this time point (Figure 2, D and E). Furthermore, Ki-67 staining demonstrated that the proliferation of both nt-KO and ITK-KO CD19-CAR-T cells decreased over time, but this reduction was smaller in ITK-KO CD19-CAR-T cells compared with nt-KO CAR-T cells at day 30 (Figure 2, F and G). Overall, these data indicate that ITK deficiency enhances long-term expansion with significantly better survival and slower reduction of proliferation of CD19-CAR-T cells in vitro.

### Transcriptomic regulation by ITK in CD19-CAR-T cells.

**Figure 3:**
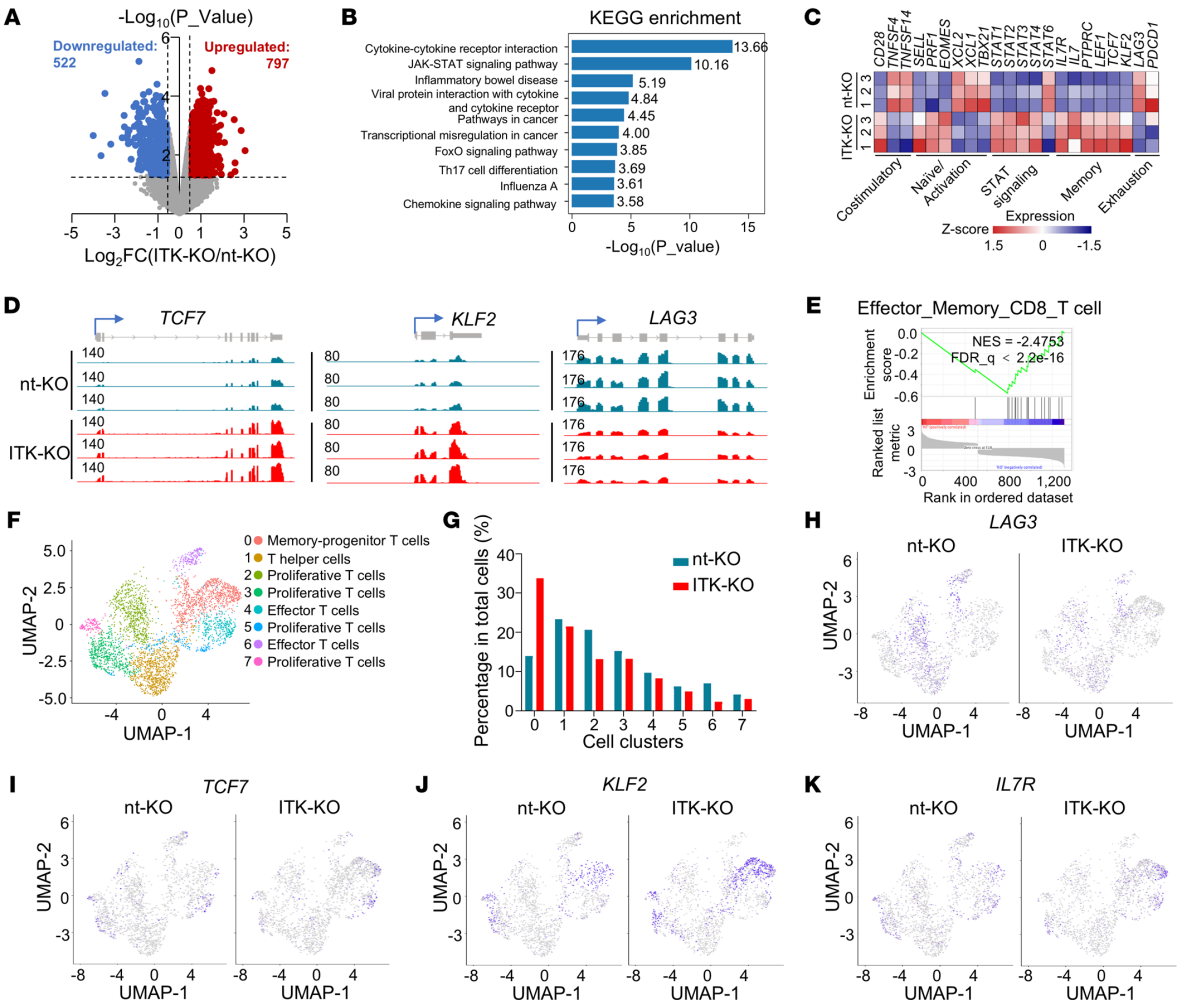
Transcriptomic regulation of CD19-CAR-T cells by ITK. CAR-T cells were cocultured with MEC1 cells for 48 hours and sort-purified for both bulk and single-cell RNA sequencing (RNA-Seq). (**A**–**E**) Bulk RNA-Seq analysis of the transcriptome in nt-KO and ITK-KO CAR-T cells. (**A**) Differential gene expression (transcripts per million ≥ 1, *P* ≤ 0.05, fold change ≥ 1.5). Unadjusted *P* values are shown. (**B**) KEGG analysis of the differentially expressed genes. (**C**) Heatmap of the indicated gene expression. (**D**) RNA-Seq tracks of read coverage at the *TCF7* (left), *KLF2* (middle), and *LAG3* (right) loci in CAR-T cells. (**E**) Gene set enrichment analysis of the effector-memory CD8^+^ T cell gene set analysis using differentially expressed genes shown in **A**. (**F**–**K**) Single-cell RNA-Seq (scRNA-Seq) analysis of the transcriptome in nt-KO and ITK-KO CAR-T cells. (**F**) Clustering of functional T cell subsets of all CAR-T cells based on uniform manifold approximation and projection (UMAP). (**G**) Percentage of indicated T cell subsets within total T cells identified by scRNA-Seq in **F**. (**H**–**K**) Expression of indicated genes in nt-KO and ITK-KO CD19-CAR-T cells.

To investigate how ITK regulates the transcriptome of CAR-T cells, we first conducted high-throughput RNA sequencing (bulk RNA-Seq) on ITK-KO and nt-KO CD19-CAR-T cells. A total of 1,319 genes were significantly differentially expressed between nt-KO and ITK-KO CD19-CAR-T cells (fold change ≥ 1.5, *P* ≤ 0.05) (Figure 3A and Supplemental Figure 3, A and B). Among these, 797 were increased while 522 were decreased in the ITK-KO CD19-CAR-T cells (Figure 3A and Supplemental Figure 3B). In line with the critical role of ITK in TCR signaling, we observed a significant enrichment of differentially expressed genes involved in several immune signaling pathways downstream of the TCR signaling, such as cytokine–cytokine receptor interaction and cytokine-mediated signaling pathways (Figure 3B and Supplemental Figure 3C), by Kyoto Encyclopedia of Genes and Genomes (KEGG) and Gene Ontology (GO) analyses. In addition, multiple genes involved in T cell activation, such as *TNFSF4*, *TNFSF14*, *XCL1*, *XCL2* and *TBX21*, were significantly decreased in the ITK-KO CD19-CAR-T cells (Figure 3C). Furthermore, transcription of several key JAK/STAT signaling genes, such as *STAT1*, *STAT2*, *STAT3*, and *STAT4*, but not *STAT6*, was significantly increased in ITK-KO CD19-CAR-T cells (Figure 3C). Enhancement of STAT1, STAT3, and STAT5, but not STAT6, had been shown to promote stem cell memory and effector CAR-T cells (36). Our observations suggested that ITK deficiency regulated TCR signaling in CD19-CAR-T cells and may promote effector-memory formation in CAR-T cells. Indeed, several genes associated with naive or progenitor-memory T cells, including *SELL* (encoding CD62L), *IL7R* (encoding CD127), *TCF7* (encoding TCF1), *KLF2*, and *LEF1*, were significantly increased in the ITK-KO CD19-CAR-T cells (Figure 3, C and D). Gene set enrichment analysis further indicated significant enrichment of genes associated with T cell effector-memory phenotype in the ITK-KO CD19-CAR-T cells (Figure 3E). In contrast, genes involved in T cell exhaustion (37), such as *PDCD1* (encoding PD-1) and *LAG3*, were significantly decreased in ITK-KO CD19-CAR-T cells (Figure 3C). Together, our results suggested that ITK may affect CAR-T cell activation, decrease T cell exhaustion, and promote memory formation.

To gain further insights into how ITK regulates the heterogeneity and transcriptomic profile of CAR-T cells, we conducted single-cell RNA sequencing (scRNA-Seq) on ITK-KO and nt-KO CD19-CAR-T cells, following stimulation by MEC1 cells for 48 hours. By merging both nt-KO and ITK-KO CD19-CAR-T cells, we identified 8 T cell populations with different states (Figure 3, F and G, and Supplemental Figure 3, D–F). Interestingly, while other populations were at a similar level between nt-KO and ITK-KO CD19-CAR-T cells, we observed that the memory-progenitor population in ITK-KO CD19-CAR-T cells was almost 2-fold higher than that in nt-KO CD19-CAR-T cells (33.74% in ITK-KO CD19-CAR-T cells vs. 13.94% in nt-KO CD19-CAR-T cells) (Figure 3, F and G, and Supplemental Figure 3, D–F). In line with the observations from bulk RNA-Seq, lower expression of *LAG3* was observed in ITK-KO CD19-CAR-T cells (Figure 3H). In contrast, multiple genes highly expressed in memory T cells, such as *TCF7* (encoding TCF1), *KLF2*, and *IL7R* (encoding CD127), showed increased expression in ITK-KO CD19-CAR-T cells, especially within the memory-progenitor T cell population (Figure 3, I–K). This is consistent with the observed increase of *TCF7*, *KLF2*, and *IL7R* expression in ITK-KO CD19-CAR-T cells in the bulk RNA-Seq analysis (Figure 3, C and D, and Supplemental Figure 3G). These findings collectively indicate that ITK deficiency may reduce exhaustion and enhance T cell memory fate in CD19-CAR-T cells.

### ITK deficiency reduces exhaustion and promotes memory phenotype in CD19-CAR-T cells in vitro.

**Figure 4:**
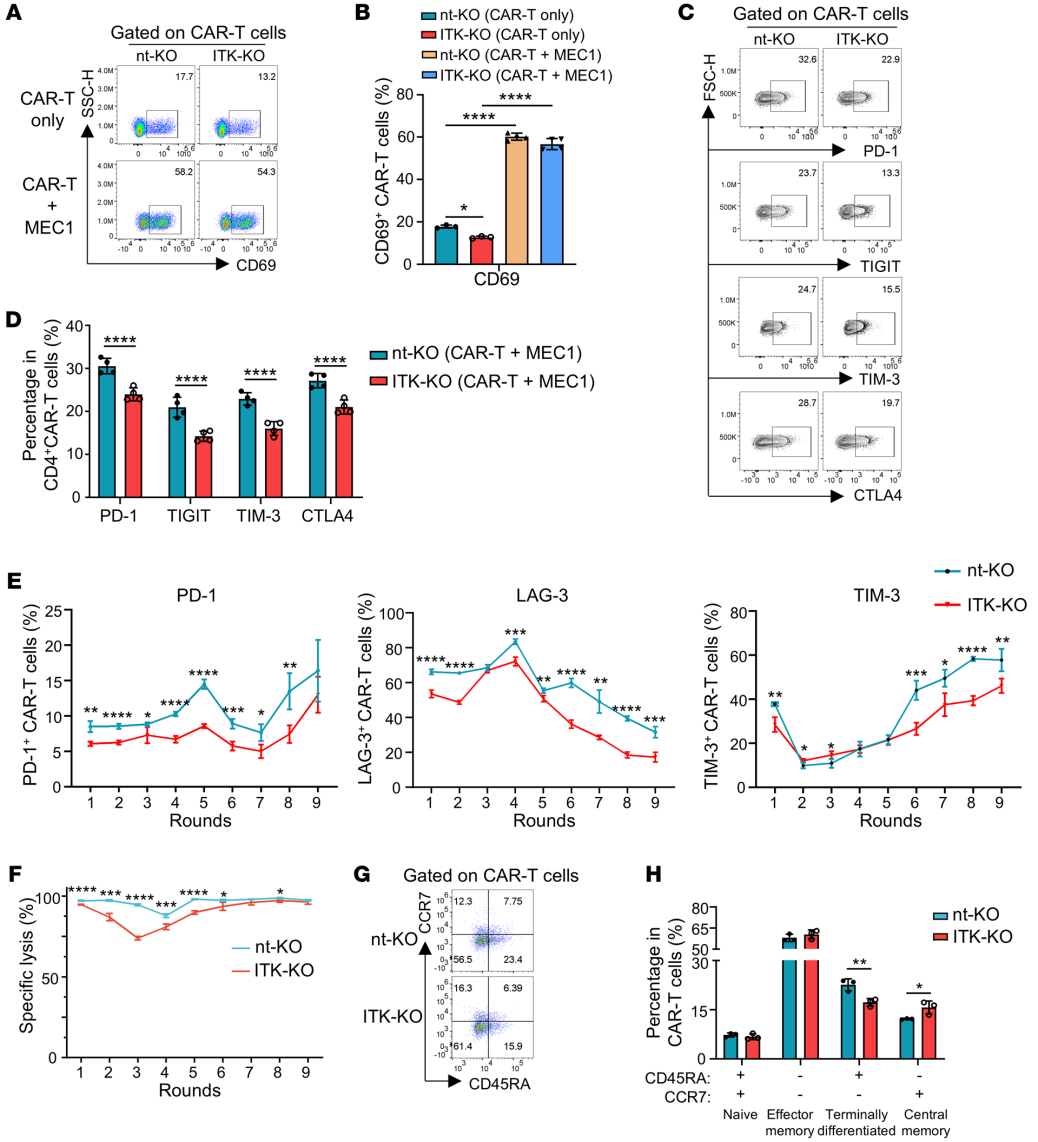
ITK deficiency reduces exhaustion and promotes memory phenotype in CD19-CAR-T cells in vitro. CAR-T cells were cocultured with MEC1 after the serial killing assay protocol detailed in Methods. (**A**) Representative flow cytometric plots of CD69 expression in the indicated CAR-T cells in the presence or absence of MEC1 cells. CAR-T cells were analyzed after coculture with MEC1 cells (E/T = 2:1) for 48 hours. (**B**) Summary of percentages of CD69^+^ cells in **A** (*n* = 3 for each CAR-T-only group, and *n* = 4 for each CAR-T + MEC1 group) (2-way ANOVA with Šidák’s correction for multiple comparisons). (**C**) Representative flow cytometric plots of PD-1, TIGIT, TIM-3, and CTLA4 expression in CAR-T cells 15 days after coculture with MEC1 cells at the ratio of E/T = 2:1. (**D**) Summary of percentages of PD-1^+^, TIGTI^+^, TIM-3^+^, and CTLA4^+^ cells as shown in **C** (*n* = 4). (**E**) Summary of percentages of LAG-3^+^, PD-1^+^, and TIM-3^+^ cells in the indicated CAR-T cells following the indicated rounds of coculture with MEC1 cells (*n* = 4). CAR-T cells were cocultured with MEC1 cells at a 2:1 ratio for 48 hours for each round for **E** and **F**. (**F**) Percentages of specific lysis determined by in vitro killing assay of MEC1 cells by the indicated CAR-T cells at indicated rounds of coculture (*n* = 4). (**G**) Representative flow cytometric plots of CD45RA and CCR7 expression in the indicated CAR-T cells 15 days after coculture with MEC1 cells (E/T = 2:1). (**H**) Summary of percentages of CAR-T cells expressing CD45RA and/or CCR7 in **G** (*n* = 3). Compiled data from 1 independent experiment for **B**, **D**–**F**, and **H**. Statistical differences were determined by 2-tailed unpaired Student’s *t* test in **D**–**F** and **H**. Data represent results of at least 2 independent experiments. *P < 0.05, **P < 0.01, ***P < 0.001, ****P < 0.0001.

To further investigate the role of ITK in regulating T cell activation, exhaustion, and memory, we determined the protein expression of multiple key molecules involved in these processes between nt-KO and ITK-KO CD19-CAR-T cells under steady state or cocultured with tumor cells. Despite a slight decrease in CD69 expression in ITK-KO CD19-CAR-T cells under steady-state conditions (Figure 4, A and B), we observed a significant upregulation of CD69 expression in the ITK-KO CD19-CAR-T cells upon stimulation with MEC1 cells, reaching levels comparable to those observed in nt-KO CD19-CAR-T cells (Figure 4, A and B). These results indicated that ITK deficiency did not affect the activation of CD19-CAR-T cells following stimulation by the targeted tumor cells. Interestingly, we noted a significant downregulation of the expression of multiple coinhibitory molecules associated with CAR-T cell exhaustion (37), including LAG-3, PD-1, and TIM-3, in ITK-KO CD19-CAR-T cells compared with nt-KO CD19-CAR-T cells 48 hours after coculture with tumor cells (Supplemental Figure 4, A–D). This downregulation was observable in ITK-KO CD19-CAR-T cells both at the steady state and after coculture with MEC1 cells or Raji cells (Supplemental Figure 4, A–F). Moreover, downregulation of coinhibitory molecules, including PD-1, TIGIT, TIM-3, and CTLA4, was observed in ITK-KO CD19-CAR-T cells 15 days after coculture with MEC1 cells (Figure 4, C and D, and Supplemental Figure 4, G and H). Remarkably, the decrease in expression of PD-1, LAG-3, and TIM-3 in ITK-KO CD19-CAR-T cells, compared with nt-KO CD19-CAR-T cells, remained significant after multiple rounds of exposure to the targeted tumor cells (Figure 4E). We also analyzed the cytotoxic potential of ITK-KO CD19-CAR-T cells following repeated exposure to cancer cells. While we observed a limited but significant decrease in their cytotoxic effect in the initial phases of antigen exposure (e.g., rounds 1 through 6 and round 8), the tumor-killing ability of ITK-KO CD19-CAR-T cells reached levels comparable to those of nt-KO CD19-CAR-T cells at later time points (e.g., rounds 7 and 9) (Figure 4F and Supplemental Figure 4I). These data suggest that ITK-KO CD19-CAR-T cells retain good antitumor activity following repeated exposure to CD19^+^ cancer cells, probably due to less T cell exhaustion and enhanced memory development. In fact, following exposure to tumor cells, there was a significantly decreased fraction of terminally differentiated cells and an increased fraction of central memory cells in ITK-KO CD19-CAR-T cells compared with nt-KO CD19-CAR-T cells (Figure 4, G and H, and Supplemental Figure 4, J and K). Together, these results suggest that ITK deficiency decreases T cell exhaustion and enhances memory fate of CD19-CAR-T cells in vitro.

### ITK deficiency enhances expansion and long-term persistence of CD19-CAR-T cells in vivo.

**Figure 5:**
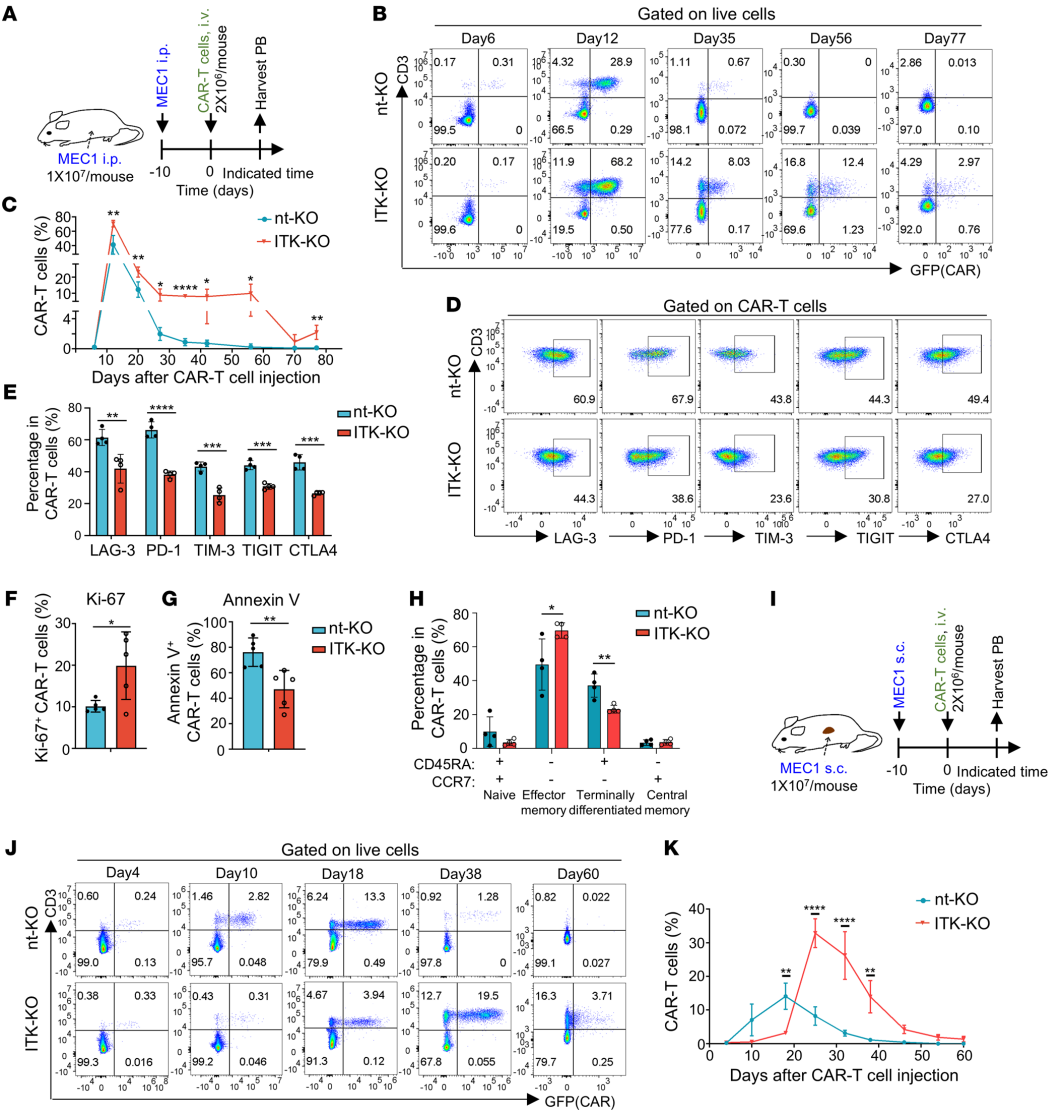
*ITK* deficiency enhances expansion and long-term persistence of CD19-CAR-T cells in vivo. (**A**) Experimental design of CAR-T cell therapy against intraperitoneally injected MEC1 cells in NPG mice. CAR-T cells were expanded for 11 days after electroporation before intravenous injection into mice. PB, peripheral blood. (**B**) Representative flow cytometric plots of CAR-GFP and CD3 in PB samples collected from nt-KO or ITK-KO CD19-CAR-T cell recipients at the indicated time points. (**C**) Summary of percentages of CAR-T cells (CD3^+^GFP^+^) shown in **B** (*n* = 3 for day 77 ITK-KO group and *n* = 4 for the rest). (**D**) Representative flow cytometric plots of expression of the indicated molecules by CAR-T cells in PB samples collected from nt-KO or ITK-KO-CAR T cell recipients 28 days after CAR-T cell infusion. (**E**) Summary of percentages of CAR-T cells that are LAG-3^+^, PD-1^+^, TIM-3^+^, TIGIT^+^, or CTLA4^+^, as shown in **D** (*n* = 4). (**F** and **G**) Summary of percentages of CAR-T cells that are Ki-67^+^ (**F**) and annexin V^+^ (**G**) as shown in Supplemental Figure 5, C and D (*n* = 5). (**H**) Statistical analysis of different populations of cells shown in Supplemental Figure 5E (*n* = 4). (**I**) Experimental design of CAR-T cell therapy against subcutaneously injected MEC1 cells in NPG mice. CAR-T cells were expanded for 11 days after electroporation before intravenous injection into mice. (**J**) Representative flow cytometric plots of CAR-GFP and CD3 in PB samples collected from nt-KO or ITK-KO CD19-CAR recipients at the indicated time points. (**K**) Summary of percentages of CAR-T cells (CD3^+^GFP^+^) shown in **J** (*n* = 4, mean ± SEM). Compiled data from 1 independent experiment for **C**, **E**–**H**, and **K**. Statistical differences were determined by 2-tailed unpaired Student’s *t* test. Data represent results of at least 2 independent experiments. *P < 0.05, **P < 0.01, ***P < 0.001, ****P < 0.0001.

To test the long-term effects of *ITK* deletion on CD19-CAR-T cells in vivo, we first used a CLL mouse model of NOD-*Prkdc^scid^Il2rγ^null^* (NPG) mice bearing MEC1 tumor cells (injected intraperitoneally) followed by infusion with ITK-KO or nt-KO CD19-CAR-T cells (Figure 5A). In this tumor model, both ITK-KO and nt-KO CD19-CAR-T cells efficiently cleared tumor cells 14 days after infusion (Supplemental Figure 5A). There was no significant difference in the body weight of mice between ITK-KO and nt-KO CD19-CAR-T cell–injected groups (Supplemental Figure 5B). Interestingly, upon tumor clearance, the abundance of ITK-KO CD19-CAR-T cells peaked at a significantly higher level than that of nt-KO CD19-CAR-T cells (Figure 5, B and C), in agreement with the enhanced expansion of ITK-KO CD19-CAR-T cells. In addition, while nt-KO CD19-CAR-T cells rapidly contracted to levels that were nearly undetectable, ITK-KO CD19-CAR-T cells remained in circulation at a significantly higher level even 77 days after infusion (Figure 5, B and C). Moreover, ITK-KO CD19-CAR-T cells showed significantly lower expression of multiple coinhibitory molecules that are associated with T cell exhaustion, including LAG-3, PD-1, TIM-3, TIGIT, and CTLA4 (Figure 5, D and E). As expected, significant decrease of apoptosis (indicated by annexin V) and significant increase of proliferation (indicated by Ki-67) were observed in ITK-KO CD19-CAR-T cells compared with nt-KO CD19-CAR-T cells (Figure 5, F and G, and Supplemental Figure 5, C and D). Notably, we observed a high level of apoptosis in CAR-T cells (Supplemental Figure 5D), which may be because these apoptotic CAR-T cells were in the contraction phase at this time point and were not rapidly cleared in the immune-deficient recipients. Remarkably, there were more effector-memory and less terminally differentiated CAR-T cells in mice injected with ITK-KO CD19-CAR-T cells compared with controls (Figure 5H and Supplemental Figure 5E). As enhanced memory cell phenotype has been correlated with improved long-term CAR-T cell therapeutic effects (36, 38, 39), our results suggest that ITK deficiency enhances expansion and long-term persistence of CD19-CAR-T cells as a result of ITK-mediated reduction of exhaustion and improvement of memory in CAR-T cells in vivo.

The CLL cell line MEC1 could also be subcutaneously injected into NPG mice and form a solid tumor (40). We further tested the impact of *ITK* deletion on CD19-CAR-T cells in this mouse model of CLL with solid tumor (41) (Figure 5I). Consistent with the previous intraperitoneal MEC1 injection model, ITK-deficient CD19-CAR-T cells not only expanded to a higher peak level but also persisted much longer in the peripheral blood after injection into the MEC1-bearing NPG mice (Figure 5, J and K). Interestingly, we found that ITK-deficient CD19-CAR-T cells showed a slight delay in the pattern of increase compared with nt-KO CD19-CAR-T cells (Figure 5, J and K). This observation might be consistent with the results that mice from nt-KO CD19-CAR-T cell–injected groups showed a trend of faster tumor cell clearance, although with no significant difference compared with mice from ITK-KO CD19-CAR-T cell–injected groups (Supplemental Figure 5F). The body weight of mice between ITK-KO and nt-KO CD19-CAR-T cell–injected groups also showed no significant difference in this tumor model (Supplemental Figure 5G).

Overall, these results show that ITK deficiency enables enhanced expansion and long-term persistence of CD19-CAR-T cells in vivo in preclinical animal models of CLL.

### ITK-deficient CAR-T cells significantly improve control of tumor relapse in vivo.

**Figure 6:**
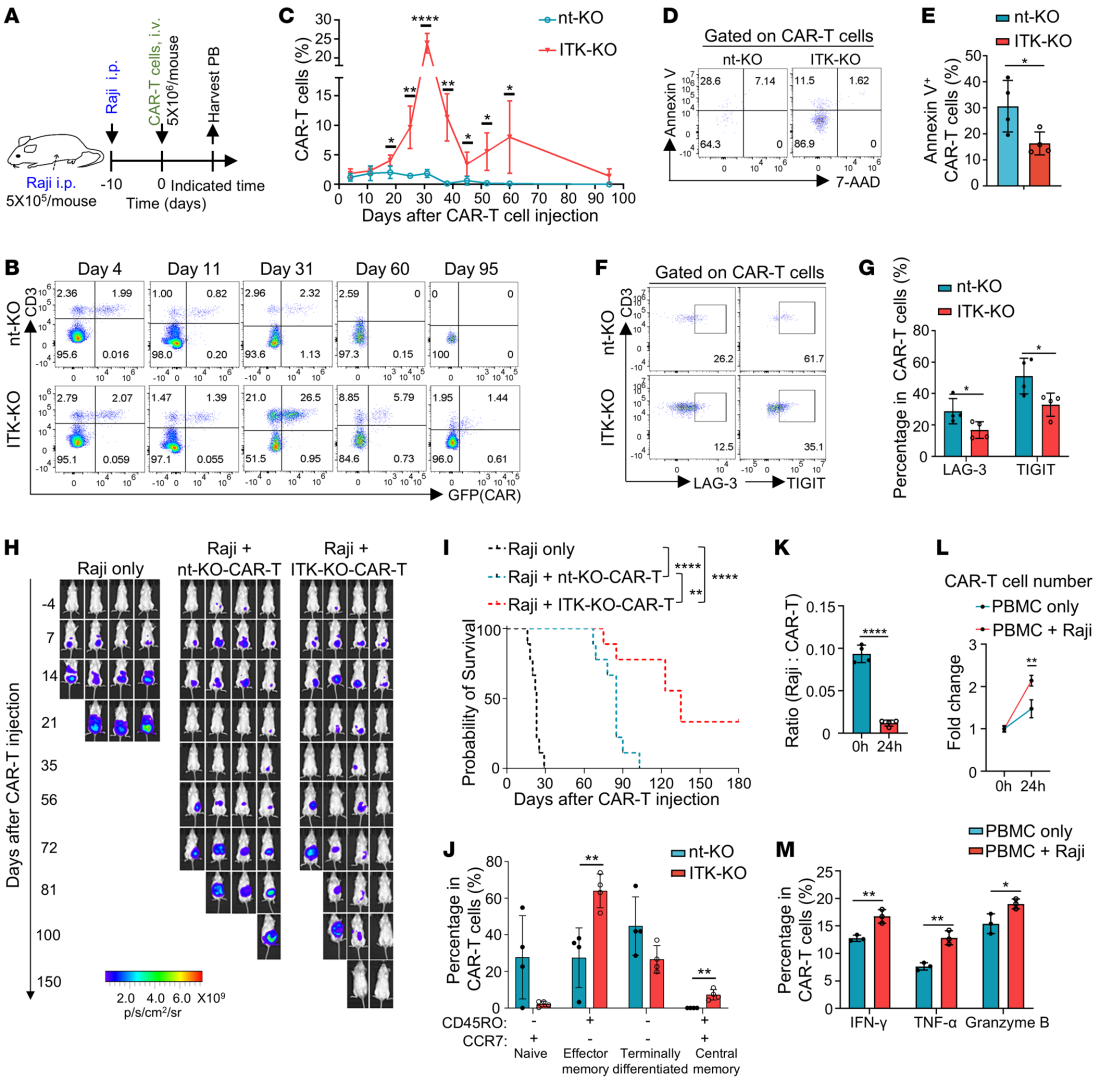
ITK*-*deficient CAR-T cells enhance control of tumor relapse in vivo. (**A**) Experimental design of CAR-T cell therapy against intraperitoneally injected Raji cells in NPG mice. CAR-T cells were expanded for 11 days after electroporation. (**B**) Flow cytometric plots of CAR-GFP and CD3 in PB samples collected from indicated recipients at the indicated time points. (**C**) Summary of CAR-T cell percentages (CD3^+^GFP^+^) in **B** (*n* = 1 for day 95 nt-KO group, *n* = 3 for day 95 ITK-KO group, *n* = 4 for the rest). (**D**) Representative flow cytometric plots of annexin V and 7-AAD expression by CAR-T cells from PB samples 28 days after CAR-T cell injection. (**E**) Summary of annexin V^+^ CAR-T cells percentages in **D** (*n* = 4). (**F**) Flow cytometric plots of LAG-3 and TIGIT expression by CAR-T cells from PB samples 24 days after CAR-T cell infusion. (**G**) Summary of LAG-3^+^ or TIGIT^+^ CAR-T cell pecentages in **F** (*n* = 4). (**H**) Bioluminescence images of NPG mice xenografted with Raji cells as in **A**. Representative figures from 1 independent experiment. (**I**) Kaplan-Meier survival of Raji-bearing NPG mice (*n* = 9) (log-rank Mantel-Cox test with Bonferroni’s correction for multiple comparisons). Compiled data from 2 independent experiments. (**J**) Statistical analysis of CD45RO and/or CCR7 expression by CAR-T cells in Supplemental Figure 6G (*n* = 4). (**K**, **L**) Statistical analysis of Raji to CAR-T cells ratios (**K**) and CAR-T cell number fold changes (**L**) as shown in Supplemental Figure 6H (n = 4). (**M**) Statistical analysis of IFN-γ, TNF-α, and granzyme B expression in CAR-T cells shown in Supplemental Figure 6I (*n* = 3). Compiled data from 1 independent experiment for **C**, **E**, **G**, and **J**–**M**. Two-tailed unpaired Student’s *t* test was performed in **C**, **E**, **G**, and **J**–**M**. Data represent at least 2 independent experiments. *P < 0.05, **P < 0.01, ****P < 0.0001.

As we observed significantly improved expansion and long-term persistence of ITK-deficient CD19-CAR-T cells compared with nt-KO CD19-CAR-T cells in vivo, we speculated that mice receiving ITK-KO CD19-CAR-T cells might be better protected against tumor relapse. The Raji cell xenograft mouse model is known to relapse after the first wave of tumor clearance by CAR-T cells (42, 43). Therefore, we used this model (Figure 6A) to assess the function of ITK in CD19-CAR-T cell activity against tumor relapse. In this model, ITK-KO CD19-CAR-T cells showed significantly enhanced expansion and long-term survival compared with nt-KO CD19-CAR-T cells (Figure 6, B and C), as ITK-KO CD19-CAR-T cells displayed lower levels of apoptotic cells and increased proliferation (Figure 6, D and E, and Supplemental Figure 6, A and B). In addition, ITK-KO CD19-CAR-T cells showed significantly reduced levels of the T cell exhaustion molecules LAG-3 and TIGIT (Figure 6, F and G, and Supplemental Figure 6, C and D). Raji cell–bearing mice that were treated without CAR-T cells showed progression of tumor growth and succumbed to tumor growth before 40 days after Raji cell injection (Figure 6, H and I). In contrast, tumor-grafted mice that received nt-KO CD19-CAR-T cells cleared tumors within 21 days but showed notable cancer relapse approximately 56 days after CAR-T cell injection (Figure 6, H and I). This group of mice also finally died of uncontrolled tumor relapse (Figure 6, H and I). Interestingly, even after tumor relapse at day 56, animals that received ITK-KO CD19-CAR-T cells exhibited better control of the relapsed tumor, leading to significantly improved survival (Figure 6, H and I, and Supplemental Figure 6E). No significant difference in body weight was observed between groups of mice that received either nt-KO or ITK-KO CD19-CAR-T cells (Supplemental Figure 6F). These results demonstrate that ITK deficiency in CD19-CAR-T cells promotes CAR-T cell expansion and survival in vivo and enables better control of tumor relapse.

Higher frequency of effector-memory and central memory CAR-T cell populations was observed in mice that received ITK-KO CD19-CAR-T compared with mice that received nt-KO CD19-CAR-T cells (Figure 6J and Supplemental Figure 6G). To further validate the long-term effector-memory function of ITK-KO CD19-CAR-T cells in vivo, we tested the memory-recall responses of ex vivo ITK-KO CD19-CAR-T cells from Raji-grafted mice at day 50 after CAR-T cell injection (when the first wave of Raji growth had been controlled). Ex vivo ITK-KO CD19-CAR-T cells showed significant control of Raji cell growth and expansion when cocultured with Raji cells (Figure 6, K–M). Furthermore, we observed an increase of IFN-γ, TNF-α, and granzyme B expression in ex vivo ITK-KO CD19-CAR-T cells after coculture with Raji cells (Figure 6M and Supplemental Figure 6H). Together, our results suggest that ITK deficiency in CD19-CAR-T cells promotes CD19-CAR-T cell memory fate associated with less T cell exhaustion and better in vivo persistence, providing a potentially more sustainable CAR-T cell therapy.

### ITK deficiency attenuates exhaustion and promotes memory phenotype in CD19-CAR-T cells derived from CLL patients.

**Figure 7:**
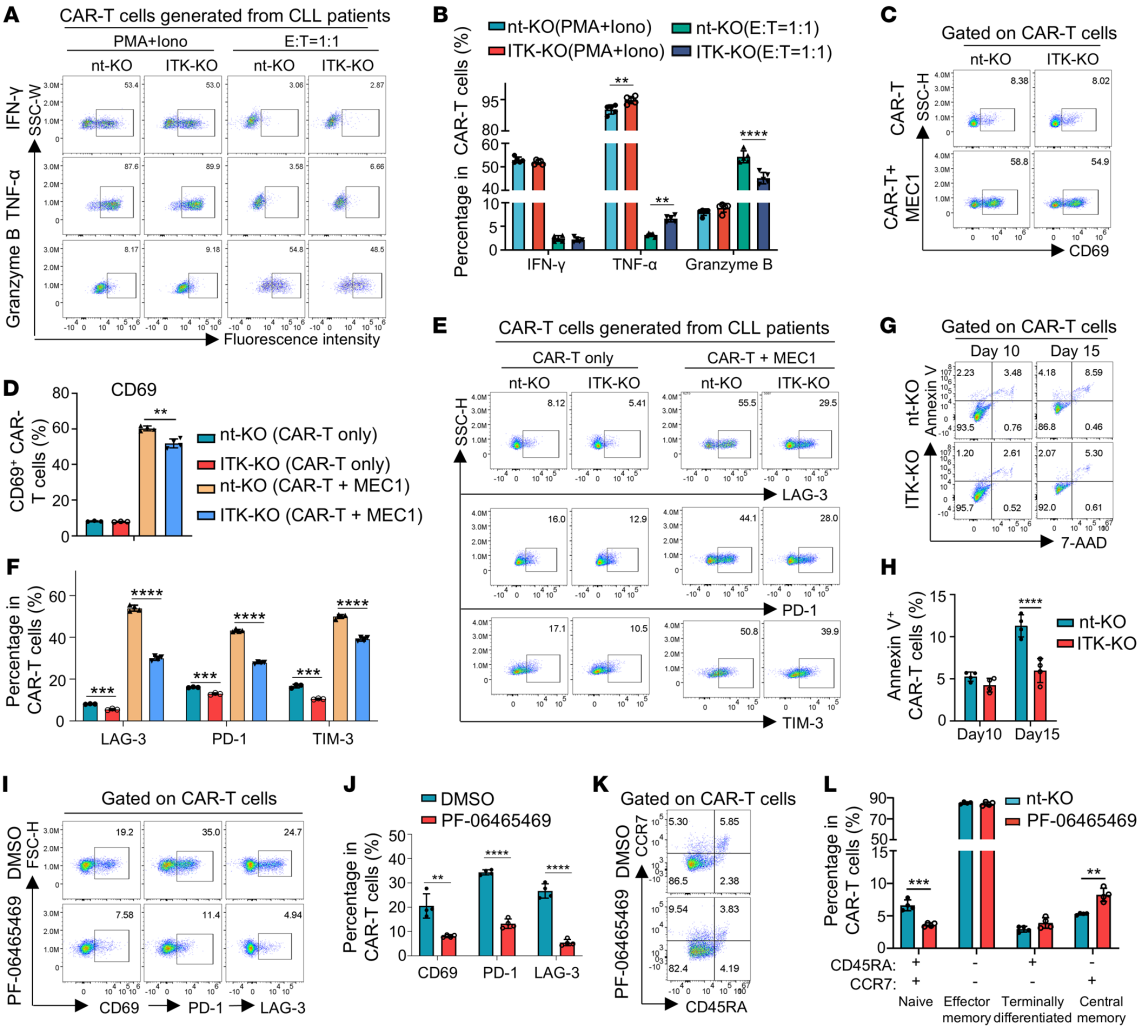
ITK deficiency attenuates exhaustion and promotes memory phenotype in CD19-CAR-T cells derived from CLL patients. (**A**) Flow cytometric analyses of IFN-γ, TNF-α, and granzyme B expression in CLL-CAR-T cells. CLL-CAR-T cells were cocultured with MEC1 cells at a 2:1 ratio for 48 hours in **A**–**J**. (**B**) Statistical analysis of percentage of IFN-γ, TNF-α, and granzyme B expression in CAR-T cells shown in **A** (*n* = 5). (**C**) Flow cytometry analyses of CD69 expression in indicated CLL-CAR-T cells after coculture with or without MEC1 cells. (**D**) Statistical analysis of CD69 expression shown in **C** (*n* = 3 for CAR-T-only group, *n* = 4 for CAR-T + MEC1 group). (**E**) Flow cytometry analyses of LAG-3, PD-1, and TIM-3 expression in indicated CLL-CAR-T cells. (**F**) Statistical analysis of percentage of LAG-3^+^, PD-1^+^, and TIM-3^+^ cells shown in **E** (*n* = 3 for CAR-T-only group, *n* = 4 for CAR-T + MEC1 group). (**G**) Flow cytometry analyses of annexin V and 7-AAD expression in indicated CLL-CAR-T cells at indicated time points. (**H**) Statistical analysis of annexin V^+^ CAR-T cells shown in **G** (*n* = 4). (**I**) Flow cytometry analyses of CD69, LAG-3, and PD-1 expression in indicated CAR-T cells cocultured with MEC1 cells with or without PF-06465469 (1 μM) treatment. (**J**) Statistical analysis of percentage of CD69^+^, LAG-3^+^, and PD-1^+^ cells in CD19-CAR-T cells shown in **I** (*n* = 4). (**K**) Representative flow cytometric plots of CD45RA and CCR7 expression in indicated CAR-T cells 15 days after PF-06465469 treatment. (**L**) Summary of percentages of CAR-T cells expressing CD45RA and/or CCR7 in **K** (*n* = 4). Compiled data from 1 independent experiment for **B**, **D**, **F**, **H**, **J**, and **L**. Statistical differences were determined by 2-tailed unpaired Student’s *t* test. Data represent results of at least 2 independent experiments. **P < 0.01, ***P < 0.001, ****P < 0.0001.

T cells from CLL patients show notable exhaustion (35), and prior treatment with ibrutinib, a BTK inhibitor, was associated with an increased response rate of CAR-T cell therapy in CLL patients (30). Since ibrutinib can also inhibit ITK (44), we speculate that the improved CAR-T cell therapeutic effects in ibrutinib-treated CLL patients are in part due to the inhibition of ITK in CAR-T cells. Therefore, we derived CD19-CAR-T cells from PBMCs of CLL patients (referred to as CLL-CAR-T cells) and investigated the role of ITK in CLL-CAR-T cells. Notably, while the total cell number of PBMCs from CLL patients was comparable to that from healthy donors (Supplemental Figure 7A), the proportion of T cells was significantly reduced in PBMCs from CLL patients (Supplemental Figure 7, B–E). The viability of T cells from CLL patients was similar to that of T cells from healthy donors (Supplemental Figure 7F). ITK-KO CLL-CAR-T cells showed slightly increased TNF-α expression following stimulation (Figure 7, A and B). In addition, a slight decrease in T cell activation as indicated by CD69 was observed in ITK-KO CLL-CAR-T cells when cocultured with MEC1 cells (Figure 7, C and D). And as expected, ITK-KO CLL-CAR-T cells showed a significant decrease in the expression of coinhibitory molecules, including LAG-3, PD-1, and TIM-3 (Figure 7, E and F). Furthermore, ITK-KO CLL-CAR-T cells exhibited significantly lower apoptosis when cocultured with MEC1 cells for 15 days compared with nt-KO CLL-CAR-T cells (Figure 7, G and H). ITK-KO CLL-CAR-T cells also demonstrated enhanced expansion in vitro compared with nt-KO CLL-CAR-T cells, particularly at later time points (Supplemental Figure 7G), despite having attenuated cytotoxic function, especially at low effector/target ratio (Supplemental Figure 7H).

To further determine whether inhibiting ITK activity during the production of CAR-T cells could mimic *ITK* deletion, we investigated the effects of PF-06465469 (a potent ITK inhibitor that also inhibits BTK) and ibrutinib on CLL-CAR-T cells. Both PF-06465469 and ibrutinib significantly decreased PD-1 and LAG-3 expression (Figure 7, I and J, and Supplemental Figure 7, I–N), suggesting a potential decrease of exhaustion in CLL-CAR-T cells. Notably, CD69 expression was also significantly decreased after PF-06465469 and ibrutinib treatment (Figure 7, I and J, and Supplemental Figure 7, M and N), indicating reduced T cell activation. Consistent with the expectation from previous results, there was a significantly increased fraction of central memory cells in CLL-CAR-T cells 15 days after PF-06465469 treatment (Figure 7, K and L), suggesting that ITK inhibition could also promote T cell memory in CD19-CAR-T cells generated from CLL patients.

Notably, infusion of the inhibitor-treated CLL-CAR-T cells or control DMSO-treated CLL-CAR-T cells into MEC1-bearing mice revealed only a trend but no significant increase of CLL-CAR-T cells (Supplemental Figure 7, O–R). These data suggest that while inhibiting ITK during CAR-T cell production can temporarily alleviate exhaustion and promote memory phenotype in CLL-CAR-T cells in vitro, genetic targeting or continuous pharmacological inhibition of T cell–intrinsic ITK signaling is probably required to sustain the long-term responses of CAR-T cells against tumors.

**Figure 8:**
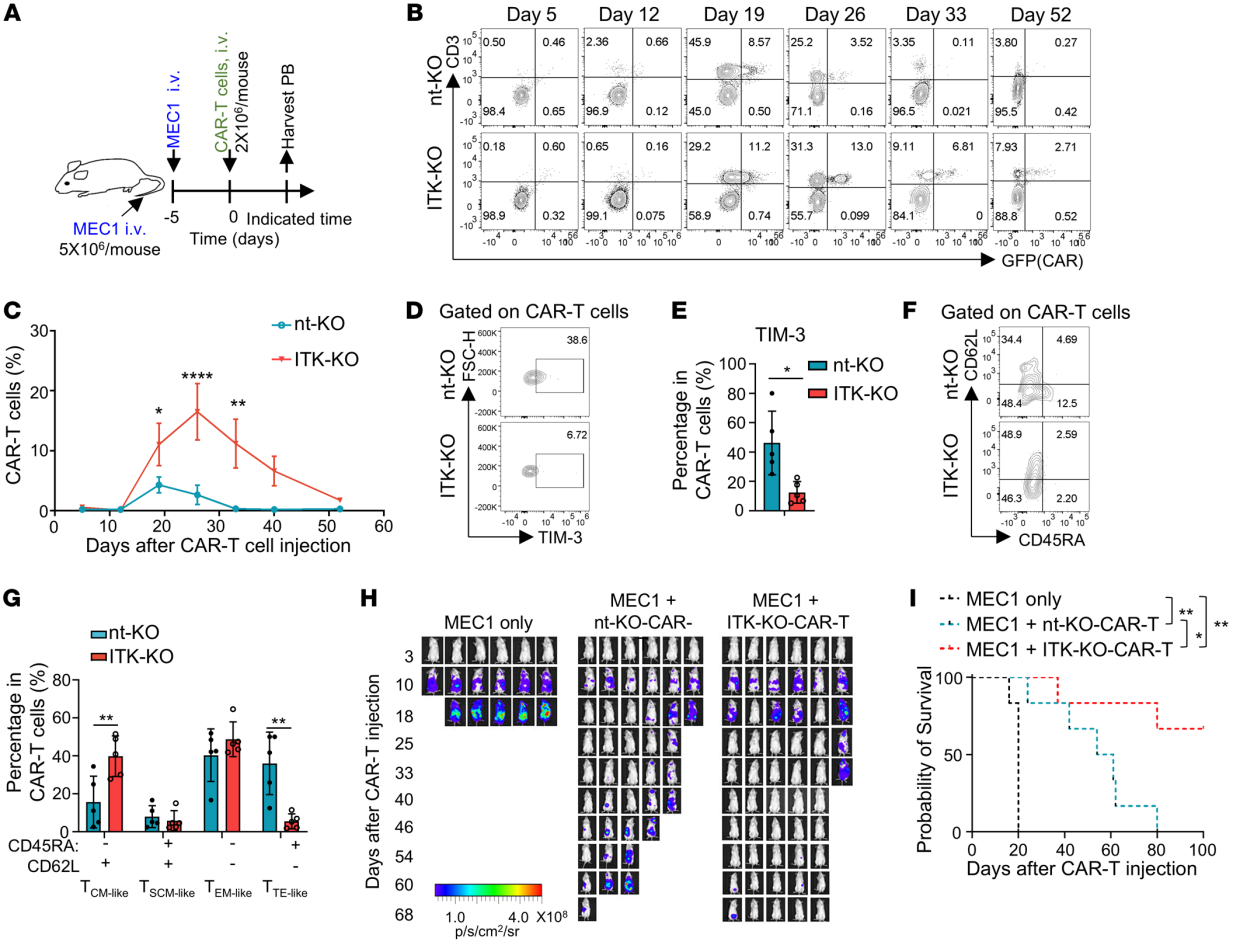
ITK*-*deficient CAR-T cells derived from CLL patients improve control of tumor relapse in vivo. (**A**) Experimental design of CAR-T cell therapy against intravenously injected MEC1 cells expressing luciferase in NPG mice via the lateral tail vein. CAR-T cells were expanded for 12 days following electroporation before intravenous injection into mice. (**B**) Representative flow cytometric plots of CAR-GFP and CD3 in PBMCs collected from CLL-CAR-T cell recipients at the indicated time points. (**C**) Summary of percentages of CAR-T cells (CD3^+^GFP^+^) as shown in **B** (mean ± SEM; *n* = 5 for the nt-KO groups on days 26, 33, and 40, as well as the ITK-KO groups on days 40 and 52; *n* = 4 for nt-KO day 52 group; *n* = 6 for the rest). (**D**) Representative flow cytometric plots of TIM-3 expression on CAR-T cells in PBMCs collected from nt-KO or ITK-KO CLL-CAR-T recipients 26 days after infusion. (**E**) Summary of percentages of CAR-T cells that are TIM-3^+^ as shown in **D** (*n* = 5). (**F**) Representative flow cytometric plots of CD62L and CD45RA expression in the indicated CAR-T cells collected. PBMCs were collected on day 33 after CAR-T cell injection. (**G**) Statistical analysis of different cell populations as shown in **F** (*n* = 5). (**H**) Representative bioluminescence images of NPG mice xenografted with MEC1 cells as designed in **A**. (**I**) Survival of MEC1-bearing NPG mice treated with PBS or nt-KO CLL-CAR-T or ITK-KO CLL-CAR-T cells (*n* = 6) (log-rank Mantel-Cox test with Bonferroni’s correction for multiple comparisons). Compiled data from 2 independent experiments in **C**, **E**, **G**, and **I**. Statistical differences were determined by 2-tailed unpaired Student’s *t* test in **C**, **E**, and **G**. Data represent results of 2 independent experiments. *P < 0.05, **P < 0.01, ****P < 0.0001.

To assess the long-term in vivo effects of *ITK* deletion on CLL-CAR-T cells, we employed a CLL mouse model using NPG mice injected intravenously with MEC1 tumor cells, followed by infusion with ITK-KO or nt-KO CLL-CAR-T cells (Figure 8A). The results showed that ITK-KO CLL-CAR-T cells exhibited better expansion and long-term persistence compared with nt-KO CLL-CAR-T cells (Figure 8, B and C). Additionally, ITK-KO CLL-CAR-T cells showed increased proliferation and reduced apoptosis compared with nt-KO CLL-CAR-T cells (Supplemental Figure 8, A–D). There was reduced exhaustion and enhanced memory phenotype in ITK-KO CLL-CAR-T cells compared with nt-KO CLL-CAR-T cells (Figure 8, D–G, and Supplemental Figure 8E). Furthermore, ITK-KO CLL-CAR-T cells showed better control of tumor relapse (Figure 8, H and I, and Supplemental Figure 8F). Notably, mouse body weights were similar between the groups treated with ITK-KO and nt-KO CLL-CAR-T cells (Supplemental Figure 8G). These results suggest that CLL-CAR-T cells with ITK deficiency exhibit enhanced efficacy in controlling tumor relapse.

Altogether, our results suggest that ITK deficiency could reduce exhaustion and promote memory in CD19-CAR-T cells, improving the expansion and long-term persistence of CAR-T cells in vivo. This contributes to better control of tumor relapse and potentially improves clinical outcomes.

## Discussion

CAR-T cell therapy has emerged as a ground-breaking treatment for various hematologic malignancies. However, the efficacy and long-term sustainability of CAR-T cells remain challenging because of issues such as T cell exhaustion, relatively short persistence in vivo, and antigenic escape (45, 46). Furthermore, CAR-T cell therapy faces a substantial hurdle given its autologous nature, which necessitates using a patient’s own T cells for CAR-T cell production. Obtaining a sufficient quantity and quality of T cells proves exceedingly difficult, primarily owing to the occurrence of T cell lymphopenia and exhaustion prevalent in cancer patients, including those with chronic lymphocytic leukemia (CLL) (47). These limitations cause concerns about the safety, efficacy, and accessibility of CAR-T cell therapy. Our data provide direct evidence for the cell-intrinsic role of ITK in regulation of expansion and memory development, as well as exhaustion, in CAR-T cells. Deletion of *ITK* during CAR-T cell production leads to enhanced CAR-T cell expansion and memory development, along with reduced cell apoptosis and exhaustion upon repeated exposures to tumor cells. This improved CAR-T cell sustainability is of particular interest for controlling tumor relapse.

One of the interesting findings of this study is the attenuation of immediate cytotoxicity in ITK-deficient CAR-T cells against tumor cells. While these cells exhibit slightly reduced cytotoxicity against tumor cells, they do not show a significant decline in the production of critical effector cytokines such as IFN-γ, TNF-α, and granzyme B. This suggests that ITK deficiency primarily affects the immediate cytotoxic effects of CAR-T cells, which may not be as critical for long-term antitumor responses. Indeed, previous studies demonstrated that ITK is not required for T cell activation, but it rather promotes the strength of TCR signaling and thus TCR-dependent cytotoxic T cell function (48). This ITK-mediated strong TCR signaling can tune down T cell responses to cytokines that can drive T cell expansion, survival, and memory development, such IL-2 and IL-4 (49).

Our study reveals that ITK-deficient CAR-T cells exhibit enhanced expansion and long-term survival in response to tumor antigen stimulation. This is crucial for maintaining therapeutic efficacy over extended periods. While initial expansion rates are similar between ITK-deficient and control CAR-T cells, the former outperform at later stages. This could be attributed to reduced apoptosis and prolonged proliferation in ITK-deficient CAR-T cells. Regarding the in vitro expansion of CAR-T cells, it appears that CD4^+^ T cells exhibit greater improvement for expansion compared with CD8^+^ T cells following *ITK* knockout. This difference might be due to the varying expression levels of IL-2 receptors on CD4^+^ T cells and CD8^+^ T cells (50). During the in vitro expansion of CAR-T cells, a relatively high level of IL-2 was added to the culture medium to produce sufficient CAR-T cells for clinical use (51–54). Given the higher expression of IL-2 receptors in CD8^+^ T cells (50), the extent of IL-2 downstream signaling activation might be closer to saturation in CD8^+^ T cells than in CD4^+^ T cells.

In addition, our current knowledge of the role of ITK in T cell exhaustion is very limited. Importantly, this study shows that ITK deficiency leads to reduced T cell exhaustion, as evidenced by lower expression of exhaustion markers such as LAG-3, PD-1, TIM-3, TIGIT, and CTLA4. Moreover, the transcriptomic and scRNA-Seq analyses indicate that ITK-KO CAR-T cells are characterized by a more marked central memory phenotype, which could contribute to their sustained antitumor activity. Recently it has been demonstrated that tumor-infiltrating TCF1^+^ T cells have long-term memory, and are capable of self-renewal and persistent control of tumor growth. It is possible that the upregulation of TCF1, a dominant transcription factor negatively regulating T cell terminal differentiation and exhaustion, observed in ITK-KO CAR-T cells might prevent the progressive exhaustion and maintain a long-term memory.

We showed that while pharmacological ITK inhibition could mitigate T cell exhaustion and promote memory phenotype in CAR-T cells derived from patients with CLL, it only showed a trend but no significant increase of CLL-CD19-CAR-T cells in the tumor-bearing mouse model in vivo. These observations may suggest that transient inhibition of ITK may not be enough to maintain CAR-T cell status with low exhaustion and better memory. Genetic deletion or silencing of *ITK* at least for a certain period is probably required to efficiently and persistently reduce T cell exhaustion and promote T cell memory in CAR-T cells. Notably, the control of relapse varied among individual tumor-bearing mice, which might be attributed to biological variation in the recipient mice. This variability suggests that clinical outcomes might differ among patients when ITK-deficient CAR-T cells are used to manage tumor relapse. Exploring host factors that might influence the effectiveness of ITK-deficient CAR-T cells in controlling tumor relapse would be a valuable direction for future research.

Previous studies have shown that transient treatment with dasatinib reduces expression of exhaustion markers and increases expression of stem cell memory–associated markers, and improves tumor clearance (18, 19). Investigation of whether long-term dasatinib treatment or permanent deletion of dasatinib targets could enhance CAR-T cell persistence and better control tumor relapse in vivo would be an intriguing area for future research.

Since lentivirus transduction and electroporation, which might act as stimulatory factors affecting T cell activation and function, are commonly used in CAR-T cell production and gene editing, examining their overall impact on CAR-T cells in further studies would be beneficial. Additionally, although we selected an ITK-targeting sgRNA with fewer overall off-target effects and with the top predicted off-target sites outside the coding sequences of known genes, potential off-target effects in introns or intergenic regions still should be considered, as these regions may affect gene expression (55).

It has been reported that ITK deficiency may impact Th17 development and promote Treg generation (56–60). Additionally, several studies have shown that *Itk^–/–^* CD4^+^ T cells exhibit defects in producing Th2 cytokines in mice (61, 62). Our findings indicate that the expression of IL-17A, IL-4, IL-13, and FOXP3 was low in the CAR-T cells, with minimal differences observed between ITK-KO and nt-KO CAR-T cells. The low expression of these cytokines may be attributed to the in vitro culture environment, where CAR-T cells are exposed to tumor cells, potentially favoring Th1 cytokine expression. However, the effects of ITK on the expression of these molecules still should be further considered in clinical applications involving human CAR-T cells, which may encounter more complex environmental conditions. The expected inhibition of Th2 and Th17 and favoring of Treg-generation effects by ITK deficiency in CAR-T cells may potentially result in attenuated cytokine release syndrome (CRS) and immune effector cell–associated neurotoxicity syndrome (ICANS) in clinical settings. Moreover, the absence of ITK has been shown to attenuate T cell migration to several peripheral organs, such as the intestine and brain (63–66), which may further contribute to reduced CRS and ICANS during ITK-KO CD19-CAR-T cell therapy. Investigating these aspects in future studies could provide valuable insights.

In conclusion, this study demonstrates that ITK deficiency enhances expansion and reduces exhaustion of CAR-T cells and improves their long-term therapeutic effects. These findings offer promising insights into the development of more effective and sustainable CAR-T cell therapies for a broad spectrum of cancers. Further research and clinical trials are warranted to validate the clinical applicability of targeting ITK in CAR-T cell therapy.

## Methods

### Sex as a biological variable.

Our study examined male and female animals, and similar findings are reported for both sexes.

### Mice.

NOD.Cg-Prkdc^scid^ Il2rg^tm1Vst^/Vst (NPG) mice were from Beijing Vitalstar Biotechnology and ranged from 6 to 8 weeks of age. Experiments were performed with both male and female mice unless otherwise indicated. For MEC1 cell–derived xenograft models, NPG mice were implanted with 1.0 × 10^7^ MEC1 cells expressing firefly luciferase intraperitoneally in the left flank, or with 1.0 × 10^7^ MEC1 cells subcutaneously, or with 5.0 × 10^6^ MEC1 cells expressing firefly luciferase via the lateral tail vein, using a 27-gauge needle. Five to ten days after engraftment, 2.0 × 10^6^ nt-KO or ITK-KO CAR-T cells were intravenously administered to tumor-bearing mice via the lateral tail vein. For Raji cell–derived xenograft models, NPG mice were implanted with 5 × 10^5^ Raji cells expressing firefly luciferase intraperitoneally in the left flank. Ten days later, tumor-bearing mice were intravenously injected with 5.0 × 10^6^ nt-KO or ITK-KO CAR-T cells via the lateral tail vein. After CAR-T cell injection, blood samples were collected from the orbital venous plexus of mice at indicated time points. Red blood cells were removed using ACK lysis buffer (catalog A1049201, Gibco). Then PBMCs were processed for flow cytometry analysis. Mice injected with MEC1 cells subcutaneously were monitored 3 times a week using a square caliper to measure tumor growth (tumor volume = π/6 × length × width × height) as previously described (67). Mice injected with MEC1 or Raji cells intraperitoneally or intravenously were monitored by in vivo imaging (IVIS Lumina III, PerkinElmer) at indicated time points. d-Luciferin potassium salt (catalog 122799, PerkinElmer) was dissolved in PBS to create a working solution at 15 mg/mL. All mice received an intraperitoneal injection of luciferin solution (150 mg/kg) 10 minutes before in vivo imaging.

### Human T cell isolation and CAR-T cell production.

Blood samples were obtained from the Second Xiangya Hospital. Ficoll-Paque (catalog 25710, Dongfang Huahui Co. Ltd.) was used to isolate PBMCs as previously reported (68). PBMCs were further used for T cell enrichment using an EasySep Human T Cell Isolation Kit (catalog 17951, STEMCELL Technologies) following the manufacturer’s instructions. The enriched T cells were activated with Dynabeads Human T-Activator CD3/CD28 (catalog 11132D, Gibco; T cells/beads = 1:2) for 24 hours and transduced with CAR-encoding lentivirus (multiplicity of infection = 8). Twenty-four hours after CAR transduction, the lentivirus-containing medium was replaced with X-VIVO15 complete medium supplemented with IL-2 (catalog 200-02, PeproTech; 100 U/mL). Gene editing in CAR-T cells by electroporation of RNP complex was performed 48–72 hours after CAR transduction. After gene editing, CAR-T cells were cultured for an additional 3–5 days before in vitro analysis or further expansion, or for an additional 11–14 days before injection into mice in the in vivo mouse experiments, unless otherwise noted.

### Gene editing in CD19-CAR-T cells.

CRISPR-mediated gene editing was used to delete *ITK*. Single-guide RNA (sgRNA) sequences were designed using the CRISPick online tool (https://portals.broadinstitute.org/gppx/crispick/public, Broad Institute) to achieve SpyCas9-mediated CRISPR knockout. Two sgRNAs (named ITK-sg1 and ITK-sg2; Supplemental Table 1) were selected along with a nontargeting control sgRNA (named nt-sg1) (69) and synthesized (GenScript Co. Ltd., Nanjing, China). Cas9 protein was purchased from Advanced Biomart (catalog CCN-066AB) and was delivered by nucleofection as an RNP complex with sgRNAs. Briefly, 3 days after CAR transduction, anti-CD3/CD28 beads were removed, and 5 × 10^6^ CAR-T cells were resuspended in 100 μL electroporation buffer (82 μL P3 Primary Cell Solution mixed with 18 μL Supplement; catalog V4XP-3024, Lonza). Cas9 protein (30 μg) was gently mixed with specific sgRNA (30 μg), placed at room temperature for 15 minutes, mixed with cells, transferred to Nucleocuvett Vessels, and electroporated using a 4D-Nucleofactor (catalog AAF-1003X, Lonza) with program EO115, followed by CAR-T cell culture and expansion in prewarmed X-VIVO15 medium. Three days later, genomic DNA was extracted and used for validation by Sanger sequencing. Gene editing efficiency and potential off-target effects were validated by PCR (Supplemental Tables 1 and 2, respectively) and subsequently Sanger sequencing. The obtained sequencing results were analyzed with the TIDE online tool (http://shinyapps.datacurators.nl/tide/) to calculate gene editing efficiency, with a threshold set at a *P* value of 0.001. SnapGene v6.01 was used to read the Sanger sequencing results of DNA PCR products.

### In vitro killing assay.

MEC1 (catalog CL-0761, Pricella), HG3 (catalog ACC765, DSMZ), and Raji cells (catalog CL-0189, Pricella) stably expressing firefly luciferase were obtained via lentiviral transduction with the pLVX-Luc2-puro plasmid (Ningbo Testobio Co. Ltd., TSPLA10184) following selection with 2 μg/mL puromycin (catalog P8230, Solarbio). CAR-T cells (effector, E) were cocultured with tumor cells (target, T) at indicated ratios in a 96-well cell culture plate for 48 hours, and cancer cell lysis was detected using the Steady-Glo Luciferase assay system (catalog E2520, Promega). The percentage of specific lysis was calculated as:

 (Equation 1)
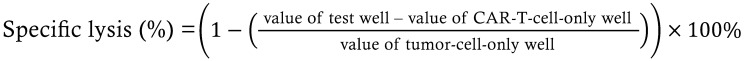


For serial tumor killing assays, CAR-T cells were cocultured with MEC1 cells at an E/T ratio of 2:1 in a 96-well plate. Bioluminescence was measured every 48 hours, and the E/T cell ratio was readjusted to 2:1 for coculture after each sampling. Nine rounds of analyses were performed.

### RNA sequencing and data analyses.

CAR-T cells were cocultured with MEC1 cells at an E/T ratio of 2:1 in X-VIVO15 medium with 5% FBS at 37°C with 5% CO_2_ for 48 hours. After incubation, GFP^+^ CAR-T cells were FACS-sorted, and the purified nt-KO or ITK-KO CAR-T cells were used for both bulk and scRNA-Seq and analyses as previously described (70) and detailed in Supplemental Methods.

### Statistics.

Two-tailed unpaired Student’s *t* test was performed for statistical analysis by GraphPad Prism 6.01 software unless otherwise noted. Log-rank (Mantel-Cox) test with Bonferroni’s correction for multiple comparisons was performed for statistical analysis of the survival curves with GraphPad Prism 6.01 software. Statistical analyses of solid tumor growth in the mouse model with subcutaneous injection of MEC1 cells were conducted by linear mixed-effects modeling (over the whole time course) with Bonferroni’s correction for multiple comparisons as described previously (71). Data for statistical analyses are presented as mean ± SD unless otherwise noted. *P < 0.05, **P < 0.01, ***P < 0.001, ****P < 0.0001.

### Study approval.

All animal experiments were approved by the Institutional Animal Care and Use Committee of the Second Xiangya Hospital and the Suzhou Institute of Systems Medicine, Chinese Academy of Medical Sciences (CAMS-ISM) animal facility. This study involved human samples and was approved by the Institutional Review Board at the Second Xiangya Hospital [reference 052 (2018)]. Participants in the study gave informed consent in accordance with the Declaration of Helsinki before taking part.

### Data availability.

The bulk RNA-Seq and scRNA-Seq data in this study were deposited in the NCBI’s Gene Expression Omnibus database under accession codes GSE278601 and GSE278612, respectively. Values for data points in figures are reported in the Supporting Data Values file. Other data and materials are available upon reasonable request.

## Author contributions

WH and ZF conceived the research idea. ZF, HP, and WH designed the study. HP, ZF, QS, and WH supervised the study. ZF, ZH, HX, QL, JL, YD, HZ, PW, HL, LH, YF, CW, and KS acquired the data. ZH, HX, QS, SKC, KY, AZ, KS, WH, and ZF analyzed the data. ZF, WH, HP, QS, ZH, AA, GV, AZ, YT, YS, ZL, and KS prepared the manuscript. ZF is listed first among the co–first authors because ZF was responsible for all experimental designs, performed the animal experiments, analyzed and interpreted data, and drafted and revised the manuscript. ZH is listed second among the co–first authors because ZH participated in all the research, collected, analyzed, and interpreted data, and revised the manuscript.

## Supplementary Material

Supplemental data

Unedited blot and gel images

Supplemental video 1

Supplemental video 2

Supplemental video 3

Supporting data values

## Version 1 11/26/2024

In-Press Preview

## Version 2 02/17/2025

Electronic publication
