# Supplementary material for: IL-2–inducible T cell kinase deficiency sustains chimeric antigen receptor T cell therapy against tumor cells
Source: J Clin Invest. 2024 Nov 26;135(4):e178558. doi: 10.1172/JCI178558 (PMC11827851; doi:10.1172/JCI178558)
Supplement: Supplemental data [file jci-135-178558-s240.pdf]

SUPPLEMENTAL MATERIALS

IL-2-inducible T cell kinase deficiency sustains chimeric antigen receptor T cell therapy against tumor cells

Authors

Zheng Fu, [1, 2, 9, 16] Zineng Huang, [1, 3, 4] Hao Xu, [5] Qingbai Liu, [6] Jing Li, [7] Keqing Song, [8] Yating Deng, [1, 3, 4] Yujia Tao, [1, 3, 4] Huifang Zhang, [1, 3, 4] Peilong Wang, [1, 3, 4] Heng Li, [1, 3, 4] Yue Sheng, [1, 3, 4] Aijun Zhou, [6] Lianbin Han, [9] Yan Fu, [9] Chenzhi Wang, [9] Saurav Kumar Choudhary, [10] Kaixiong Ye, [10, 11] Gianluca Veggiani, [12] Zhihong Li, [13, 14] Avery August, [15] Weishan Huang, [12, 15] Qiang Shan, [5] Hongling Peng, [1, 3, 4, 14]

Author affiliation

[1] Department of Hematology, The Second Xiangya Hospital, Central South University, Changsha, Hunan 410011, PR China

[2] Hubei Jiangxia Laboratory, Wuhan, Hubei 430200, PR China

[3] Institute of Hematology, Central South University, Changsha, Hunan 410011, PR China

[4] Hunan Engineering Research Center of Cell Immunotherapy for Hematopoietic Malignancies, Changsha, Hunan 410011, PR China

[5] National Key Laboratory of Immunity and Inflammation, Suzhou Institute of Systems Medicine, Chinese Academy of Medical Sciences & Peking Union Medical College, Suzhou, Jiangsu 215123, PR China

[6] Lianshui People's Hospital of Kangda College Affiliated to Nanjing Medical University, Huai'an, Jiangsu Province, PR China

[7] Key Laboratory of Cluster Science, Ministry of Education of China, School of Chemistry and Chemical Engineering, Beijing Institute of Technology, Beijing, 100081, PR China

[8] Tianjin Mogenetics Biotech Co., Ltd, Tianjin, 300457 PR China

[9] MegaRobo Technologies Co., Ltd, 277 Dongping Road, Suzhou, 215000, PR China

[10] Institute of Bioinformatics, University of Georgia, Athens, GA, 30602, USA

[11] Department of Genetics, Franklin College of Arts and Sciences, University of Georgia, Athens, GA, 30602, USA

[12] Department of Pathobiological Sciences, School of Veterinary Medicine, Louisiana State University, Baton Rouge, LA 70803, USA

[13] Department of Orthopedics, The Second Xiangya Hospital, Central South University, Changsha, PR China

[14] Hunan Key Laboratory of Tumor Models and Individualized Medicine, Changsha, Hunan 410011, PR China

[15] Department of Microbiology and Immunology, College of Veterinary Medicine, Cornell University, Ithaca, NY 14853, USA

[16] Xinyi Biotech Co., Ltd, Lingang, Shanghai, 201306, PR China

Authorship note: ZF, ZH and HX contributed equally to this work. HP, ZF, QS, and WH jointly supervised this work.

Address correspondence to: Hongling Peng, Department of Hematology, The Second Xiangya Hospital, Central South University, Changsha, Hunan 410011, PR China. Phone: +86 0731-85295296; Email: penghongling@csu.edu.cn. Or to: Zheng Fu, Hubei Jiangxia Laboratory, Wuhan, Hubei 430200, PR China. Phone: +86 18120040001; Email: fuzheng_ioz@163.com. Or to: Qiang Shan, National Key Laboratory of Immunity and Inflammation, Suzhou Institute of Systems Medicine, Chinese Academy of Medical Sciences & Peking Union Medical College, Suzhou, Jiangsu 215123, PR China. Phone: +86 0512-62873785; Email: shanq2009@gmail.com. Or to: Weishan Huang, Department of Pathobiological Sciences, School

of Veterinary Medicine, Louisiana State University, Baton Rouge, LA 70803, USA. Phone: +01-225-5789467; Email: huang1@lsu.edu.

MATERIALS AND METHODS

Plasmids and lentivirus production

Lentiviral packaging plasmids psPAX2 (Cat#12260, Addgene), pMD2.G (Cat#12259, Addgene), and the CD19-CAR-expressing plasmid (Cat#LC0001-3, Public Protein/Plasmid Library) or GD2-CAR-expressing plasmid (Cat#LC0003-3, Public Protein/Plasmid Library) were used for production of CAR-expressing lentivirus. 293T packaging cells were seeded in DMEM (Cytiva) complete medium (supplemented with 10% FBS and 1% Penicillin/Streptomycin) in a 15 cm dish and incubated until cells reaching 80–90% confluence. For transfection, 15 μg of psPAX2, 15 μg of pMD2.G and 30 μg of the CAR-expressing plasmid were mixed in a tube with 3.75 mL of Opti-MEM (Cat#31985070, GIBCO). In a second tube, 120 μg of polyethylenimine (PEI) (Cat#24765-1, Polysciences) was added to 3.75 mL of Opti-MEM. Both mixtures were incubated for 5 minutes at room temperature, then mixed and incubated for an additional 25 minutes. The cell medium was replaced with DEME medium one hour before transfection. Transfection mixture was added to the dish and replace the media with DMEM complete 6-8 hours post-transfection. Viral supernatant was harvested at 24, 48 and 72 hours, filtered through a 0.45 μm filter, concentrated with a centrifugal filter unit (Cat# UFC910024, Millipore) at 4500 RPM for 30 minutes at 4°C, then aliquoted and stored at –80°C.

sgRNA, predicted off-target sequences and primers for validation of editing efficiency

The CRISPick online tool (https://portals.broadinstitute.org/gppx/crispick/public, Broad Institute) was used for single guide RNA (sgRNA) design using the human GRCh38 as the reference genome for SpyoCas9-mediated CRISPRko. Two sgRNAs (named ITK-sg1 and ITK-sg2, see Supplemental Table 1) were selected along with a non-targeting control sgRNA (named nt-sg1) (1), and synthesized by GenScript (Nanjing, China). To predict off-target effects, the online tool CRISPOR (http://crispor.gi.ucsc.edu/crispor.py) was used to query the top 4 potential off-target

sites (see Supplemental Table 2) of ITK-sgRNA1 and ITK-sgRNA2. Predicted off-target sites were experimentally validated by amplification of genomic DNA using off-target-specific primers and subjecting the amplicons to Sanger sequencing. Briefly, PCR primers (primers used were listed in Supplemental Table 2) were designed using the online tool Primer3 (https://bioinfo.ut.ee/primer3-0.4.0/) ensuring annealing to target sequence about 100-200 bp upstream the sgRNA-targeted DNA region. PCR reactions were performed using Q5 High-Fidelity DNA Polymerase (Cat#M0491V, NEB) and following 30 cycles of amplification, amplicons underwent Sanger sequencing (Shengong, Co. Ltd., Shanghai, China). Obtained sequences were analyzed with the TIDE (http://shinyapps.datacurators.nl/tide/) online tool to estimate gene editing efficiency, using a P-value threshold of 0.001.

Flow cytometry analysis

ACK lysis buffer (Cat#A1049201, Gibco) was used to remove red blood cells from whole blood as per manufacturer's instructions. To stain surface makers, cells were firstly stained with LIVE/DEAD Fixable Aqua Dead Cell Stain Kit (Cat#L34966, Invitrogen), followed by Fc-receptor blocking (Cat# 422301, Biolegend), and then stained with indicated antibody (1:200 final dilution unless otherwise noted) for 30 minutes at 4°C. Annexin V-PE/7-AAD Apoptosis Detection Kit (Cat#A213-01, Vazyme Biotech Co., Ltd.) was used for apoptosis detection as per manufacturer's instructions. To stain intracellular proteins, after surface staining, cells were pre-fixed in 4% paraformaldehyde for 10 minutes, fixed and permeabilized with Foxp3 / transcription factor staining buffer (Cat#00-5523-00, eBioscience), and then stained with antibodies for 2 hours at 4°C. For cytokine staining, cells were stimulated with 50 ng/ml PMA (Cat# HY-18739, MedChemExpress) and 500 ng/ml ionomycin (Cat#73724, StemCell Technology) for 2 hours or tumor cells as indicated, and Brefeldin A (2μg/ml; Cat#HY-16592, MedChemExpress) was added 2 hours before cells were harvested. Antibodies used for flow cytometry are detailed in the

Supplemental Materials. Cytek Aurora spectral flow cytometer was used for data collection and FlowJo (version 10.6) was used for FACS data analysis.

Antibodies used for flow cytometry

Antibodies used: anti-human CD69 (Cat# 310922, Biolegend), anti-human LAG-3 (Cat# 369320, Biolegend), anti-human PD-1 (Cat# 15-2799-42, eBioscience), anti-human TIM-3 (Cat# 345008, Biolegend), anti-human CTLA-4 (Cat# 46-1529-42, eBioscience), anti-human TIGIT (Cat# 372704, Biolegend), anti-human CD45RA (Cat# 304134, Biolegend), anti-human CD45RO (Cat# 304237, Biolegend), anti-human CCR7 (Cat# 353254, Biolegend), anti-human Ki-67 (Cat# 350526, Biolegend), anti-human CD19 (Cat# 392504 and Cat# 302218, Biolegend), CD62L (Cat# 304809, Biolegend), IFN-γ (Cat# 506541, Biolegend), Granzyme B (Cat# 12-8899-41, eBioscience)、TNF-α (Cat# 502920, Biolegend)、IL-17A (Cat# 45-7179-42, eBioscience)、FOXP3 (Cat# 56-4776-41, eBioscience), IL-4 (Cat# 500808, Biolegend), IL-13 (Cat# 501913, Biolegend), CD3ε (Cat# 317336, Biolegend), CD4 (Cat# 47-0048-42 and Cat# 48-0049-42, eBioscience), CD8α (Cat# 25-0088-42, eBioscience).

Western blotting

CAR-T cells were lysed using RIPA lysis buffer (Cat#P0013B, Beyotime Biotechnology Co., Shanghai, China) with protease inhibitor cocktail (Cat#P1005, Beyotime Biotechnology). Protein concentrations were measured via BCA Protein Quantification Kit (Cat# E112-01, Vazyme Biotech Co., Ltd.). Samples were heat-denatured in loading buffer, separated by electrophoresis and transferred to PVDF membranes, followed by incubation with primary antibodies against ITK (Cat# 77215S, CST), PLCγ-1(Cat#5690, CST), phospho-PLCγ-1(Cat#2821S, CST), ERK1/2(Cat#9102S, CST), phospho-ERK1/2(Cat#9101S, CST), p70S6(Cat#2708T, CST), phospho-p70S6(Cat#9205S, CST) and GAPDH (Cat#97166, CST) as per manufacturer's

instructions. HRP-conjugated secondary antibodies (Cat#ab6721 and ab6789, Abcam) were used as recommended and chemiluminescence reagents (Cat#P0018FS, Beyotime Biotechnology) was used for visualization on a gel imaging system (Cat#NA, Baygene Biotech Co. Ltd., Beijing, China).

Bulk RNA sequencing and data analyses

Total RNA extraction was performed for 1 × 10^6 sorted cells derived from each experimental group using the FastPure cell/tissue total RNA isolation kit (CAT# RC112-01, Vazyme Biotech Co., Ltd.) according to the manufacturer's instructions. RNA-seq libraries were constructed and sequenced commercially by Azenta Life Science Co. Ltd (Suzhou, China). The adaptor sequence of RNA-seq raw reads was trimmed using trimmomatic-0.36 (2), and reads were aligned to hg38 genome using HISAT2 (3). Raw gene counts were generated using the subread featureCounts function (4), normalized for transcripts per kilobase per million and normalized bigwigs were generated for visualization using Integrative Genomics Viewer (5). Differential expression analysis was defined with log2 (fold change) ≥1.5 and a false discovery rate ≤ 0.05 was applied by using the R package DESeq2 (6). Gene set enrichment analysis (GSEA, v4.3.2) was performed using the RPKM data as input (7). GOLDRATH_EFF_VS_MEMORY_CD8_TCELL_DN gene set from MSigDB Signatures was used as reference of GSEA (8, 9). One thousand random permutations of the phenotypic subgroups were used to establish a null distribution of enrichment score against which NES and FDR-corrected q values were calculated. GO and KEGG pathway enrichment analysis of the DEGs were conducted by GOSeq R package (1.24.0) (10) and KOBAS software (3.0) (11), respectively.

Single-cell RNA sequencing and data analyses

Similar to bulk RNA-seq sample preparation, CAR-T cells were co-cultured with MEC1 at an E: T = 2:1 for 48 hours, and GFP-positive CAR-T cells were obtained via FACS. Single-cell RNA-Seq

was performed following manufacturer's protocols (https://www.10xgenomics.com). In brief, single cell suspension at 1000 cells/μl in PBS was loaded onto a Chromium controller for GEM (Gel Beads-In-Emulsion) generation and barcoding. Gene expression library was constructed with the chromium single cell 3' reagent kit v2 (10x Genomics). The post-library product was qualified and sequenced on an Illumina Novaseq 6000 by Genergy Biotechnology Co. Ltd. (Shanghai, China). Sequencing data alignment and gene expression matrix were performed by cellranger 5.0.1 (10X Genomics, San Francisco, CA) by aligning them to a pre-built human reference genome hg38 and human gene annotation version 32 (Ensembl 92). Cells with a percentage of mitochondrial genes >25%, expressed genes <100 or >5,000 and estimated as doublets by the scds package were removed from further analysis. The Seurat package (version 4.1.1) was adopted for dimension reduction and unsupervised cell clustering (12). Before processing, mitochondrial and ribosomal genes were removed, and the 3,000 most variable genes were selected for integrating data from different batches and for principal component (PC) analysis. The first 30 PCs were used for identifying cell clusters with a resolution of 0.5. In order to obtain confidential clusters with more features, a cluster with an average feature number of less than 1,000 and a median read percentage of mitochondrial genes > 10% was excluded from downstream analysis. Clustering results were illustrated in UMAP space. Variable genes were selected based on average expression and dispersion. Marker genes were identified with Seurat's FindAllMarkers function. Top 100 high-expressed genes in each cluster were shown in Supplemental Table 3. Normalized gene expression data were shown as feature plots or violin plots and scaled expression data of cluster marker genes were used to generate heatmaps.

Drug treatment of CLL-CAR-T cells

CLL-CAR-T cells were treated with PF-06465469 (1 μM, Cat# HY-108691, MedChemExpress), ibrutinib (0.78 μM, Cat# HY-10997, MedChemExpress), or dimethyl sulfoxide (DMSO) for an additional 5-7 days after coculture with MEC1 cells for 48 hours. For the mouse experiments,

CLL-CAR-T cells were treated with ibrutinib (0.78 μM) or DMSO for 12 days following CAR-expressing lentivirus infection.

Continuous monitoring of tumor killing by CAR-T cells

nt-KO CD19-CAR-T cells and ITK-KO CD19-CAR-T cells were sorted based on GFP positive. MEC1 cells were used for co-culture experiments. In each well of a flat-bottom 96-well plate (pre-coated with 0.01% poly-L-ornithine, Cat# P3655, Sigma), 20,000 MEC1 cells and 40,000 CD19-CAR-T cells were added. Cells were cultured in X-VIVO15 medium suppled with IL-2 (100 U/mL, Peprotech). CellEvent™ Caspase-3/7 Red Detection Reagent (Cat# C10430, Thermo) was incorporated into the culture medium at the start of the co-culture, following the manufacturer's instructions. During the co-culture, pro-apoptotic cells were labeled with red fluorescence, which would finally disappear as the apoptotic cells underwent cell death. A total of 48 hours continuous monitoring was performed using Incucyte® Live-Cell Analysis (Sartorius).

REFERENCES

1. Ting PY, Parker AE, Lee JS, Trussell C, Sharif O, Luna F, et al. Guide Swap enables genome-scale pooled CRISPR-Cas9 screening in human primary cells. *Nat Methods.* 2018;15(11):941-6.
2. Bolger AM, Lohse M, and Usadel B. Trimmomatic: a flexible trimmer for Illumina sequence data. *Bioinformatics.* 2014;30(15):2114-20.
3. Kim D, Paggi JM, Park C, Bennett C, and Salzberg SL. Graph-based genome alignment and genotyping with HISAT2 and HISAT-genotype. *Nature Biotechnology.* 2019;37(8):907-15.
4. Liao Y, Smyth GK, and Shi W. featureCounts: an efficient general purpose program for assigning sequence reads to genomic features. *Bioinformatics.* 2013;30(7):923-30.
5. Robinson JT, Thorvaldsdóttir H, Winckler W, Guttman M, Lander ES, Getz G, et al. Integrative genomics viewer. *Nature Biotechnology.* 2011;29(1):24-6.
6. Love MI, Huber W, and Anders S. Moderated estimation of fold change and dispersion for RNA-seq data with DESeq2. *Genome Biology.* 2014;15(12):550.
7. Mootha VK, Lindgren CM, Eriksson K-F, Subramanian A, Sihag S, Lehar J, et al. PGC-1α-responsive genes involved in oxidative phosphorylation are coordinately downregulated in human diabetes. *Nature Genetics.* 2003;34(3):267-73.
8. Subramanian A, Tamayo P, Mootha VK, Mukherjee S, Ebert BL, Gillette MA, et al. Gene set enrichment analysis: a knowledge-based approach for interpreting genome-wide expression profiles. *Proc Natl Acad Sci U S A.* 2005;102(43):15545-50.
9. Mootha VK, Lindgren CM, Eriksson KF, Subramanian A, Sihag S, Lehar J, et al. PGC-1alpha-responsive genes involved in oxidative phosphorylation are coordinately downregulated in human diabetes. *Nat Genet.* 2003;34(3):267-73.
10. Young MD, Wakefield MJ, Smyth GK, and Oshlack A. Gene ontology analysis for RNA-seq: accounting for selection bias. *Genome Biology.* 2010;11(2):R14.
11. Bu D, Luo H, Huo P, Wang Z, Zhang S, He Z, et al. KOBAS-i: intelligent prioritization and exploratory visualization of biological functions for gene enrichment analysis. *Nucleic Acids Research.* 2021;49(W1):W317-W25.
12. Satija R, Farrell JA, Gennert D, Schier AF, and Regev A. Spatial reconstruction of single-cell gene expression data. *Nature Biotechnology.* 2015;33(5):495-502.
13. Enot DP, Vacchelli E, Jacquelot N, Zitvogel L, and Kroemer G. TumGrowth: An open-access web tool for the statistical analysis of tumor growth curves. *Oncoimmunology.* 2018;7(9):e1462431.

Supplemental Figures

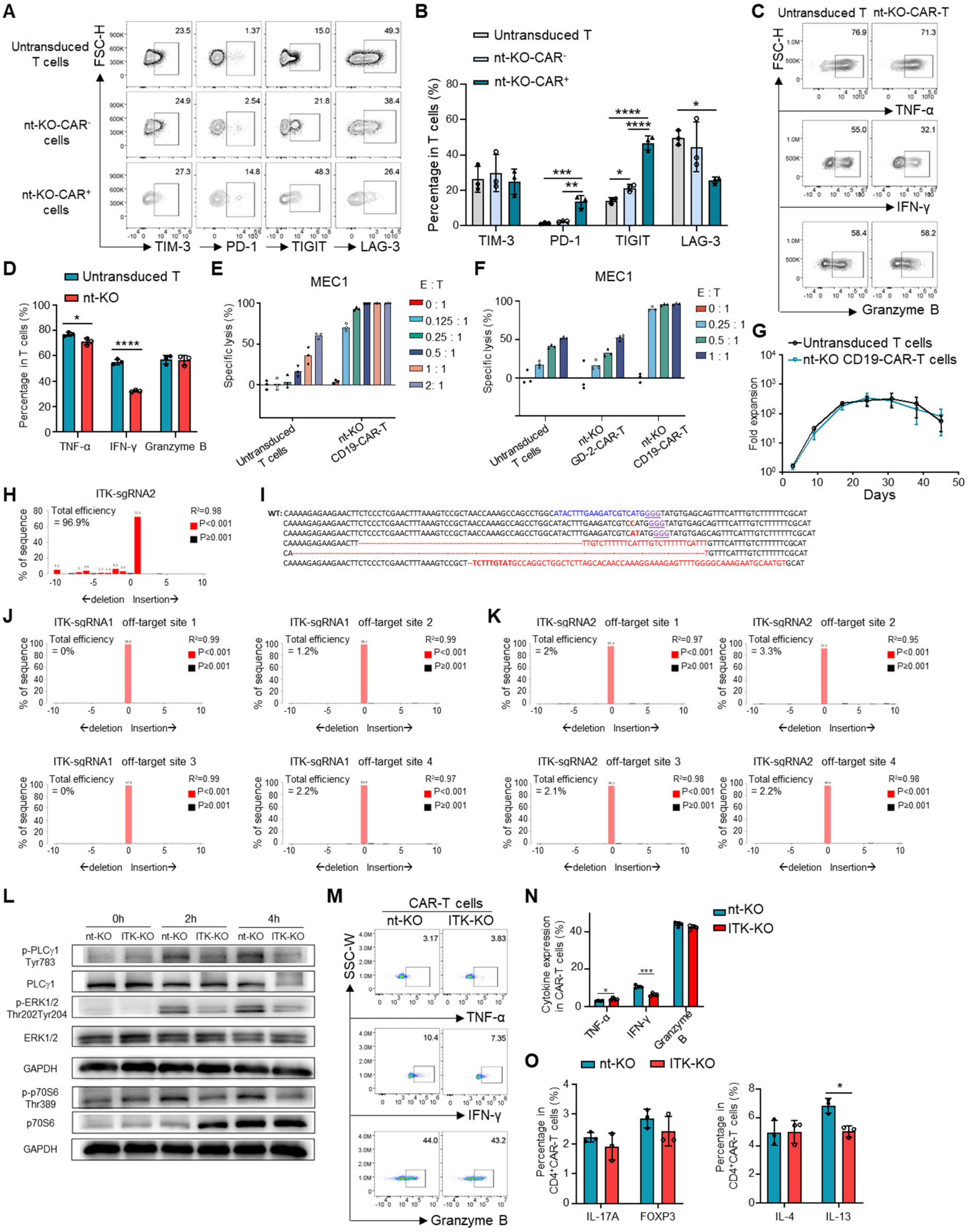

Supplemental Figure 1 ITK deficiency attenuates immediate cytotoxicity of CAR-T cells

Generation of CAR-T cells were described in **Figure 1B** and in the **Methods**. (**A**) Flow cytometry

analysis of expression of TIM-3, PD-1, TIGIT and LAG-3 in indicated T cells. Data were collected 72 hours after electroporation of RNP complex. (**B**) Summary of the percentages of TIM-3$^+$, PD-1$^+$, TIGIT$^+$ and LAG-3$^+$ cells as shown in **A** (n = 3). Statistical differences were determined by one-way ANOVA with Tukey correction for multiple comparisons (*, P < 0.05. **, P < 0.01. ***, P < 0.001. ****, P < 0.0001). (**C**) Flow cytometry analysis of TNF-α, IFN-γ and granzyme B expression in untransduced T cells and nt-KO CD19-CAR-T cells. Cells were stimulated with PMA and ionomycin for 2 hours, followed by the addition of Brefeldin A for an additional 2 hours. (**D**) Summary of the percentages of TNF-α, IFN-γ and granzyme B expression as shown in **C** (n = 3). (**E**) In vitro killing assay of untransduced T cells and nt-KO CD19-CAR-T cells against the MEC1 cells (n = 3). Luciferase-expressing MEC1 cells were mixed at the indicated ratios with indicated cells and analyzed 48 hours after co-culture. Effector (E): untransduced T cells or CAR-T cells. Target (T): MEC1 cells. (**F**) In vitro killing assay of untransduced T cells, nt-KO GD2-CAR-T cells and nt-KO CD19-CAR-T cells against the MEC1 cells (n = 3). (**G**) Fold expansion of untransduced T cells and nt-KO CD19-CAR-T cells at the indicated time points (n = 3). Fold expansion values were normalized to the average values of cell numbers at day 0. (**H**) Gene editing efficiency of *ITK* locus by sgRNA2 targeting *ITK* (ITK-sgRNA2). CAR-T cells were collected 3 days after electroporation of RNP complex for **H**-**K**. Data represent the results of three independent experiments. (**I**) Detection of gene editing at *ITK* locus by Sanger sequencing. PCR products of the *ITK* locus were cloned into pcDNA3.1 plasmids and then subjected to single-clone sequencing using Sanger sequencing. Blue, sgRNA sequence. Purple with underline, protospacer adjacent motif (PAM) sequence. Red, changed bases comparing to the wildtype sequence. (**J**) Detection of off-target effects of ITK-sgRNA1 at the predicted off-target DNA loci. (**K**) Detection of off-target effects of ITK-sgRNA2 at the predicted off-target DNA loci. (**L**) Representative figure of indicated protein expression by western blotting. Five days after electroporation of RNP complex, ITK-KO or nt-KO CD19-CAR-T cells were co-cultured with/without MEC1 cells for indicated times at the E:T ratio of 2:1. CAR-T cells were then enriched with T cell enrichment kit (Cat#17951,

STEMCELL Technologies) and processed to western blotting analysis. (**M**) Flow cytometry analysis of IFN-γ, TNF-α and Granzyme B expression in CAR-T cells. Raji cells stably expressing luciferase were used as the target cells and incubated with effector CAR-T cells at a 2:1 E:T ratio for 48 hours. (**N**) Statistical analysis of IFN-γ, TNF-α and Granzyme B expression showed in **M** (n = 4). (**O**) Statistical analysis of expression of indicated molecules in $CD4^{+}$CAR-T cells by flow cytometric analyses (n = 3). CAR-T cells were co-cultured with MEC1 cells for 48 hours. Compiled data from one independent experiment for **B**, **D**-**G**, **N** and **O**. Statistical differences were determined by two-tailed unpaired Student's *t*-test in **D**, **G**, **N** and **O**. Data represent results of at least two independent experiments. Data means, standard variations, statistical tests and indicators of significant tests were as defined in the main text for all supplemental figures.

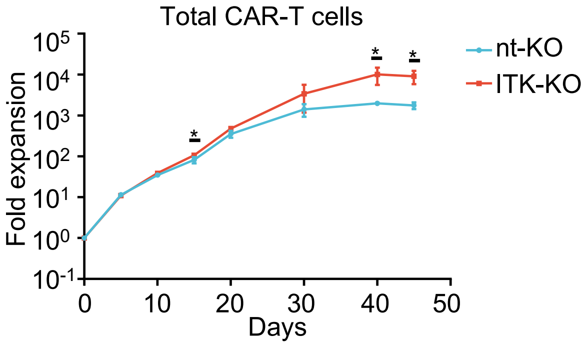

Supplemental Figure 2 ITK deficiency promotes long-term expansion of CD19-CAR-T cell in vitro

Fold expansion of the indicated CAR-T cells cultured in vitro (n = 3). Fold expansion values of the cell numbers were normalized to the average cell number of the CAR-T cells at day 0. Nt-KO or ITK-KO CD19-CAR-T cells were generated as described in the **Methods** and cultured for indicated days without co-culture with tumor cells. Compiled data from one independent experiment. Statistical differences were determined by two-tailed unpaired Student's *t*-test. Data represent results of two independent experiments.

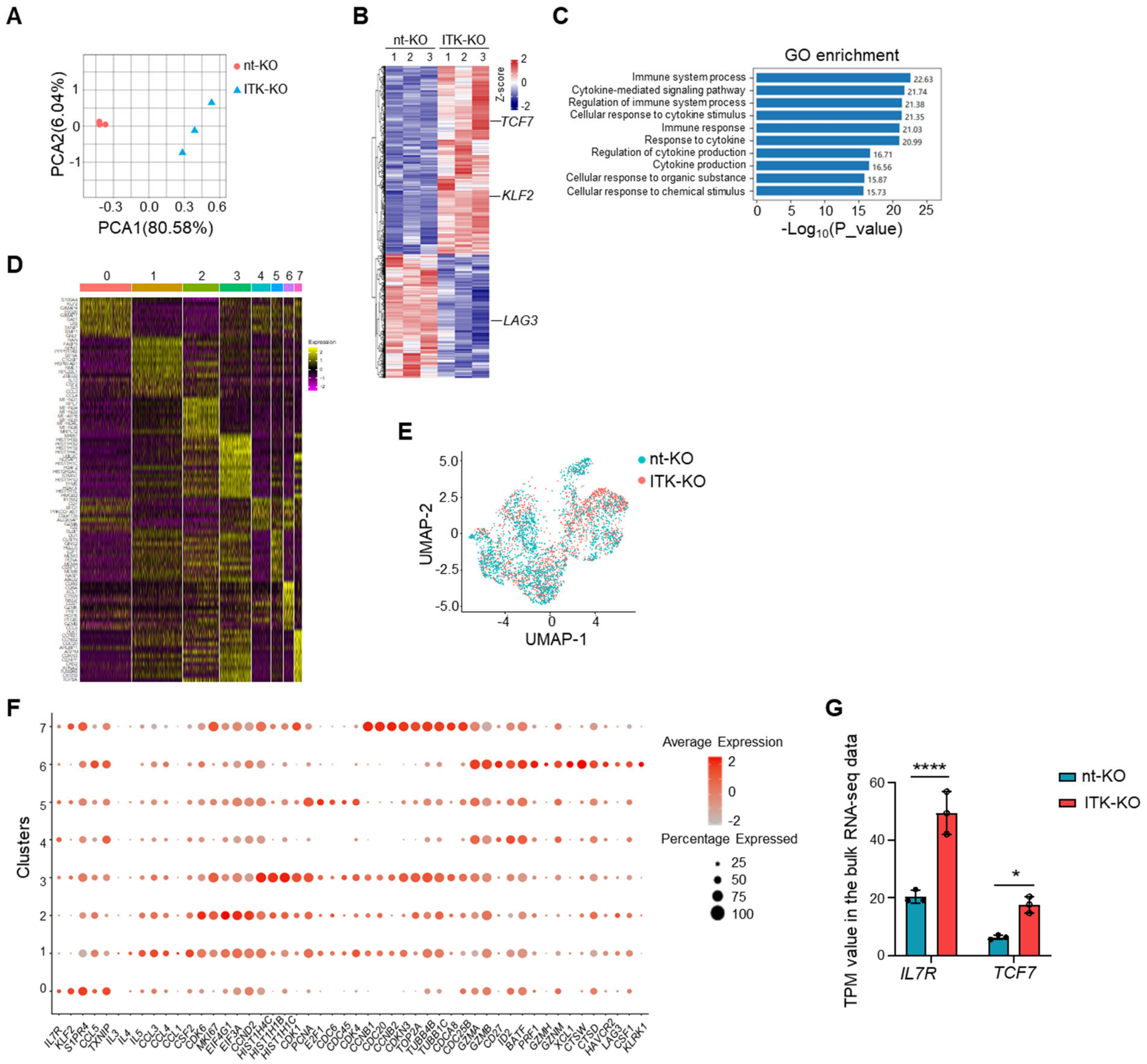

Supplemental Figure 3 Transcriptomic regulation of CD19-CAR-T cells by ITK

(**A**) Principal component analysis (PCA) of the bulk RNA-seq data of nt-KO and ITK-KO CD19-CAR-T cells. (**B**) Heatmap of differentially expressed genes, with *TCF7, KLF2* and *LAG3* highlighted, as identified by the bulk RNA-seq analysis. (**C**) Gene Ontology (GO) geneset enrichment analysis of differentially expressed genes between nt-KO and ITK-KO CD19-CAR-T cells using bulk RNA-seq data. (**D**) Expression of signature genes associated with clustering of CAR-T cells based scRNA-seq analysis. (E) Comparison of the UMAP-based scRNA-Seq data clustering profiles of nt-KO and ITK-KO CD19-CAR-T cells. (**F**) Expression of the indicated genes

in different CD19-CAR-T cell clusters identified by the scRNA-seq analysis. Clusters 2, 3, 5, and 7 show high expression of several genes involved in cell cycle. Cluster 2 shows elevated expression of *CDK6*, *E1F4G1*, *E1F3A* and *CCND2*. Cluster 3 exhibits higher levels of *HIST1H4C*, *HIST1H1B*, *CDK1* and *PCNA*. Cluster 5 shows elevated expression of *E2F1*, *CDC6*, *CDC45* and *CDK1*. Cluster 7 shows higher levels of cyclin proteins such as *CCNB1*, *CCNB2* and *CDC20*. (**G**) Transcript per million (TPM) values of *IL7R* and *TCF7* in nt-KO and ITK-KO CD19-CAR-T cells in the bulk RNA-seq data (n = 3). Statistical differences were determined by two-tailed unpaired Student's *t*-test.

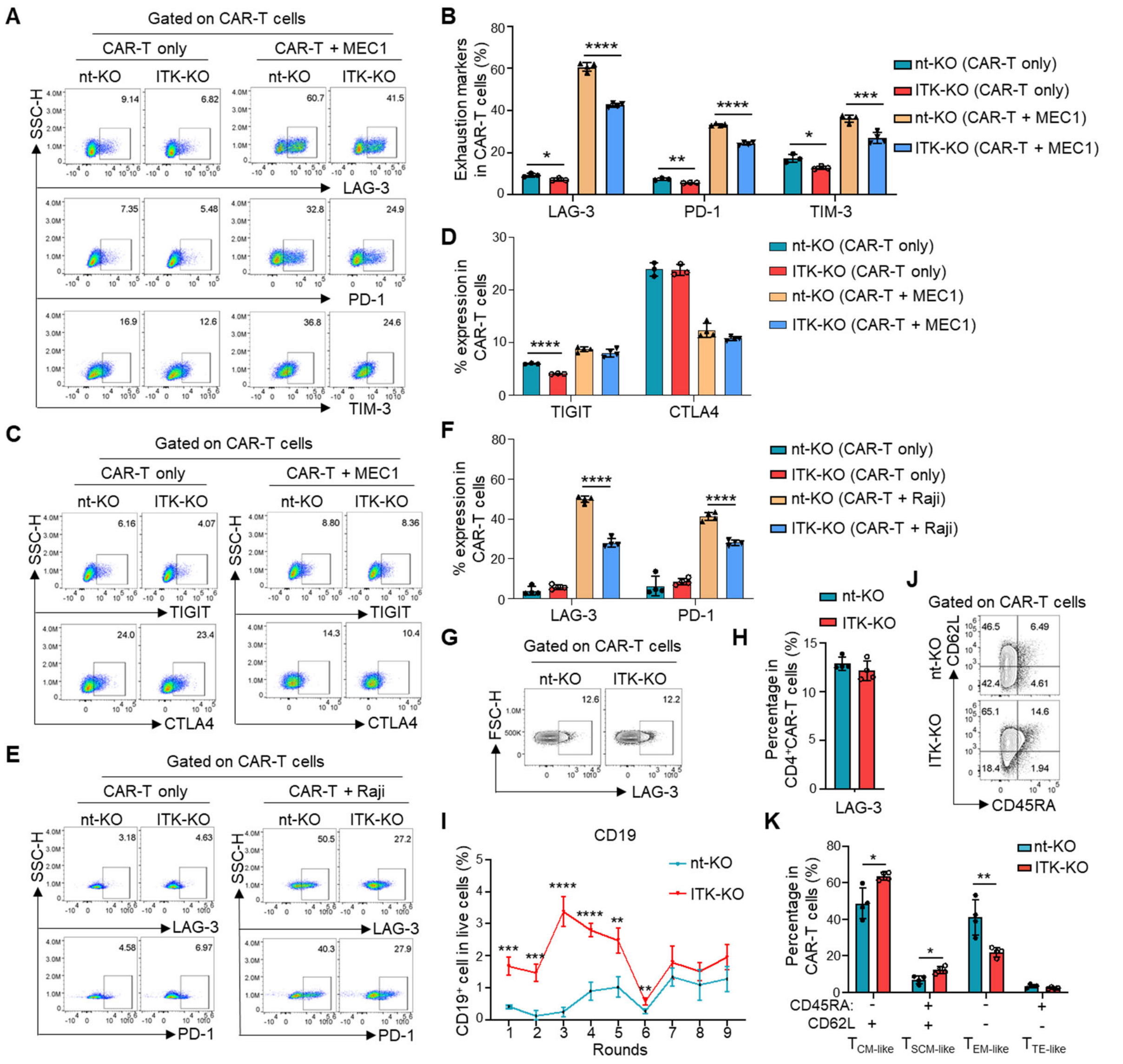

Supplemental Figure 4 ITK deficiency reduces exhaustion and promote memory phenotype in CD19-CAR-T cells in vitro

(**A**) Representative flow cytometric plots of LAG-3, PD-1 and TIM-3 expression in CAR-T cells after co-culture with MEC1 cells at the E:T ratio of 2:1 for 48 hours. (**B**) Summary of percentages of LAG-3⁺, PD-1⁺ and TIM-3⁺ cells as shown in **A** (n = 3 for each CAR-T only group, and n = 4 for each CAR-T + MEC1 group)**.** (**C**) Flow cytometric analyses of TIGIT and CTLA4 expression in indicated CAR-T cells after co-culture with or without MEC1 cells at the E:T ratio of 2:1 for 48 hours. (**D**) Statistical analysis of TIGIT and CTLA4 expression shown in **C** (n = 3 for each CAR-T

only group, n = 4 for each CAR-T + MEC1 group). (**E**) Flow cytometric analyses of LAG-3 and PD-1 expression in the indicated CAR-T cells after co-culture with or without Raji cells at the E:T ratio of 2:1 for 48 hours. (**F**) Statistical analysis of LAG-3 and PD-1 expression shown in **E** (n = 4). (**G**) Representative flow cytometric plots of LAG-3 expression in CAR-T cells at day 15 after co-cultured with MEC1 cells at the E:T ratio of 2:1. (**H**) Summary of percentages of LAG-3^{+} cells in **G** (n = 4)**.** (**I**) Summary of percentages of residual CD19^{+} cells in samples after indicated rounds of co-culture with MEC1 cells (n = 4). CAR-T cells were co-cultured with MEC1 cells at 2:1 ratio for 48 hours for each round. (**J**) Representative flow cytometric plots of CD62L and CD45RA expression in CAR-T cells at day 15 after co-cultured with MEC1 cells at the E:T ratio of 2:1. (**K**) Summary of percentages of indicated cell populations in **J** (n = 4)**.** Compiled data from one independent experiment for **B**, **D**, **F**, **H**, **I** and **K**. Statistical differences were determined by two-tailed unpaired Student's *t*-test. Data represented results of at least two independent experiments.

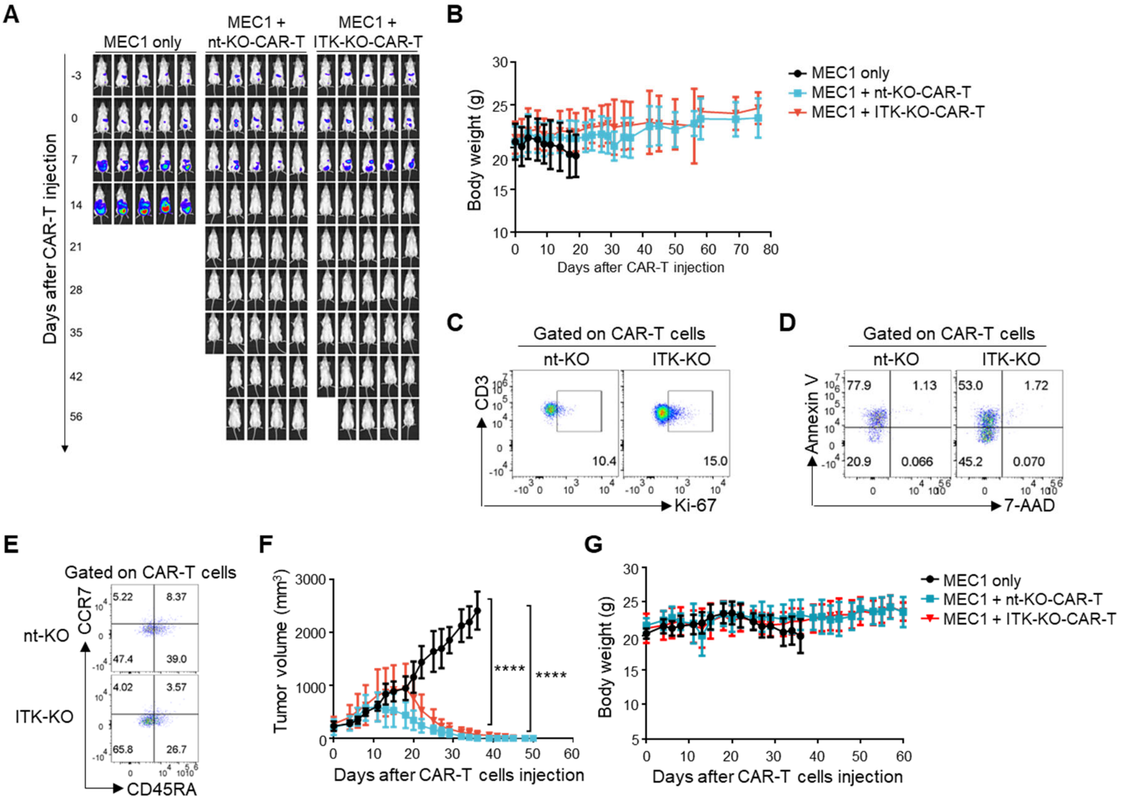

Supplemental Figure 5 ITK deficiency enhances expansion and long-term persistence of CD19-CAR-T cells in vivo

(**A**) Representative bioluminescence imaging of NPG mice xenografted with MEC1 cells stably expressing luciferase. Data are related to **Figure 5A**. (**B**) Body weight of groups of mice with indicated treatment shown in **A** (n = 4 for day 42, day 45, day 50, day 56, day 58, day 69, day 76 nt-KO groups and day 56 ITK-KO group. n=3 for day 58, day 69, day 76 ITK-KO groups. n = 5 for the rest of the groups). (**C**) Flow cytometric analyses of Ki-67 expression in CAR-T cells collected from the indicated recipients 11 days after CAR-T cell injection. (**D**) Representative flow cytometric plots of Annexin V and 7-AAD expression on CAR-T cells in the PB samples collected from nt-KO or ITK-KO CAR-T cell recipients 11 days after CAR-T cell injection. (**E**) Flow cytometric analyses of CCR7 and CD45RA expression in the indicated CAR-T cells collected. PBMCs were collected at day 33 after CAR-T cell injection. (**F**) Assessment of tumor volume of groups of mice receiving treatments as indicated in **Figure 5K** (n = 7 for day 27 MEC1 only group. n = 6 for day

29 and day 32 MEC1 only groups. n = 5 for day 34 MEC1 only group. n=4 for day 36 MEC1 only group. n = 5 for all nt-KO groups. n = 7 for all ITK-KO groups). Statistical analyses were conducted by linear mixed-effect modeling (over the whole-time course) (13) and Bonferroni correction for multiple comparisons was used. ****, P< 0.0001. (**G**) Body weight of groups of mice receiving treatments as indicated in **Figure 5K** (n = 7 for day 27 MEC1 only group. n = 6 for day 29 and day 32 MEC1 only groups. n = 5 for day 34 MEC1 only group. n=4 for day 36 MEC1 only group. n = 5 for all nt-KO groups. n = 7 for all ITK-KO groups). For comparison between two groups at each time point in **B** and **G**, two-tailed unpaired Student's *t*-test was used. For multiple comparisons among three groups at each time point in **B** and **G**, one-way ANOVA with Bonferroni correction for multiple comparisons was used. Compiled data from one independent experiment for **B**, **F** and **G**. Data represented results of two independent experiments.

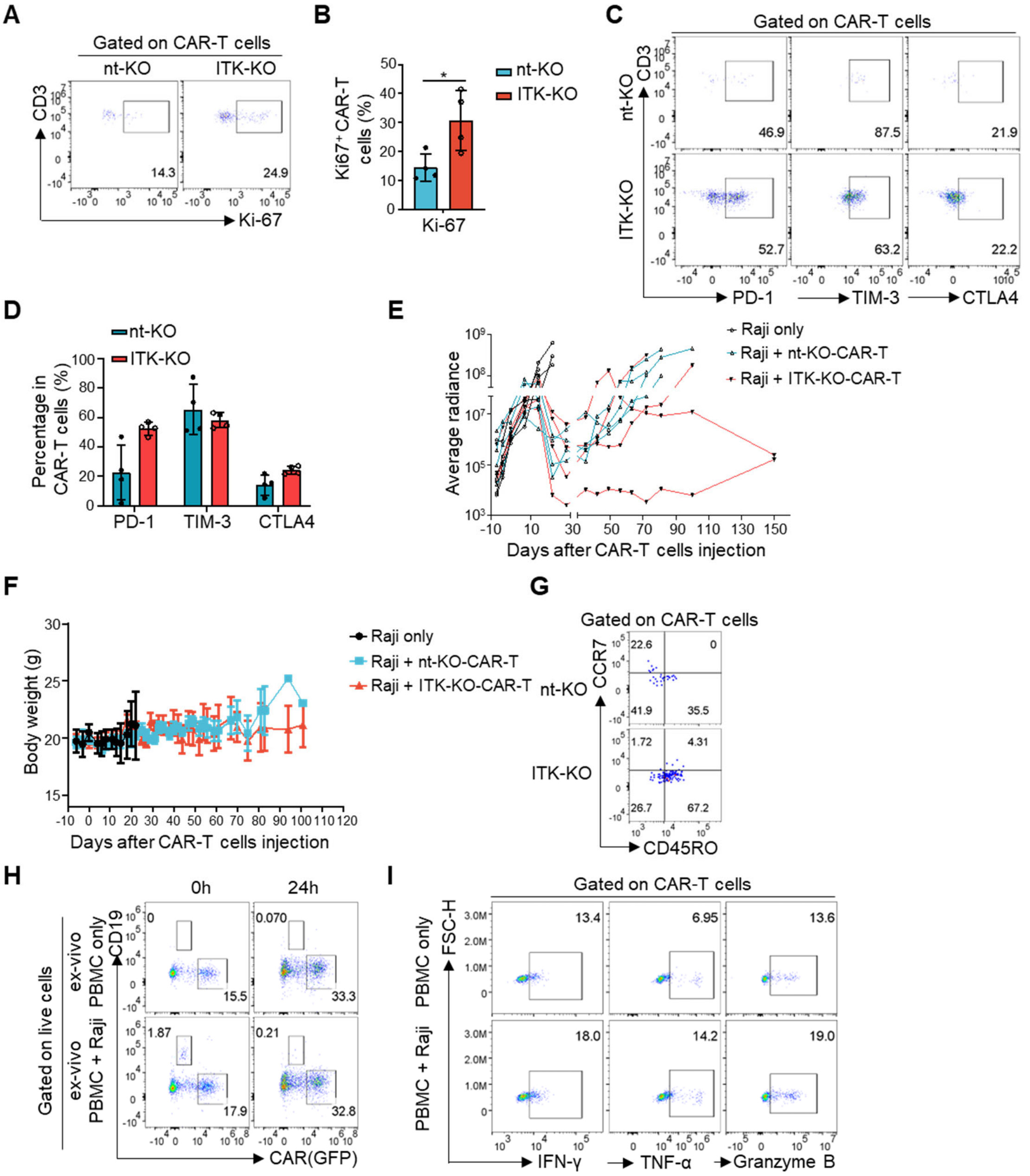

Supplemental Figure 6 ITK-deficient CAR-T cells significantly improve control of tumor relapse in vivo

(**A**) Flow cytometric plots of Ki-67 expression in nt-KO and ITK-KO CD19-CAR-T cells at Day 28 after CAR-T cell injection. (**B**) Statistical analysis of Ki-67⁺ CAR-T cells shown in **A** (n = 4). (**C**) Flow cytometric analyses of PD-1, TIM-3, and CTLA4 expression in CAR-T cells at day 24 after CAR-T cell injection. (**D**) Statistical analysis of PD-1, TIM-3, and CTLA4 expression in CD19-

CAR-T cells shown in **C** (n = 4). (**E**) Tumor burden in mice grafted with Raji cells stably expressing luciferase (Raji only) and treated with different CAR-T cells, as shown in **Figure 6H**. Tumor growth was monitored using bioluminescence imaging and plotted as average radiance (photons/s/cm2/sr) over time. (n = 3 for day 21 Raji only group, day 81 nt-KO group, day 81 and day 100 ITK-KO group; n = 1 for day nt-KO day 100 group; n = 2 for day 150 ITK-KO group; n = 4 for the rest of the groups). (**F**) Body weight of groups of mice shown in **Figure 6H** (n = 3 for day 20 and day 22 Raji only groups; n = 3 for day 81 and day 83 nt-KO groups; n = 1 for day 94 and day 101 nt-KO groups; n = 3 for day 75, day 81, day 83, day 94, and day 101 ITK-KO groups; n = 4 for the rest of the groups). For comparison between two groups at each time point in **F**, two-tailed unpaired Student's *t*-test was used. For multiple comparisons among three groups at each time point in **F**, one-way ANOVA with Bonferroni correction for multiple comparisons was used. (**G**) Flow cytometric plots of CCR7 and CD45RO expression in CAR-T cells collected from PBMC of mice 30 days post CAR-T cell injection. (**H**) Representative flow cytometric plots of CD19 and CAR-GFP expression in samples from ex-vivo PBMC co-cultured with/without Raji cells for 24 hours. PBMCs were collected 50 days after CAR-T cell injection. (**I**) Flow cytometric plots of IFN-γ, TNF-α, and Granzyme B expression in CAR-T cells cultured with or without Raji cells as shown in **H**. Compiled data from one experiment for **B** and **D**-**F**. Statistical differences were determined by two-tailed unpaired Student's *t*-test in **B** and **D**. Data represent results of two independent experiments.

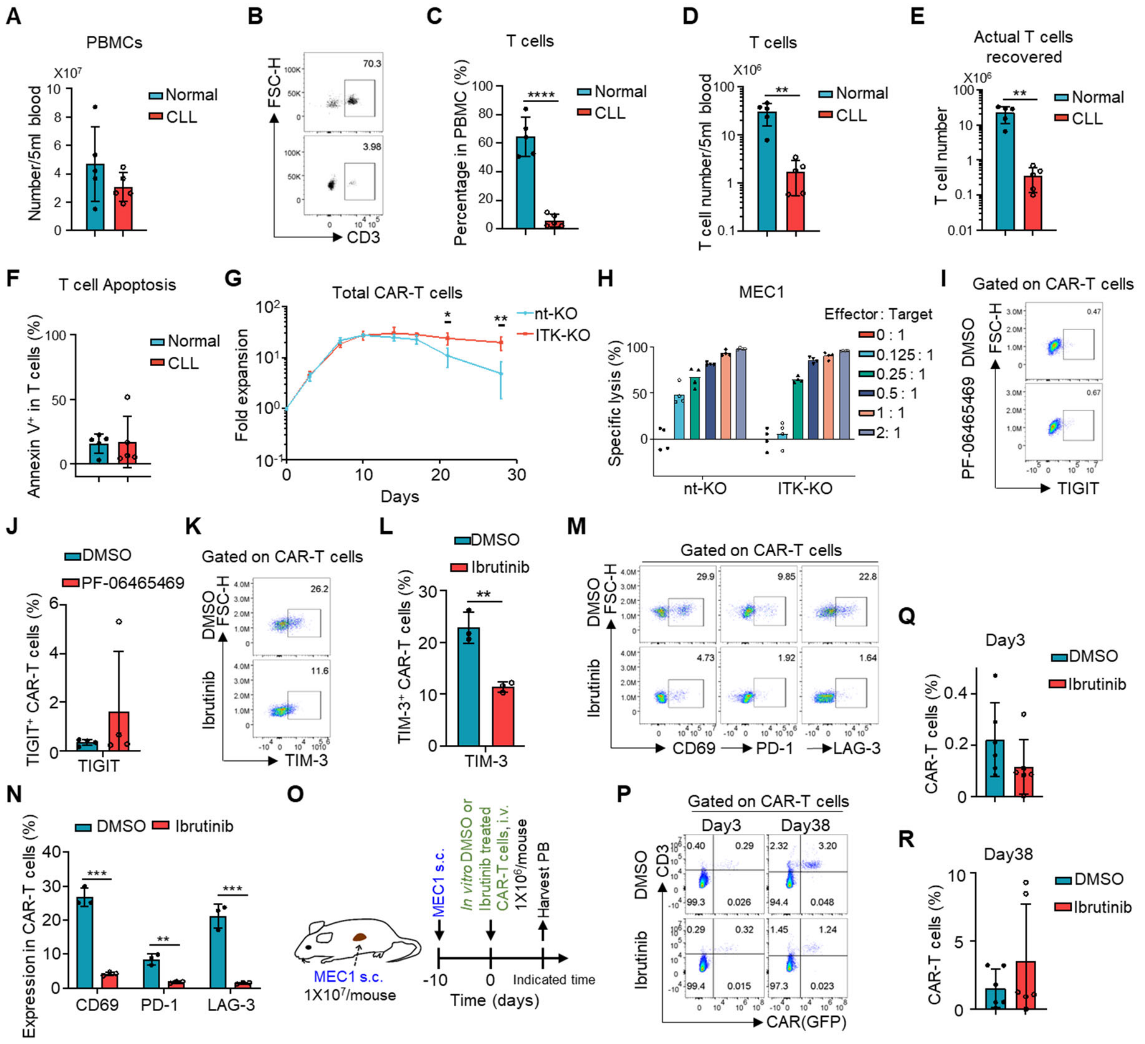

Supplemental Figure 7 ITK deficiency attenuates exhaustion and promotes memory phenotype in CD19-CAR-T cells derived from CLL patients

(**A**) Number of PBMCs in 5ml blood samples from CLL patients or normal donors (n=5). (**B**) Representative flowcytometry data of CD3 expression in PBMCs from CLL patients or normal donors. (**C**) Percentage of $CD3^{+}$T cells in PBMCs from CLL patients or normal donors (n=5). (**D**) Number of T cells per 5ml blood samples as calculated from data in panels **A** and **C** (n=5). (**E**) Actual T cells recovered from each PBMC samples (n=5). (**F**) Percentage of apoptosis in T cells from CLL patients or normal donors by flow cytometry analysis of Annexin V staining (n=5). (**G**) Fold expansion of the indicated CAR-T cells at the indicated time points, following 48 hours co-

culture with MEC1 cells at the E:T ratio of 2:1 (n = 4). Fold expansion values of the cell numbers were normalized to the average cell number of the CAR-T cells at day 0. (**H**) In vitro killing assay against the indicated MEC1 cells expressing luciferase using nt-KO and ITK-KO CLL-CD19-CAR-T cells at indicated E:T ratios for 48 hours (n = 4). (**I**) Flow cytometric plots of TIGIT expression in CLL-CAR-T cells co-cultured with MEC1 cells with or without PF-06465469 (1 μM) treatment. (**J**) Statistical analysis of the percentage of TIGIT$^+$ CAR-T cells in CLL-CD19-CAR-T cells shown in **I** (n = 4). (**K**) Flow cytometric plots of TIM-3 expression in CLL-CAR-T cells co-cultured with MEC1 cells with or without ibrutinib (0.78 μM) treatment. (**L**) Statistical analysis of the percentage of TIM-3$^+$ CAR-T cells in CLL-CD19-CAR-T cells shown in **K** (n = 3). (**M**) Flow cytometry analyses of CD69, LAG-3, and PD-1 expression in indicated CAR-T cells co-cultured with MEC1 cells with/without Ibrutinib treatment. (**N**) Statistical analysis of the percentage of CD69$^+$, LAG-3$^+$, and PD-1$^+$ cells in CD19-CAR-T cells shown in **M** (n = 3). (**O**) Experimental design of the MEC1-bearing NPG mouse model for CLL-CAR-T therapy. MEC1 cells stably expressing luciferase were subcutaneously injected into 6-8 weeks old NPG mice. Control (DMSO) or ibrutinib-treated CLL-CAR-T cells were intravenously injected into recipients 10 days after the MEC1 injection. Peripheral blood (PB) cells were collected at different time points after the CAR-T cell injection. (**P**) Flow cytometric plots of CD3 and CAR-GFP expression in PB cells collected from **O** at indicated time points. (**Q** and **R**) Statistical analysis of the percentage of CD3$^+$ GFP$^+$ CAR-T cells in PBMCs at day 3 (**Q**) and day 38 (**R**) from samples analyzed in **P** (n = 6). Compiled data from one experiment for **A**, **C-H**, **J**, **L**, **N**, **Q** and **R**. Statistical differences were determined by two-tailed unpaired Student's *t*-test in **A**, **C-G**, **J**, **L**, **N**, **Q** and **R**. Data represent results of at least two independent experiments.

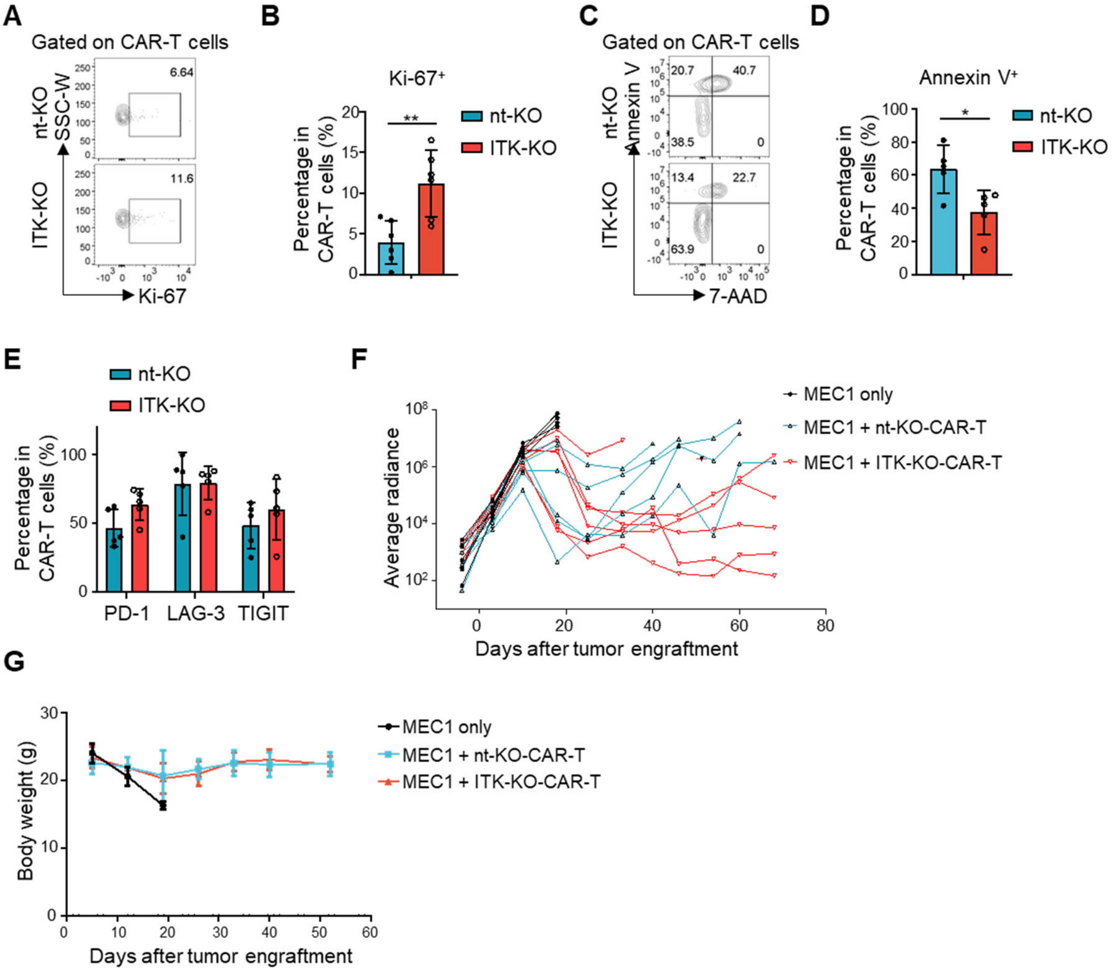

Supplemental Figure 8 ITK-deficient CAR-T cells derived from CLL patients improve control of tumor relapse in vivo.

(**A**) Representative flow cytometric plots of Ki-67 expression on CAR-T cells in the PBMCs collected from nt-KO or ITK-KO CLL-CD19-CAR-T recipients 23 days post-infusion. Experiments were performed as described in **Figure 8A**. (**B**) Summary of the percentages of CAR-T cells that are Ki-67$^+$ as shown in **A** (n=5). (**C**) Representative flow cytometric plots of Annexin V and 7-AAD expression on nt-KO or ITK-KO CLL-CD19-CAR-T cells at day 14 after CAR-T injection. (**D**) Summary of the percentages of CAR-T cells that are Annexin V$^+$ as shown in **C** (n=5). (**E**) Summary of the percentages of CAR-T cells that are PD-1$^+$, LAG-3$^+$ and TIGIT$^+$ by flow cytometry analysis at day 26 after CAR-T injection (n=5). (**F**) Tumor burden in mice grafted with MEC1 cells

expressing luciferase (MEC1 only) and treated with different CAR-T cells, as shown in **Figure 8H**. Tumor growth was monitored using bioluminescence imaging and plotted as average radiance (photons/s/cm2/sr) over time (n = 5 for the MEC1 only group on day 18, nt-KO groups on day 25, 33, and 40, as well as the ITK-KO groups on day 40, 54, 60 and 68. n=4 for nt-KO group on day 46. n=3 for nt-KO groups on day 54 and 60. n=1 for nt-KO group on day 68. n = 6 for the rest groups). (**G**) Body weight of groups of mice as shown in **Figure 8H** (n = 5 for the MEC1 only group on day 19, nt-KO groups on day 26, 33, and 40, as well as the ITK-KO groups on day 40 and 52. n=4 for nt-KO group on day 52. n = 6 for the rest groups). For comparison between two groups at each time point in **G**, two-tailed unpaired Student's *t*-test was used. For multiple comparisons among three groups at each time point in **G**, one-way ANOVA with Bonferroni correction for multiple comparisons was performed. Compiled data from two independent experiments in **B**, **D** and **E**-**G**. Statistical differences were determined by two-tailed unpaired Student's *t*-test in **B**, **D** and **E**. Representative data from two independent experiments.

Supplemental Table 1 Sequences of the sgRNAs and primers for validation of gene editing efficiency

**sgRNA sequence (5' - 3')**	**Primer name**	**Primer sequence (5' - 3')**
ITK-sg1: ATACTTTGAAGATCGTCATG	ITK-sg1_F<br>ITK-sg1_R	GCTAAGAGGTGATGCCCAAG<br>GGGGTAGATCATCCCATTGA
ITK-sg2: ATCCTCAGGAACTCGCACTG	ITK-sg2_F<br>ITK-sg2_R	CTGTAACTCTAACTCATAA<br>AATAAGCACTTCCTAATGG
nt-sg1: ACGGAGGCTAAGCGTCGCAA	-<br>-	-<br>-

Note: F – forward primer; R – reverse primer.

Supplemental Table 2 Predicted off-target sequences and primers for validation of off-target efficiency

**Predicted off-target sequence (5' - 3')**	**Primer name**	**Primer sequence (5' - 3')**
ITK-sg1-off1:	ITK-sg1-off1_F	TGATTTTTGGTATCTCCCTGTG
AAACTTTAAAAATCATCATGTGG	ITK-sg1-off1_R	TCAGTTCCCACTTAGCAGAGC
ITK-sg1-off2:	ITK-sg1-off2_F	CATGGACTGCTGTGATCCTA
GTAATTTGAAAATCATCATGGGG	ITK-sg1-off2_R	TGGTTGTATTCTGGGCTGGT
ITK-sg1-off3:	ITK-sg1-off3_F	GGGGAATCATCCTCCAAGTT
ATAATATGAAAATCGACATGAGG	ITK-sg1-off3_R	TCACGTGATCCTCCCTCTTC
ITK-sg1-off4:	ITK-sg1-off4_F	GCAAGAATTTCAACGGGTAGAG
ATAATATGAAAATCGACATGAGG	ITK-sg1-off4_R	GAAAACTTCCAGCCAGGTACA
ITK-sg2-off1:	ITK-sg2-off1_F	TCCCAGCTGAGGCAACTAAG
CGCCCCAGGAACTCACACTGTGG	ITK-sg2-off1_R	ATCTCTCGGTGCATGGAGTC
ITK-sg2-off2:	ITK-sg2-off2_F	TCCTGTTTCAAGAGCCCAAC
AGCCCCAGGAACCCACACTGGGG	ITK-sg2-off2_R	ACCCAGCCTTCTGACAACAC
ITK-sg2-off3:	ITK-sg2-off3_F	GTGAAGGCCCTTTGTGAAAA
GTCCTTAGGAACTCACACTTGGG	ITK-sg2-off3_R	TTTTGGTGACATACCAATCCTG
ITK-sg2-off4:	ITK-sg2-off4_F	TCTCAGACTTCACGTGGGAAT
ACCAGCAGGAACTCGCACTAAGG	ITK-sg2-off4_R	GAGCACGATGCTGTAATGGA

Supplemental Table 3 Top100 expressed genes in each cluster in the single-cell RNA-seq.

Clusters	Gene Name	Gene Rank	P_value	avg_log2FC	adj-P_value (Dunn-Bonferroni correction)
0	*GNLY*	1	6.83E-15	1.715636773	2.50E-10
0	*LTB*	2	5.69E-109	1.603656001	2.08E-104
0	*KLF2*	3	2.55E-151	1.503597027	9.34E-147
0	*S100A4*	4	5.03E-190	1.383877126	1.84E-185
0	*TXNIP*	5	6.46E-94	1.35828689	2.37E-89
0	*CD52*	6	1.11E-172	1.251645134	4.07E-168
0	*ISG20*	7	1.25E-124	1.24411446	4.58E-120
0	*B2M*	8	8.54E-259	1.180392024	3.13E-254
0	*TIMP1*	9	6.02E-87	1.166844254	2.20E-82
0	*GIMAP4*	10	2.14E-125	1.15404953	7.82E-121
0	*RPS4Y1*	11	3.15E-106	1.123219301	1.15E-101
0	*IL32*	12	1.08E-176	1.083264808	3.94E-172
0	*IFI27*	13	1.12E-14	1.070411833	4.11E-10
0	*S100A6*	14	6.37E-182	1.066676988	2.33E-177
0	*SAT1*	15	1.36E-111	1.056622329	4.99E-107
0	*GIMAP7*	16	7.86E-114	1.040769002	2.88E-109
0	*PLAAT4*	17	6.34E-71	1.035040743	2.32E-66
0	*ISG15*	18	8.26E-129	1.017182304	3.02E-124
0	*PCED1B-AS1*	19	1.96E-67	1.006016272	7.17E-63
0	*CYBA*	20	2.28E-207	1.000951318	8.33E-203
0	*ALOX5AP*	21	1.68E-89	0.993824355	6.14E-85
0	*MAL*	22	3.95E-58	0.985002188	1.45E-53
0	*ODAPH*	23	1.31E-60	0.982477626	4.80E-56
0	*NPC2*	24	2.33E-84	0.970331478	8.54E-80
0	*TNFRSF4*	25	1.35E-53	0.938302053	4.93E-49
0	*BTG1*	26	1.11E-97	0.923430815	4.08E-93
0	*S100P*	27	1.12E-46	0.916077133	4.10E-42
0	*S1PR4*	28	2.03E-100	0.912672172	7.42E-96
0	*CCL5*	29	2.02E-08	0.89790199	0.00074092
0	*MAF*	30	5.22E-62	0.865050313	1.91E-57
0	*HLA-A*	31	1.01E-194	0.862292655	3.70E-190
0	*RNASEK*	32	1.76E-143	0.860299986	6.45E-139
0	*TYMP*	33	5.49E-80	0.860233042	2.01E-75
0	*HLA-C*	34	1.19E-174	0.843986502	4.36E-170
0	*HLA-DPB1*	35	9.41E-81	0.839481634	3.44E-76

0	*HLA-DRB1*	36	5.31E-49	0.834757104	1.94E-44
0	*HPGD*	37	1.23E-10	0.820293529	4.50E-06
0	*ITM2C*	38	3.31E-37	0.817855678	1.21E-32
0	*NEAT1*	39	3.01E-44	0.81226139	1.10E-39
0	*IL17RB*	40	1.52E-28	0.810996813	5.56E-24
0	*GSTK1*	41	5.87E-102	0.80520278	2.15E-97
0	*JUNB*	42	3.18E-79	0.805087707	1.16E-74
0	*IL7R*	43	5.71E-15	0.804901621	2.09E-10
0	*RABAC1*	44	4.35E-108	0.803722898	1.59E-103
0	*GIMAP1*	45	1.09E-74	0.801809253	3.98E-70
0	*CD37*	46	1.36E-89	0.799011646	4.96E-85
0	*FTH1*	47	8.57E-85	0.798953019	3.14E-80
0	*CST7*	48	1.47E-47	0.792856096	5.37E-43
0	*IL3RA*	49	2.31E-31	0.787021474	8.47E-27
0	*EPSTI1*	50	5.84E-79	0.785378833	2.14E-74
0	*S100A11*	51	5.19E-132	0.774019515	1.90E-127
0	*EMP3*	52	8.16E-113	0.773203313	2.99E-108
0	*PBXIP1*	53	2.12E-73	0.764034253	7.78E-69
0	*ATP6V0E1*	54	8.56E-113	0.763302994	3.13E-108
0	*FXYD5*	55	1.84E-101	0.757097013	6.73E-97
0	*ARL6IP5*	56	1.98E-113	0.751253586	7.24E-109
0	*TRADD*	57	5.01E-79	0.747548302	1.84E-74
0	*ITM2B*	58	9.24E-100	0.747546035	3.38E-95
0	*RNF213*	59	6.99E-97	0.744573085	2.56E-92
0	*LIME1*	60	2.80E-37	0.744535524	1.02E-32
0	*HLA-B*	61	1.96E-173	0.740849121	7.16E-169
0	*YPEL3*	62	4.25E-76	0.738364127	1.55E-71
0	*SELPLG*	63	1.20E-75	0.737260392	4.41E-71
0	*SOCS3*	64	1.07E-53	0.720930303	3.92E-49
0	*CD3D*	65	7.04E-135	0.720284726	2.58E-130
0	*IFI6*	66	1.73E-52	0.710975305	6.32E-48
0	*CXCR4*	67	4.05E-37	0.706459737	1.48E-32
0	*BST2*	68	5.61E-90	0.706241216	2.05E-85
0	*IL2RG*	69	1.26E-97	0.705676153	4.63E-93
0	*TMEM50A*	70	1.21E-87	0.690547756	4.44E-83
0	*EGLN3*	71	2.39E-16	0.686019299	8.74E-12
0	*MALAT1*	72	1.43E-34	0.684293218	5.24E-30
0	*RNASET2*	73	4.31E-46	0.683983827	1.58E-41
0	*LIMD2*	74	1.07E-84	0.682990359	3.93E-80

0	*HLA-E*	75	4.80E-107	0.682024622	1.76E-102
0	*ETV7*	76	5.57E-47	0.676829155	2.04E-42
0	*IL10RA*	77	2.79E-57	0.67671467	1.02E-52
0	*GABARAP*	78	1.78E-81	0.675966702	6.51E-77
0	*CSTB*	79	7.22E-88	0.669661054	2.64E-83
0	*ARL4C*	80	4.89E-69	0.665702074	1.79E-64
0	*OPTN*	81	1.41E-72	0.665611437	5.16E-68
0	*RIPOR2*	82	5.63E-48	0.662076017	2.06E-43
0	*FTL*	83	3.23E-75	0.660211979	1.18E-70
0	*GADD45G*	84	4.22E-21	0.656455389	1.54E-16
0	*ATP6V1G1*	85	3.66E-74	0.652646739	1.34E-69
0	*SLFN5*	86	7.03E-55	0.650769877	2.57E-50
0	*C4orf3*	87	1.90E-77	0.649267451	6.97E-73
0	*OSTF1*	88	5.24E-58	0.644299079	1.92E-53
0	*BRI3*	89	5.04E-46	0.641454413	1.84E-41
0	*POLB*	90	8.68E-19	0.639983315	3.18E-14
0	*HSPA1A*	91	1.82E-46	0.63700814	6.67E-42
0	*HLA-F*	92	1.81E-52	0.636711376	6.64E-48
0	*GIMAP5*	93	9.67E-50	0.636353071	3.54E-45
0	*UQCRB*	94	5.60E-79	0.629498963	2.05E-74
0	*MXD4*	95	2.50E-56	0.625180452	9.14E-52
0	*TMSB4X*	96	1.02E-125	0.624519519	3.75E-121
0	*LGALS1*	97	6.34E-50	0.609533244	2.32E-45
0	*LGALS3*	98	3.28E-20	0.608138258	1.20E-15
0	*SQSTM1*	99	2.90E-34	0.60619486	1.06E-29
0	*TMEM59*	100	3.20E-56	0.60518429	1.17E-51
1	*CCL3*	101	8.24E-44	1.869057401	3.02E-39
1	*CCL4*	102	7.60E-13	1.737777052	2.78E-08
1	*FABP5*	103	1.50E-246	1.696627486	5.49E-242
1	*CCL1*	104	1.40E-22	1.615510324	5.13E-18
1	*IL5*	105	2.85E-60	1.606442342	1.04E-55
1	*CSF2*	106	9.76E-74	1.605622533	3.57E-69
1	*C1QBP*	107	4.66E-211	1.234682359	1.71E-206
1	*PPP1R14B*	108	1.63E-240	1.170735445	5.95E-236
1	*NPM1*	109	4.67E-243	1.152957556	1.71E-238
1	*IL13*	110	1.61E-125	1.13600379	5.88E-121
1	*RAN*	111	2.52E-268	1.130912466	9.21E-264
1	*RPL22L1*	112	6.82E-172	1.101950459	2.50E-167
1	*ZBED2*	113	7.06E-156	1.051797308	2.58E-151

1	*IL3*	114	8.74E-14	1.045931983	3.20E-09
1	*EIF5A*	115	7.18E-227	1.045422711	2.63E-222
1	*HSP90AB1*	116	4.08E-187	1.023217757	1.49E-182
1	*NME1*	117	3.05E-174	1.005510389	1.12E-169
1	*HSPD1*	118	1.47E-169	0.990755928	5.38E-165
1	*HSPE1*	119	8.53E-164	0.970381536	3.12E-159
1	*PGAM1*	120	2.01E-208	0.961196601	7.35E-204
1	*IL2RA*	121	2.89E-135	0.954136018	1.06E-130
1	*PDCD5*	122	6.91E-182	0.941769036	2.53E-177
1	*RANBP1*	123	8.36E-177	0.933181456	3.06E-172
1	*LIF*	124	2.77E-107	0.922240624	1.01E-102
1	*SLIRP*	125	4.89E-169	0.919050517	1.79E-164
1	*RPS17*	126	2.24E-206	0.918562229	8.18E-202
1	*MT2A*	127	5.74E-87	0.916204091	2.10E-82
1	*LINC00892*	128	5.41E-134	0.884354109	1.98E-129
1	*CCT2*	129	3.90E-160	0.852927409	1.43E-155
1	*ODC1*	130	2.20E-139	0.850662021	8.03E-135
1	*MRTO4*	131	2.59E-171	0.843561816	9.47E-167
1	*NME2*	132	7.34E-195	0.829125201	2.69E-190
1	*DDX21*	133	1.20E-133	0.82384868	4.37E-129
1	*IL4*	134	7.25E-33	0.821394193	2.65E-28
1	*IER3*	135	1.26E-76	0.820535938	4.60E-72
1	*LTA*	136	4.49E-61	0.805905288	1.64E-56
1	*PPA1*	137	2.38E-138	0.804780322	8.70E-134
1	*TOMM6*	138	8.48E-160	0.803811101	3.10E-155
1	*DCTPP1*	139	3.12E-155	0.803183042	1.14E-150
1	*SRM*	140	4.49E-115	0.795669311	1.64E-110
1	*TOMM5*	141	3.91E-141	0.787490336	1.43E-136
1	*ATP5MC3*	142	1.44E-186	0.787075465	5.27E-182
1	*EEF1E1*	143	8.35E-165	0.78249799	3.06E-160
1	*LDHA*	144	1.80E-145	0.777292379	6.57E-141
1	*PRDX1*	145	2.54E-164	0.775474804	9.31E-160
1	*SNRPF*	146	2.83E-169	0.771823487	1.04E-164
1	*RPL5*	147	6.91E-193	0.765211807	2.53E-188
1	*CCT6A*	148	2.97E-141	0.764873699	1.09E-136
1	*NCL*	149	1.84E-110	0.756354851	6.74E-106
1	*CCT5*	150	1.46E-136	0.753718134	5.35E-132
1	*DUSP4*	151	1.49E-120	0.744431879	5.44E-116
1	*SRSF7*	152	5.99E-133	0.727597014	2.19E-128

1	*LMNA*	153	3.89E-73	0.725181262	1.42E-68
1	*SNRPD1*	154	1.46E-144	0.724489901	5.33E-140
1	*SEC11C*	155	8.25E-113	0.722338089	3.02E-108
1	*EIF4A1*	156	2.84E-158	0.711235681	1.04E-153
1	*TOMM40*	157	1.71E-126	0.71007612	6.25E-122
1	*NDUFAB1*	158	2.98E-135	0.7020757	1.09E-130
1	*NHP2*	159	2.18E-125	0.699861668	8.00E-121
1	*MIR155HG*	160	8.88E-127	0.696665244	3.25E-122
1	*VDAC1*	161	1.41E-130	0.695798985	5.17E-126
1	*PHB*	162	1.15E-126	0.695714697	4.20E-122
1	*SNRPE*	163	2.26E-124	0.689829138	8.26E-120
1	*SERBP1*	164	1.10E-137	0.688743541	4.01E-133
1	*PSMB5*	165	3.95E-122	0.679728003	1.45E-117
1	*CCT7*	166	1.54E-122	0.674346045	5.62E-118
1	*SNRPB*	167	2.61E-143	0.672273209	9.55E-139
1	*RPS26*	168	1.74E-182	0.668740438	6.36E-178
1	*ATP5MC1*	169	1.29E-116	0.664566166	4.74E-112
1	*SSBP1*	170	2.43E-123	0.662228778	8.90E-119
1	*PSMA2*	171	1.53E-123	0.659009421	5.62E-119
1	*LSM5*	172	1.23E-117	0.657371142	4.49E-113
1	*PRMT1*	173	5.02E-104	0.655283287	1.84E-99
1	*EBNA1BP2*	174	4.14E-119	0.653791494	1.52E-114
1	*CCT3*	175	8.03E-114	0.647478173	2.94E-109
1	*SRI*	176	3.03E-112	0.637777263	1.11E-107
1	*CAVIN3*	177	1.95E-86	0.637032511	7.15E-82
1	*EEF1B2*	178	1.39E-128	0.636867476	5.07E-124
1	*YBX1*	179	1.07E-162	0.636586574	3.93E-158
1	*EIF1AX*	180	1.61E-109	0.635814718	5.89E-105
1	*GTPBP4*	181	6.72E-112	0.630329798	2.46E-107
1	*NOL7*	182	1.40E-114	0.623384904	5.11E-110
1	*HSP90AA1*	183	1.36E-115	0.623029908	4.98E-111
1	*SNRPA1*	184	3.74E-94	0.621761174	1.37E-89
1	*PRELID1*	185	2.17E-122	0.621153141	7.96E-118
1	*PTGES3*	186	8.40E-103	0.620747662	3.07E-98
1	*MYC*	187	1.39E-100	0.619990988	5.08E-96
1	*CD70*	188	7.20E-71	0.619459695	2.63E-66
1	*NOP16*	189	3.64E-142	0.618620291	1.33E-137
1	*PSMA7*	190	2.91E-149	0.617615575	1.07E-144
1	*IMPDH2*	191	1.51E-107	0.615023349	5.53E-103

1	*NDUFAF8*	192	2.66E-102	0.611796845	9.74E-98
1	*MRPL3*	193	1.77E-116	0.610645349	6.50E-112
1	*TIMM13*	194	1.95E-92	0.607493559	7.15E-88
1	*RPL8*	195	4.76E-182	0.607301262	1.74E-177
1	*CYCS*	196	8.49E-97	0.60625445	3.11E-92
1	*BTF3*	197	2.52E-123	0.606242376	9.21E-119
1	*SNHG8*	198	1.44E-79	0.59710231	5.26E-75
1	*BANF1*	199	1.99E-104	0.595008065	7.28E-100
1	*LYAR*	200	1.19E-99	0.5944799	4.36E-95
2	*MT-ND2*	201	4.87E-215	2.028298935	1.78E-210
2	*MT-ND4*	202	6.53E-224	1.977250428	2.39E-219
2	*MT-ND1*	203	1.28E-232	1.901886725	4.67E-228
2	*MT-ATP6*	204	4.00E-208	1.8487545	1.47E-203
2	*MT-ND5*	205	1.92E-191	1.811541819	7.04E-187
2	*MT-CO2*	206	5.76E-248	1.762578631	2.11E-243
2	*MT-CO3*	207	5.95E-228	1.755691461	2.18E-223
2	*MT-ND3*	208	2.23E-226	1.660843871	8.18E-222
2	*MT-ND4L*	209	3.29E-164	1.654197196	1.20E-159
2	*MT-CYB*	210	2.20E-206	1.635715791	8.04E-202
2	*RPL7*	211	6.17E-232	1.629916995	2.26E-227
2	*MT-CO1*	212	1.45E-229	1.609949822	5.30E-225
2	*MT-ND6*	213	1.04E-138	1.531736427	3.80E-134
2	*MRPL12*	214	5.71E-132	1.364966587	2.09E-127
2	*MKI67*	215	5.85E-109	1.361824556	2.14E-104
2	*NOP56*	216	8.76E-132	1.351588498	3.21E-127
2	*ASPM*	217	2.92E-82	1.211825567	1.07E-77
2	*RABL6*	218	1.15E-129	1.176067144	4.20E-125
2	*MT-ATP8*	219	5.87E-102	1.173704246	2.15E-97
2	*HSPA8*	220	1.68E-156	1.17136383	6.15E-152
2	*CDK6*	221	7.85E-108	1.161792261	2.87E-103
2	*PTPN7*	222	3.07E-143	1.115778937	1.12E-138
2	*AHI1*	223	3.88E-66	1.09835754	1.42E-61
2	*NSD2*	224	5.12E-109	1.094861485	1.88E-104
2	*EIF4G1*	225	1.53E-120	1.090350685	5.59E-116
2	*KPNB1*	226	5.13E-127	1.083854202	1.88E-122
2	*EHBP1L1*	227	3.38E-98	1.063287295	1.24E-93
2	*LONP1*	228	7.98E-112	1.059462858	2.92E-107
2	*HSP90AB1*	229	4.49E-101	1.05714454	1.64E-96
2	*MBD3*	230	7.33E-109	1.054158697	2.68E-104

2	*TOP2A*	231	8.77E-63	1.03807871	3.21E-58
2	*TCOF1*	232	1.32E-101	1.027407157	4.83E-97
2	*RIF1*	233	8.53E-97	1.025253755	3.12E-92
2	*CENPF*	234	3.52E-57	1.022342658	1.29E-52
2	*CCDC88B*	235	2.00E-93	1.019114899	7.31E-89
2	*PKM*	236	1.22E-153	0.997353036	4.45E-149
2	*BCLAF1*	237	3.18E-90	0.995102768	1.16E-85
2	*EIF3B*	238	6.72E-92	0.992335723	2.46E-87
2	*RRM2*	239	5.12E-78	0.970820621	1.87E-73
2	*ZAP70*	240	4.92E-76	0.968125596	1.80E-71
2	*HNRNPH1*	241	3.85E-94	0.967308724	1.41E-89
2	*FBL*	242	3.14E-78	0.961065877	1.15E-73
2	*UQCRC1*	243	6.35E-90	0.95539934	2.32E-85
2	*TRIM28*	244	1.59E-82	0.953361019	5.81E-78
2	*SNHG3*	245	3.39E-68	0.951868593	1.24E-63
2	*RAD21*	246	3.71E-78	0.947876139	1.36E-73
2	*HNRNPAB*	247	3.45E-98	0.947561604	1.26E-93
2	*FLNA*	248	3.50E-110	0.947306777	1.28E-105
2	*AL627171.2*	249	8.81E-64	0.941399507	3.23E-59
2	*C21orf58*	250	7.31E-62	0.935695812	2.68E-57
2	*CCND2*	251	2.88E-97	0.931243029	1.06E-92
2	*NOLC1*	252	4.42E-74	0.926176316	1.62E-69
2	*ANKRD11*	253	6.40E-77	0.923890415	2.34E-72
2	*PAICS*	254	2.56E-70	0.898229229	9.36E-66
2	*GOLGA8A*	255	5.72E-53	0.897710811	2.09E-48
2	*SMC4*	256	7.71E-69	0.896796913	2.82E-64
2	*NCAPH2*	257	1.06E-79	0.892962502	3.87E-75
2	*LAG3*	258	1.82E-37	0.890425381	6.66E-33
2	*MACF1*	259	1.52E-62	0.884879887	5.58E-58
2	*HNRNPU*	260	4.01E-95	0.884710035	1.47E-90
2	*TUBB*	261	3.91E-94	0.883578405	1.43E-89
2	*HDLBP*	262	6.50E-78	0.883512732	2.38E-73
2	*PHIP*	263	4.30E-65	0.879752038	1.57E-60
2	*ARHGAP30*	264	8.91E-77	0.87758198	3.26E-72
2	*SRSF1*	265	1.65E-77	0.876042503	6.04E-73
2	*GNL3*	266	8.11E-65	0.868517366	2.97E-60
2	*TMPO*	267	1.81E-82	0.852349646	6.64E-78
2	*LMNA*	268	2.26E-29	0.85122563	8.26E-25
2	*SMARCA4*	269	3.87E-74	0.851129944	1.42E-69

2	*HELLS*	270	1.51E-59	0.848726727	5.52E-55
2	*TFRC*	271	6.10E-57	0.845466106	2.23E-52
2	*HSPD1*	272	3.90E-67	0.845049006	1.43E-62
2	*TAF1D*	273	4.31E-53	0.841631321	1.58E-48
2	*MCM7*	274	6.06E-70	0.839097619	2.22E-65
2	*EIF3A*	275	2.17E-93	0.831506973	7.96E-89
2	*C6orf62*	276	3.26E-66	0.828206545	1.19E-61
2	*NFATC2IP*	277	1.46E-44	0.828087134	5.35E-40
2	*KIF20B*	278	2.62E-48	0.82488067	9.59E-44
2	*MCM4*	279	2.11E-62	0.824186754	7.74E-58
2	*SF1*	280	2.06E-73	0.823182802	7.55E-69
2	*PRRC2A*	281	3.21E-70	0.821341897	1.18E-65
2	*SMG1*	282	1.44E-65	0.820023915	5.26E-61
2	*ARGLU1*	283	2.45E-55	0.814094389	8.96E-51
2	*RPS3A*	284	7.88E-120	0.811796704	2.88E-115
2	*DKC1*	285	1.20E-65	0.809868432	4.40E-61
2	*ZBED2*	286	2.72E-45	0.809521623	9.94E-41
2	*PNN*	287	1.23E-67	0.808613787	4.49E-63
2	*HIST1H1E*	288	1.60E-33	0.802746387	5.86E-29
2	*HIST1H1B*	289	1.73E-45	0.79979318	6.32E-41
2	*DYNC1H1*	290	1.96E-60	0.798406431	7.17E-56
2	*SPEN*	291	1.53E-56	0.796662068	5.59E-52
2	*MYH9*	292	4.56E-71	0.796541756	1.67E-66
2	*PTBP1*	293	5.31E-70	0.794601721	1.94E-65
2	*PDS5A*	294	4.04E-64	0.794208806	1.48E-59
2	*VCP*	295	2.22E-85	0.790404067	8.13E-81
2	*CENPE*	296	3.85E-33	0.78769204	1.41E-28
2	*CD320*	297	4.72E-53	0.786062542	1.73E-48
2	*ATAD5*	298	3.09E-47	0.785313219	1.13E-42
2	*BRD4*	299	2.74E-56	0.784084278	1.00E-51
2	*SEC61A1*	300	9.97E-66	0.782324939	3.65E-61
3	*HIST1H4C*	301	1.18E-268	3.174406193	4.31E-264
3	*HIST1H1B*	302	5.07E-279	2.040171164	1.86E-274
3	*HIST1H1C*	303	1.01E-226	1.907128348	3.69E-222
3	*HIST1H3D*	304	2.05E-299	1.881478427	7.50E-295
3	*HIST1H1D*	305	7.44E-197	1.797218792	2.72E-192
3	*HIST2H2AC*	306	2.67E-221	1.769491511	9.77E-217
3	*NUSAP1*	307	1.11E-241	1.717700521	4.07E-237
3	*STMN1*	308	3.76E-203	1.562158154	1.38E-198

3	*HIST1H3B*	309	1.29E-302	1.529958747	4.71E-298
3	*UBE2C*	310	9.56E-251	1.444587622	3.50E-246
3	*H2AFZ*	311	1.18E-221	1.404608072	4.31E-217
3	*HMGB2*	312	4.44E-183	1.397012917	1.62E-178
3	*HIST1H1E*	313	4.05E-184	1.368159634	1.48E-179
3	*TYMS*	314	2.33E-192	1.361205241	8.52E-188
3	*H2AFX*	315	2.40E-192	1.326161626	8.78E-188
3	*HIST1H2AL*	316	5.50E-303	1.324031637	2.01E-298
3	*TUBA1B*	317	7.47E-178	1.315668386	2.73E-173
3	*TOP2A*	318	9.17E-186	1.298846075	3.36E-181
3	*CDKN2D*	319	7.24E-134	1.282076351	2.65E-129
3	*TUBB4B*	320	1.43E-162	1.27449293	5.22E-158
3	*CKS1B*	321	1.16E-173	1.268294547	4.25E-169
3	*RRM2*	322	2.60E-181	1.261473631	9.51E-177
3	*MKI67*	323	1.55E-154	1.212606756	5.66E-150
3	*CDK1*	324	5.61E-210	1.208008941	2.05E-205
3	*TUBB*	325	5.52E-160	1.186005541	2.02E-155
3	*GTSE1*	326	8.51E-208	1.121322803	3.11E-203
3	*DUT*	327	3.55E-114	1.112554868	1.30E-109
3	*HMGN2*	328	7.73E-133	1.072928593	2.83E-128
3	*HIST1H2AG*	329	1.24E-202	1.06463185	4.55E-198
3	*SPC25*	330	1.95E-228	1.032161023	7.14E-224
3	*BIRC5*	331	1.81E-146	1.015731316	6.63E-142
3	*H2AFV*	332	9.65E-127	1.009360407	3.53E-122
3	*CENPM*	333	1.47E-158	1.007482583	5.36E-154
3	*TK1*	334	1.54E-142	1.001634813	5.62E-138
3	*HIST1H2AH*	335	2.84E-231	0.997525853	1.04E-226
3	*CENPF*	336	4.25E-137	0.993540566	1.56E-132
3	*ZWINT*	337	4.93E-151	0.969346745	1.80E-146
3	*CDKN3*	338	9.76E-145	0.967080036	3.57E-140
3	*TPX2*	339	6.15E-138	0.951969029	2.25E-133
3	*UBE2T*	340	6.85E-139	0.947361712	2.51E-134
3	*HIST1H2AJ*	341	5.96E-226	0.928962431	2.18E-221
3	*CKS2*	342	9.04E-117	0.91491941	3.31E-112
3	*HMGB1*	343	7.47E-166	0.884028714	2.74E-161
3	*UBE2S*	344	5.39E-103	0.882876453	1.97E-98
3	*TUBA4A*	345	2.46E-102	0.882475429	8.99E-98
3	*HIST1H2BH*	346	3.09E-192	0.875033191	1.13E-187
3	*PCNA*	347	1.91E-82	0.87421251	6.98E-78

3	*PRC1*	348	2.34E-139	0.86618351	8.55E-135
3	*FBXO5*	349	2.18E-168	0.864586711	7.99E-164
3	*CENPW*	350	7.84E-121	0.862444435	2.87E-116
3	*SAC3D1*	351	5.61E-122	0.843993304	2.05E-117
3	*AURKB*	352	3.41E-158	0.841799116	1.25E-153
3	*CARHSP1*	353	1.61E-107	0.83881587	5.91E-103
3	*PCLAF*	354	9.97E-109	0.830888815	3.65E-104
3	*MXD3*	355	1.25E-202	0.830443856	4.58E-198
3	*MYBL2*	356	3.06E-145	0.83035459	1.12E-140
3	*HIST1H3G*	357	4.29E-226	0.828984595	1.57E-221
3	*ATAD2*	358	4.95E-101	0.826316574	1.81E-96
3	*DEK*	359	4.14E-119	0.817663771	1.51E-114
3	*SMC2*	360	3.52E-101	0.767754908	1.29E-96
3	*H1FX*	361	1.45E-66	0.766120825	5.31E-62
3	*TMPO*	362	6.85E-82	0.764525418	2.51E-77
3	*DTYMK*	363	2.10E-104	0.764095234	7.68E-100
3	*CLSPN*	364	5.46E-88	0.762502831	2.00E-83
3	*CALM2*	365	1.46E-109	0.755526394	5.33E-105
3	*KIF11*	366	1.33E-120	0.749621278	4.87E-116
3	*ESCO2*	367	5.01E-169	0.74917653	1.83E-164
3	*SIVA1*	368	5.62E-93	0.74739768	2.06E-88
3	*SNRNP25*	369	5.62E-84	0.746513454	2.06E-79
3	*SMC4*	370	3.03E-93	0.746019531	1.11E-88
3	*CENPU*	371	3.59E-103	0.734459118	1.31E-98
3	*HIST1H2BF*	372	4.90E-216	0.73227563	1.80E-211
3	*KIFC1*	373	1.21E-147	0.726041332	4.43E-143
3	*CDCA3*	374	4.97E-146	0.725298548	1.82E-141
3	*IDH2*	375	1.40E-71	0.716533031	5.11E-67
3	*CDCA5*	376	2.64E-140	0.716403789	9.66E-136
3	*CDCA8*	377	7.51E-134	0.709663813	2.75E-129
3	*TMEM106C*	378	1.67E-81	0.709412583	6.13E-77
3	*NUDT1*	379	5.13E-81	0.706314759	1.88E-76
3	*CRIP1*	380	2.57E-66	0.701159343	9.39E-62
3	*FAM111A*	381	1.60E-110	0.700836467	5.84E-106
3	*CEP55*	382	1.39E-99	0.692475695	5.07E-95
3	*PBK*	383	1.37E-143	0.69129495	5.01E-139
3	*PTMA*	384	5.81E-113	0.675807211	2.13E-108
3	*CENPA*	385	2.29E-142	0.675481215	8.39E-138
3	*NCAPH*	386	6.34E-100	0.674969823	2.32E-95

3	*KIF2C*	387	1.42E-121	0.673336796	5.21E-117
3	*DNAJC9*	388	8.56E-79	0.665802826	3.13E-74
3	*RHEB*	389	1.57E-70	0.661529425	5.76E-66
3	*NCAPD3*	390	3.88E-85	0.660199007	1.42E-80
3	*TUBA1C*	391	3.47E-70	0.656477683	1.27E-65
3	*NUCKS1*	392	2.31E-87	0.655009017	8.46E-83
3	*NCAPG*	393	5.26E-114	0.651400847	1.92E-109
3	*TACC3*	394	1.22E-91	0.648281166	4.46E-87
3	*SMC3*	395	1.09E-66	0.646474472	3.99E-62
3	*ASPM*	396	2.36E-107	0.6440413	8.65E-103
3	*CKAP2L*	397	3.95E-136	0.643765183	1.45E-131
3	*RPA3*	398	1.45E-73	0.642834627	5.30E-69
3	*MAD2L1*	399	1.24E-78	0.636527921	4.52E-74
3	*HJURP*	400	4.92E-143	0.634022984	1.80E-138
4	*IFITM2*	401	4.11E-82	1.162240989	1.51E-77
4	*BTG1*	402	4.13E-45	0.997548284	1.51E-40
4	*TPT1*	403	1.50E-109	0.915176902	5.48E-105
4	*CD7*	404	1.15E-50	0.895811162	4.20E-46
4	*GZMA*	405	6.10E-34	0.884572481	2.23E-29
4	*RPL10*	406	1.26E-96	0.88051746	4.62E-92
4	*RPS27*	407	1.33E-91	0.859616481	4.89E-87
4	*EEF1A1*	408	8.39E-80	0.84043922	3.07E-75
4	*PRKCQ-AS1*	409	1.59E-42	0.82148471	5.82E-38
4	*C9orf135*	410	4.47E-40	0.816555334	1.64E-35
4	*ID2*	411	1.30E-30	0.759481235	4.75E-26
4	*ALOX5AP*	412	3.38E-35	0.734007509	1.24E-30
4	*RPS28*	413	3.51E-68	0.732356813	1.28E-63
4	*RGS10*	414	7.73E-40	0.724224605	2.83E-35
4	*RPS14*	415	1.79E-72	0.714312226	6.56E-68
4	*RPL30*	416	1.38E-70	0.707060207	5.03E-66
4	*RPS19*	417	1.94E-73	0.703748787	7.09E-69
4	*CD27*	418	2.52E-28	0.678850707	9.23E-24
4	*C15orf48*	419	4.55E-26	0.672314785	1.67E-21
4	*TRAPPC6A*	420	2.61E-31	0.6566907	9.55E-27
4	*RPL34*	421	5.31E-57	0.648034356	1.94E-52
4	*SAMSN1*	422	1.83E-22	0.641657432	6.71E-18
4	*GIMAP7*	423	6.06E-37	0.640347147	2.22E-32
4	*ITM2B*	424	1.09E-39	0.630789011	3.98E-35
4	*OST4*	425	2.41E-46	0.623892419	8.82E-42

4	*RPL39*	426	4.13E-50	0.623179495	1.51E-45
4	*MAL*	427	5.08E-30	0.610108753	1.86E-25
4	*NOP53*	428	2.94E-25	0.609624173	1.08E-20
4	*RPS12*	429	1.78E-57	0.601133639	6.53E-53
4	*SSR4*	430	4.25E-47	0.593533227	1.55E-42
4	*IL4R*	431	5.19E-29	0.589959472	1.90E-24
4	*SPINT2*	432	2.81E-25	0.583024043	1.03E-20
4	*GYPC*	433	6.73E-20	0.575567026	2.46E-15
4	*TXNIP*	434	1.93E-26	0.571025281	7.08E-22
4	*CD48*	435	2.16E-31	0.559863602	7.91E-27
4	*CST7*	436	8.61E-32	0.559100106	3.15E-27
4	*CD37*	437	1.00E-23	0.558296543	3.67E-19
4	*RPL32*	438	9.74E-49	0.558094806	3.56E-44
4	*SOCS1*	439	8.45E-21	0.553951916	3.09E-16
4	*SARAF*	440	1.96E-28	0.544879796	7.19E-24
4	*PFDN5*	441	2.83E-45	0.544506196	1.04E-40
4	*NDFIP2*	442	1.00E-19	0.543589621	3.66E-15
4	*CORO1B*	443	6.43E-15	0.539258991	2.35E-10
4	*GIMAP4*	444	1.46E-26	0.537924896	5.35E-22
4	*RPS13*	445	1.66E-40	0.531726156	6.08E-36
4	*RPS10*	446	1.09E-41	0.530611087	4.00E-37
4	*GBP5*	447	2.66E-12	0.530157701	9.73E-08
4	*FILIP1L*	448	2.86E-20	0.529951778	1.05E-15
4	*PLAC8*	449	6.65E-29	0.527944779	2.43E-24
4	*RPL37*	450	1.09E-54	0.527206605	3.97E-50
4	*EGLN3*	451	6.85E-13	0.525715335	2.51E-08
4	*CSTB*	452	1.64E-30	0.524362537	6.01E-26
4	*PTGIS*	453	1.93E-23	0.524202139	7.08E-19
4	*TNFRSF14*	454	2.06E-20	0.520305418	7.56E-16
4	*PLAAT4*	455	2.93E-39	0.519700815	1.07E-34
4	*CYSLTR1*	456	8.72E-31	0.517630853	3.19E-26
4	*RPL19*	457	6.92E-52	0.517143169	2.53E-47
4	*COMMD6*	458	9.70E-26	0.514790593	3.55E-21
4	*RPL28*	459	1.94E-61	0.514037878	7.09E-57
4	*LGALS3BP*	460	4.46E-16	0.512473098	1.63E-11
4	*LEPROTL1*	461	6.88E-22	0.507902296	2.52E-17
4	*GIMAP1*	462	3.64E-19	0.506474222	1.33E-14
4	*BATF*	463	2.30E-17	0.504004487	8.40E-13
4	*PYCARD*	464	1.30E-22	0.503004007	4.75E-18

4	*PTGER2*	465	3.47E-18	0.50149347	1.27E-13
4	*ATP6V1G1*	466	1.12E-24	0.501179788	4.11E-20
4	*RPLP1*	467	5.68E-52	0.498904306	2.08E-47
4	*TCF7*	468	6.12E-20	0.498598381	2.24E-15
4	*RPL13*	469	3.90E-49	0.497200937	1.43E-44
4	*TRBC2*	470	3.44E-14	0.493100913	1.26E-09
4	*CAPG*	471	8.78E-15	0.492849833	3.21E-10
4	*TOMM7*	472	5.89E-30	0.490448579	2.16E-25
4	*SLC25A6*	473	1.58E-26	0.488872309	5.79E-22
4	*RPL10A*	474	1.15E-39	0.488835384	4.20E-35
4	*TNFSF10*	475	1.35E-09	0.488384364	4.93E-05
4	*RPL11*	476	1.05E-48	0.487729709	3.84E-44
4	*EIF3K*	477	4.34E-29	0.482947507	1.59E-24
4	*GZMM*	478	3.03E-13	0.482106078	1.11E-08
4	*HCST*	479	2.71E-22	0.480915129	9.93E-18
4	*HIGD2A*	480	1.20E-23	0.478898896	4.38E-19
4	*PPIB*	481	1.64E-32	0.475090663	6.00E-28
4	*FAU*	482	4.00E-50	0.469814589	1.46E-45
4	*CYTOR*	483	4.74E-12	0.463775786	1.73E-07
4	*RPS4Y1*	484	3.21E-30	0.463007126	1.18E-25
4	*TRIM22*	485	2.20E-15	0.461645508	8.06E-11
4	*RRS1*	486	4.63E-06	0.460429424	0.169291109
4	*NSMCE1*	487	1.50E-12	0.458635223	5.49E-08
4	*RPS29*	488	5.87E-45	0.458459015	2.15E-40
4	*RPS8*	489	1.82E-38	0.457003125	6.65E-34
4	*IRX3*	490	3.27E-15	0.455942311	1.20E-10
4	*SLFN5*	491	8.53E-19	0.454766129	3.12E-14
4	*RPS15A*	492	3.23E-44	0.453971609	1.18E-39
4	*XBP1*	493	2.94E-21	0.449748737	1.08E-16
4	*ETHE1*	494	4.88E-11	0.448027665	1.79E-06
4	*SMDT1*	495	3.82E-14	0.446049115	1.40E-09
4	*RPL18*	496	1.96E-37	0.445746118	7.18E-33
4	*RPS4X*	497	3.56E-29	0.444274464	1.30E-24
4	*GIMAP5*	498	1.88E-15	0.441710157	6.90E-11
4	*SERF2*	499	3.95E-46	0.439318425	1.44E-41
4	*SATB1*	500	2.99E-14	0.438268863	1.09E-09
5	*DUT*	501	9.75E-42	0.939210571	3.57E-37
5	*SLBP*	502	1.68E-42	0.848633208	6.13E-38
5	*CLSPN*	503	1.51E-41	0.832276527	5.51E-37

5	*GINS2*	504	4.01E-40	0.774533315	1.47E-35
5	*HELLS*	505	9.16E-36	0.737144139	3.35E-31
5	*MCM3*	506	1.51E-32	0.723703218	5.52E-28
5	*GZMA*	507	1.25E-16	0.679599748	4.57E-12
5	*PCNA*	508	1.62E-32	0.671967595	5.94E-28
5	*CENPU*	509	5.17E-28	0.670839034	1.89E-23
5	*MCM6*	510	8.84E-28	0.651414766	3.24E-23
5	*E2F1*	511	4.52E-35	0.639655881	1.65E-30
5	*NASP*	512	2.45E-27	0.629634305	8.98E-23
5	*ATAD2*	513	3.40E-21	0.627701171	1.25E-16
5	*TYMS*	514	1.08E-21	0.612439372	3.95E-17
5	*MCM4*	515	4.49E-28	0.607768265	1.64E-23
5	*FEN1*	516	3.29E-25	0.598832904	1.20E-20
5	*DNMT1*	517	4.65E-24	0.577478186	1.70E-19
5	*MCM5*	518	1.25E-29	0.569894149	4.56E-25
5	*IDH2*	519	4.11E-22	0.565063354	1.51E-17
5	*DHFR*	520	7.38E-21	0.561308238	2.70E-16
5	*DNAJC9*	521	5.35E-20	0.549319244	1.96E-15
5	*SNRNP25*	522	2.01E-18	0.545632283	7.36E-14
5	*WDR76*	523	6.39E-28	0.53546277	2.34E-23
5	*DEK*	524	1.28E-26	0.531559687	4.67E-22
5	*MSH6*	525	1.35E-24	0.52278196	4.94E-20
5	*POLE3*	526	1.59E-17	0.521008408	5.82E-13
5	*CORO1B*	527	9.67E-10	0.511070148	3.54E-05
5	*FAM111B*	528	1.38E-25	0.488326626	5.04E-21
5	*HNRNPF*	529	1.77E-22	0.488161519	6.49E-18
5	*USP1*	530	1.02E-16	0.483984821	3.72E-12
5	*C15orf48*	531	2.89E-09	0.475589287	0.000105806
5	*NDUFB1*	532	2.90E-16	0.472254341	1.06E-11
5	*CDC6*	533	6.73E-24	0.471845416	2.46E-19
5	*GMNN*	534	4.54E-16	0.460537975	1.66E-11
5	*TK1*	535	3.66E-10	0.459761313	1.34E-05
5	*CHAF1A*	536	2.87E-19	0.458815911	1.05E-14
5	*SIVA1*	537	1.92E-14	0.456622759	7.04E-10
5	*ORC6*	538	6.27E-15	0.453410639	2.29E-10
5	*CARHSP1*	539	2.97E-14	0.451458734	1.09E-09
5	*CDT1*	540	5.33E-19	0.446143739	1.95E-14
5	*GIMAP7*	541	1.10E-14	0.439290892	4.02E-10
5	*PSIP1*	542	1.63E-17	0.433752924	5.98E-13

5	*PCLAF*	543	1.22E-11	0.428077076	4.47E-07
5	*NME4*	544	3.31E-14	0.420736743	1.21E-09
5	*ALOX5AP*	545	8.09E-15	0.419357299	2.96E-10
5	*CHEK1*	546	2.11E-15	0.419028479	7.72E-11
5	*BRCA1*	547	1.03E-12	0.415043697	3.77E-08
5	*UNG*	548	1.67E-19	0.414492179	6.10E-15
5	*FTL*	549	1.77E-13	0.408753483	6.49E-09
5	*CENPM*	550	1.69E-12	0.406933746	6.17E-08
5	*MCM7*	551	9.00E-15	0.401194844	3.30E-10
5	*GCHFR*	552	2.08E-11	0.396684103	7.61E-07
5	*MCM2*	553	1.37E-13	0.388718191	5.00E-09
5	*CDC45*	554	4.46E-14	0.386273097	1.63E-09
5	*POLR3K*	555	8.31E-13	0.385266014	3.04E-08
5	*CENPK*	556	7.42E-13	0.383308849	2.71E-08
5	*MAL*	557	8.30E-13	0.382741677	3.04E-08
5	*CDK4*	558	4.78E-12	0.381800774	1.75E-07
5	*TPT1*	559	1.66E-21	0.381412672	6.08E-17
5	*HINT1*	560	2.36E-17	0.380846618	8.65E-13
5	*TMEM106C*	561	1.22E-13	0.373991653	4.46E-09
5	*CENPX*	562	9.25E-10	0.372907952	3.39E-05
5	*CDCA7*	563	8.85E-18	0.371632702	3.24E-13
5	*LTB*	564	4.34E-12	0.371296512	1.59E-07
5	*DTL*	565	8.03E-18	0.369153637	2.94E-13
5	*IL32*	566	2.97E-11	0.368702915	1.09E-06
5	*ARPC3*	567	4.50E-18	0.366553346	1.65E-13
5	*IFITM2*	568	5.19E-10	0.366181913	1.90E-05
5	*RFC4*	569	2.84E-14	0.364129808	1.04E-09
5	*RPA2*	570	2.30E-10	0.358644778	8.42E-06
5	*BARD1*	571	1.12E-09	0.358107812	4.09E-05
5	*LSM2*	572	6.94E-10	0.354342013	2.54E-05
5	*SVIP*	573	1.95E-10	0.353714579	7.15E-06
5	*CSTB*	574	1.47E-11	0.351816747	5.37E-07
5	*RMI2*	575	2.81E-14	0.350241513	1.03E-09
5	*IFI27L1*	576	2.59E-08	0.3494719	0.000946206
5	*PFN1*	577	3.85E-17	0.348799536	1.41E-12
5	*CCNE2*	578	3.64E-20	0.345995291	1.33E-15
5	*ASF1B*	579	2.90E-11	0.345787373	1.06E-06
5	*NR2C2AP*	580	1.53E-12	0.344610281	5.59E-08
5	*RPA3*	581	1.50E-09	0.34439934	5.50E-05

5	*NDUFA6*	582	6.30E-10	0.344193013	2.30E-05
5	*MCM10*	583	1.29E-12	0.343808447	4.71E-08
5	*LIME1*	584	2.46E-10	0.342941176	9.00E-06
5	*PYCARD*	585	6.06E-11	0.342500262	2.22E-06
5	*PRDX2*	586	4.58E-10	0.340135231	1.68E-05
5	*DCTPP1*	587	2.17E-10	0.33902558	7.95E-06
5	*PRKDC*	588	8.06E-09	0.338236983	0.000294985
5	*TALDO1*	589	3.07E-11	0.333127072	1.12E-06
5	*CTSC*	590	1.44E-10	0.332856231	5.25E-06
5	*RANBP1*	591	2.16E-10	0.332271119	7.91E-06
5	*PRIM1*	592	8.25E-12	0.331744508	3.02E-07
5	*RNASEH2C*	593	1.26E-08	0.331627945	0.000461736
5	*LY6E*	594	3.28E-13	0.330651511	1.20E-08
5	*ETHE1*	595	5.72E-09	0.329660506	0.000209356
5	*RFC2*	596	5.15E-10	0.327408084	1.89E-05
5	*SSR4*	597	7.25E-14	0.325604672	2.65E-09
5	*IER3IP1*	598	7.27E-08	0.325211346	0.002660348
5	*SNRPB*	599	1.89E-10	0.324473871	6.91E-06
5	*RPLP1*	600	1.91E-16	0.32321632	6.99E-12
6	*XCL1*	601	2.66E-102	2.503406023	9.75E-98
6	*CD8B*	602	5.50E-183	2.207670897	2.01E-178
6	*CD8A*	603	4.00E-172	2.112428955	1.46E-167
6	*CTSW*	604	1.32E-92	2.111007534	4.84E-88
6	*NKG7*	605	6.59E-68	2.040057891	2.41E-63
6	*GZMA*	606	6.45E-58	1.742289017	2.36E-53
6	*GZMK*	607	5.75E-52	1.488298911	2.10E-47
6	*CD7*	608	1.77E-48	1.450657963	6.49E-44
6	*PRF1*	609	1.97E-42	1.431749461	7.23E-38
6	*HOPX*	610	4.40E-37	1.387169527	1.61E-32
6	*CD27*	611	4.82E-61	1.205526568	1.76E-56
6	*GZMB*	612	3.66E-32	1.184425047	1.34E-27
6	*HCST*	613	8.94E-44	1.181436185	3.27E-39
6	*CCL5*	614	5.92E-22	1.130169466	2.17E-17
6	*PTGIS*	615	9.01E-36	1.077223976	3.30E-31
6	*CXCR6*	616	1.67E-29	1.033145724	6.10E-25
6	*KLRK1*	617	1.13E-88	1.027118375	4.12E-84
6	*STK17B*	618	3.46E-38	0.959121666	1.27E-33
6	*ITGB2*	619	2.70E-25	0.927371252	9.88E-21
6	*BATF*	620	5.17E-28	0.924160322	1.89E-23

6	*PECAM1*	621	5.34E-67	0.863549269	1.95E-62
6	*JAML*	622	5.85E-22	0.856349178	2.14E-17
6	*FXYD2*	623	3.12E-51	0.84286674	1.14E-46
6	*PRKCQ-AS1*	624	1.68E-19	0.837209268	6.14E-15
6	*CTSD*	625	6.87E-16	0.82932404	2.51E-11
6	*GZMH*	626	5.15E-25	0.817648502	1.88E-20
6	*IFITM2*	627	1.53E-26	0.815739506	5.58E-22
6	*TIAM1*	628	4.11E-24	0.804760042	1.50E-19
6	*FILIP1L*	629	1.92E-23	0.800106981	7.03E-19
6	*C15orf48*	630	1.86E-21	0.786166725	6.82E-17
6	*GZMM*	631	2.02E-19	0.785121484	7.38E-15
6	*TOP1*	632	3.20E-21	0.781974244	1.17E-16
6	*LRRN3*	633	1.82E-16	0.779414652	6.65E-12
6	*HAVCR2*	634	5.27E-15	0.760545264	1.93E-10
6	*PAG1*	635	1.13E-17	0.752599857	4.12E-13
6	*GBP5*	636	7.27E-18	0.744007641	2.66E-13
6	*CST7*	637	2.44E-23	0.73694644	8.93E-19
6	*COTL1*	638	6.08E-23	0.73180609	2.23E-18
6	*AIF1*	639	1.27E-22	0.724901946	4.63E-18
6	*TESC*	640	3.47E-16	0.716429169	1.27E-11
6	*CD96*	641	2.56E-15	0.71591541	9.35E-11
6	*GSTP1*	642	1.15E-15	0.715649272	4.19E-11
6	*LCP2*	643	1.06E-15	0.704075079	3.88E-11
6	*GRAP2*	644	9.98E-16	0.703222985	3.65E-11
6	*EVL*	645	3.27E-19	0.702280383	1.20E-14
6	*CXCR3*	646	2.02E-16	0.700900112	7.40E-12
6	*CLEC2B*	647	3.39E-19	0.700512164	1.24E-14
6	*RNF125*	648	1.72E-15	0.694149333	6.31E-11
6	*ABI3*	649	1.35E-15	0.683012828	4.94E-11
6	*GYPC*	650	2.32E-12	0.678496071	8.49E-08
6	*FYB1*	651	5.33E-14	0.674255633	1.95E-09
6	*SLAMF7*	652	2.93E-52	0.673977751	1.07E-47
6	*RFLNB*	653	3.36E-19	0.666856868	1.23E-14
6	*IL16*	654	2.30E-16	0.664754151	8.44E-12
6	*HTATSF1*	655	7.00E-06	0.664641117	0.256074043
6	*KRT7*	656	2.28E-06	0.662408636	0.083522555
6	*STK17A*	657	7.81E-16	0.649437444	2.86E-11
6	*ID2*	658	6.87E-15	0.648183792	2.52E-10
6	*SERPINB9*	659	8.35E-13	0.64241378	3.06E-08

6	*XBP1*	660	2.92E-16	0.641265137	1.07E-11
6	*CAPG*	661	2.28E-16	0.639376966	8.36E-12
6	*MT1F*	662	3.82E-10	0.637987464	1.40E-05
6	*HIF1A*	663	2.24E-12	0.631226633	8.18E-08
6	*DUSP6*	664	7.50E-06	0.629829334	0.274352793
6	*TXNIP*	665	1.54E-16	0.629170378	5.63E-12
6	*SPINT2*	666	1.68E-14	0.62437471	6.16E-10
6	*FCER1G*	667	2.65E-27	0.624270816	9.69E-23
6	*EOMES*	668	3.83E-23	0.621866312	1.40E-18
6	*KLF13*	669	3.24E-18	0.621420893	1.18E-13
6	*MAL*	670	2.47E-17	0.621254983	9.05E-13
6	*IL18R1*	671	3.14E-11	0.620517308	1.15E-06
6	*LAG3*	672	9.38E-08	0.619304604	0.003434963
6	*BTG1*	673	1.70E-17	0.617229142	6.23E-13
6	*RPS19*	674	7.49E-31	0.615215753	2.74E-26
6	*GAB3*	675	1.57E-19	0.609462071	5.76E-15
6	*ITGA1*	676	2.13E-20	0.606330768	7.80E-16
6	*SLFN5*	677	1.93E-13	0.594682713	7.07E-09
6	*ANXA1*	678	6.63E-07	0.580266222	0.024266053
6	*PTPRC*	679	2.31E-19	0.579919836	8.44E-15
6	*DPP4*	680	5.00E-12	0.579480793	1.83E-07
6	*FKBP11*	681	9.08E-11	0.576458626	3.32E-06
6	*CSF1*	682	1.43E-06	0.573680582	0.05250333
6	*NELL2*	683	3.18E-38	0.569834261	1.16E-33
6	*RIN3*	684	1.74E-28	0.568126131	6.37E-24
6	*RGS10*	685	7.28E-13	0.5635006	2.66E-08
6	*CD3D*	686	6.92E-23	0.563149554	2.53E-18
6	*RCBTB2*	687	5.03E-10	0.560913998	1.84E-05
6	*NCR3*	688	4.08E-09	0.560459091	0.000149434
6	*CD3G*	689	8.34E-18	0.557871991	3.05E-13
6	*CD55*	690	2.76E-07	0.55490959	0.010112882
6	*DOCK10*	691	4.48E-07	0.548823254	0.016380898
6	*CD44*	692	3.93E-11	0.544511957	1.44E-06
6	*PIK3CD*	693	1.36E-08	0.539434338	0.000497757
6	*THEMIS*	694	8.81E-09	0.538287186	0.000322284
6	*SYNRG*	695	4.09E-11	0.53755037	1.50E-06
6	*CDCP1*	696	1.89E-19	0.536705947	6.93E-15
6	*ITM2B*	697	8.45E-11	0.530176594	3.09E-06
6	*CD52*	698	2.99E-17	0.529573627	1.10E-12

6	*LAPTM5*	699	1.90E-09	0.525952186	6.95E-05
6	*IL4R*	700	3.25E-08	0.525075374	0.00119028
7	*ARL6IP1*	701	5.10E-68	2.076667386	1.87E-63
7	*UBE2C*	702	9.05E-75	2.066551006	3.31E-70
7	*CENPF*	703	1.87E-64	1.848308681	6.84E-60
7	*CCNB1*	704	9.02E-86	1.68173229	3.30E-81
7	*ASPM*	705	1.37E-65	1.658638302	5.01E-61
7	*KPNA2*	706	8.08E-61	1.650413494	2.96E-56
7	*HMGB2*	707	6.67E-58	1.649690092	2.44E-53
7	*CKS2*	708	1.06E-61	1.64361771	3.90E-57
7	*TUBB4B*	709	5.06E-59	1.643024964	1.85E-54
7	*TOP2A*	710	7.36E-56	1.639896108	2.69E-51
7	*CDC20*	711	3.58E-77	1.509491526	1.31E-72
7	*CKS1B*	712	1.13E-58	1.448929461	4.15E-54
7	*CCNB2*	713	1.04E-82	1.423864593	3.80E-78
7	*CDKN3*	714	2.57E-65	1.389753543	9.40E-61
7	*PLK1*	715	3.07E-89	1.376831379	1.12E-84
7	*PTTG1*	716	5.47E-57	1.371229512	2.00E-52
7	*UBALD2*	717	9.69E-53	1.367511561	3.55E-48
7	*UBE2S*	718	3.03E-48	1.357542415	1.11E-43
7	*BIRC5*	719	3.45E-61	1.354822974	1.26E-56
7	*TUBA1C*	720	7.63E-46	1.34298722	2.79E-41
7	*TPX2*	721	5.56E-51	1.327791827	2.03E-46
7	*GTSE1*	722	5.18E-59	1.275483504	1.90E-54
7	*MKI67*	723	4.33E-40	1.240899288	1.58E-35
7	*TROAP*	724	5.85E-81	1.236191228	2.14E-76
7	*NUSAP1*	725	1.59E-45	1.231211726	5.84E-41
7	*NUF2*	726	4.35E-59	1.177215553	1.59E-54
7	*DLGAP5*	727	2.43E-57	1.157544783	8.88E-53
7	*CEP55*	728	3.36E-52	1.135911431	1.23E-47
7	*JPT1*	729	4.31E-50	1.128110802	1.58E-45
7	*PRC1*	730	2.43E-46	1.122055394	8.88E-42
7	*HMGN2*	731	2.08E-45	1.112113041	7.61E-41
7	*CDCA8*	732	2.11E-55	1.111002479	7.73E-51
7	*CALM2*	733	5.12E-42	1.089211711	1.87E-37
7	*CENPE*	734	2.68E-48	1.088816255	9.79E-44
7	*KIF2C*	735	2.97E-64	1.078852622	1.09E-59
7	*CDK1*	736	3.81E-38	1.076138261	1.40E-33
7	*AURKA*	737	1.02E-51	1.061034758	3.73E-47

7	*HMMR*	738	6.57E-63	1.025969609	2.40E-58
7	*SMC4*	739	1.02E-37	1.021543606	3.73E-33
7	*CKAP2*	740	6.17E-44	1.011400927	2.26E-39
7	*KIF20B*	741	3.75E-43	0.996329089	1.37E-38
7	*HP1BP3*	742	5.71E-36	0.975958035	2.09E-31
7	*SGO2*	743	3.76E-47	0.975318919	1.37E-42
7	*H2AFV*	744	1.97E-37	0.95778851	7.20E-33
7	*STMN1*	745	2.67E-32	0.905758676	9.76E-28
7	*DCTN3*	746	4.29E-35	0.903635499	1.57E-30
7	*CENPA*	747	3.01E-56	0.898757247	1.10E-51
7	*CDC25B*	748	8.20E-32	0.896484344	3.00E-27
7	*CENPW*	749	3.32E-37	0.890663133	1.21E-32
7	*TUBA1B*	750	1.50E-30	0.885674408	5.50E-26
7	*NEK2*	751	1.43E-99	0.870877101	5.22E-95
7	*HMGB3*	752	3.18E-38	0.870219496	1.17E-33
7	*TUBA1A*	753	9.67E-27	0.86799748	3.54E-22
7	*SDCBP*	754	5.23E-33	0.864275839	1.92E-28
7	*KLF2*	755	1.42E-16	0.8456706	5.20E-12
7	*KIF14*	756	7.64E-60	0.841716541	2.80E-55
7	*NUCKS1*	757	4.46E-38	0.836931655	1.63E-33
7	*VIM*	758	3.31E-27	0.817241353	1.21E-22
7	*CDCA3*	759	5.22E-47	0.815778585	1.91E-42
7	*LGALS1*	760	9.81E-19	0.813980174	3.59E-14
7	*UGP2*	761	2.12E-24	0.799032369	7.77E-20
7	*PRR11*	762	4.26E-45	0.775025967	1.56E-40
7	*DEPDC1*	763	6.47E-71	0.759926031	2.37E-66
7	*KIF23*	764	3.91E-46	0.74544361	1.43E-41
7	*KNSTRN*	765	4.21E-51	0.742025001	1.54E-46
7	*CDKN2D*	766	4.96E-17	0.73522491	1.82E-12
7	*MXD3*	767	2.28E-43	0.728547399	8.36E-39
7	*TUBB*	768	8.92E-25	0.725678789	3.26E-20
7	*LSM5*	769	9.83E-26	0.719051909	3.60E-21
7	*TACC3*	770	8.09E-31	0.719029247	2.96E-26
7	*KIF11*	771	2.79E-29	0.717150789	1.02E-24
7	*BUB1*	772	4.73E-41	0.715206047	1.73E-36
7	*CCNA2*	773	4.80E-37	0.709470816	1.76E-32
7	*AURKB*	774	3.35E-34	0.707254365	1.23E-29
7	*KRAS*	775	1.61E-21	0.707211268	5.91E-17
7	*HJURP*	776	1.81E-34	0.694028013	6.62E-30

7	*SPTBN1*	777	5.49E-25	0.69040776	2.01E-20
7	*CDK2AP2*	778	3.13E-26	0.688102292	1.15E-21
7	*SGO1*	779	9.15E-33	0.684511798	3.35E-28
7	*H2AFZ*	780	4.35E-23	0.680828061	1.59E-18
7	*COX8A*	781	4.98E-30	0.674090107	1.82E-25
7	*KIF4A*	782	1.60E-47	0.672424019	5.84E-43
7	*H2AFX*	783	2.32E-16	0.671207229	8.48E-12
7	*RAD21*	784	7.42E-22	0.670123818	2.72E-17
7	*RAC2*	785	2.47E-28	0.654844472	9.06E-24
7	*CKAP2L*	786	1.70E-29	0.649616386	6.23E-25
7	*MAD2L1*	787	8.74E-25	0.640858924	3.20E-20
7	*PIF1*	788	9.62E-39	0.639892913	3.52E-34
7	*BUB3*	789	2.77E-18	0.63472108	1.01E-13
7	*REEP4*	790	4.94E-32	0.632737451	1.81E-27
7	*ARHGAP11A*	791	8.34E-27	0.63202751	3.05E-22
7	*SMC2*	792	1.34E-21	0.629531842	4.89E-17
7	*DTYMK*	793	1.75E-20	0.624802657	6.39E-16
7	*SUN2*	794	1.99E-20	0.622933215	7.30E-16
7	*TMPO*	795	1.31E-17	0.621140963	4.80E-13
7	*KNL1*	796	2.27E-28	0.617533917	8.29E-24
7	*EMC9*	797	2.60E-26	0.614645573	9.53E-22
7	*GRK6*	798	3.01E-17	0.608878699	1.10E-12
7	*KIF20A*	799	5.40E-68	0.608375734	1.98E-63
7	*HMG20B*	800	5.52E-26	0.604360357	2.02E-21

Supplemental Videos 1-3 Continuous monitoring of killing of MEC1 cells by nt-KO CD19-CAR-T cells or ITK-KO CD19-CAR-T cells in vitro. CAR-T cells were sorted by GFP$^+$. MEC1 cells were cocultured with/without CAR-T cells at E:T ratio of 2:1 for 48 hours. CellEvent™ Caspase 3/7 Red Detection Reagent was used for indicating the apoptosis of cells. Incucyte® Live-Cell Analysis (Sartorius) was used for continuous monitoring.

Supplemental Video 1 Continuous monitoring of killing of MEC1 cells by nt-KO CD19-CAR-T cells in vitro

Supplemental Video 2 Continuous monitoring of killing of MEC1 cells by ITK-KO CD19-CAR-T cells in vitro

Supplemental Video 3 Continuous monitoring of MEC1 cells without co-culture with CAR-T cells in vitro
