# Supplementary material for: IL-2–inducible T cell kinase deficiency sustains chimeric antigen receptor T cell therapy against tumor cells
Source: J Clin Invest. 2024 Nov 26;135(4):e178558. doi: 10.1172/JCI178558 (PMC11827851; doi:10.1172/JCI178558)

Full unedited blot for **Figure 1D**
The bands in green frame were selected for **Figure 1D**

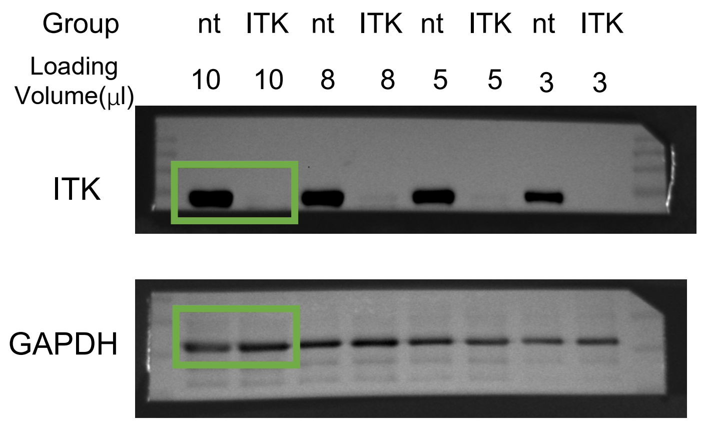

Full unedited blot for **Supplemental Figure 1L**
The bands in red frame were selected for **Supplemental Figure 1L**

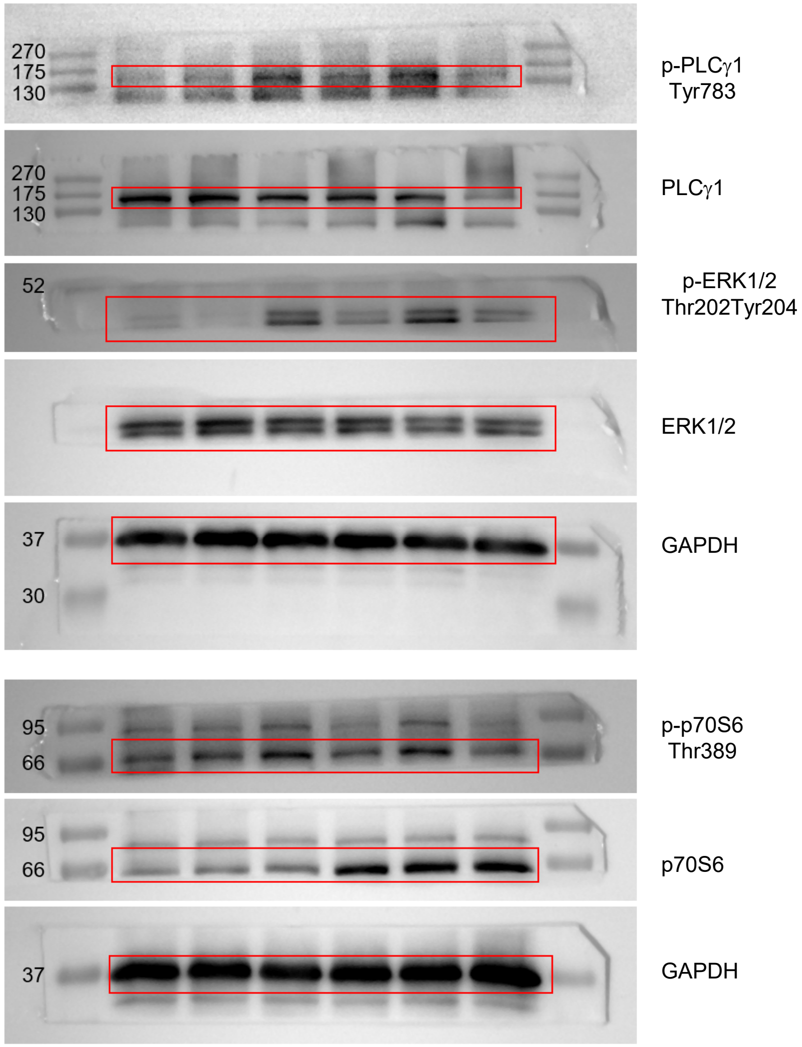

Supplement: Unedited blot and gel images [file jci-135-178558-s241.pdf]
